# Supplementary material for: Triterpene Glycosides from the Far Eastern Sea Cucumber Thyonidium (=Duasmodactyla) kurilensis (Levin): The Structures, Cytotoxicities, and Biogenesis of Kurilosides A3, D1, G, H, I, I1, J, K, and K1
Source: Mar Drugs. 2021 Mar 27;19(4):187. doi: 10.3390/md19040187 (PMC8066294; doi:10.3390/md19040187)
Supplement: Supplementary file 1 [file marinedrugs-19-00187-s001.pdf]

Supplementary data content page

Title: Triterpene glycosides from the Far Eastern sea cucumber *Thyonidium (=Duasmodactyla) kurilensis* (Levin): the structures, cytotoxicities and biogenesis of Kurilosides A_3, D_1, G, H, I, I_1, J, K and K_1

Authors: Alexandra S. Silchenko*[1], Anatoly I. Kalinovsky[1], Sergey A. Avilov[1], Pelageya V. Andrijaschenko[1], Roman S. Popov[1], Pavel S. Dmitrenok, Ekaterina A. Chingizova[1] and Vladimir I. Kalinin[1]

Address: [1]*G.B. Elyakov Pacific Institute of Bioorganic Chemistry, Far Eastern Branch of Russian Academy of Sciences, Pr. 100-let Vladivostoku 159, 690022 Vladivostok, Russia*

Correspondence: kalininv@piboc.dvo.ru; Tel.: +7-914-705-0845

Contents:

Table S1. ^{13}C and ^{1}H NMR chemical shifts, HMBC and ROESY correlations of the carbohydrate moiety of kuriloside A_3 (**1**).

Table S2. ^{13}C and ^{1}H NMR chemical shifts, HMBC and ROESY correlations of the aglycone moiety of kurilosides A_3 (**1**) and G (**3**).

Table S3. ^{13}C and ^{1}H NMR chemical shifts, HMBC and ROESY correlations of the carbohydrate moiety of kuriloside D_1 (**2**).

Table S4. ^{13}C and ^{1}H NMR chemical shifts, HMBC and ROESY correlations of the aglycone moiety of kurilosides H (**4**) and I_1 (**6**).

Table S5. ^{13}C and ^{1}H NMR chemical shifts, HMBC and ROESY correlations of the carbohydrate moiety of DS-kuriloside L (**10**).

Table S6. ^{13}C and ^{1}H NMR chemical shifts, HMBC and ROESY correlations of the aglycone moiety of DS-kuriloside L (**10**).

Table S7. ^{13}C and ^{1}H NMR chemical shifts, HMBC and ROESY correlations of the carbohydrate moiety of DS-kuriloside M (**11**).

Table S8. ^{13}C and ^{1}H NMR chemical shifts, HMBC and ROESY correlations of the aglycone moiety of DS-kuriloside M (**11**).

Figure S1. The ^{1}H NMR (700.00 MHz) and ^{13}C NMR (176.03 MHz) spectra of kuriloside A_3 (**1**) in C_5D_5N/D_2O (4/1).

Figure S2. The COSY (700.00 MHz) spectrum of kuriloside A_3 (**1**) in C_5D_5N/D_2O (4/1).

Figure S3. The HSQC (700.00 MHz) spectrum of kuriloside A_3 (**1**) in C_5D_5N/D_2O (4/1).

Figure S4. The ROESY (700.00 MHz) spectrum of kuriloside A_3 (**1**) in C_5D_5N/D_2O (4/1).

Figure S5. The HMBC (700.00 MHz) spectrum of kuriloside A_3 (**1**) in C_5D_5N/D_2O (4/1).

Figure S6. HR-ESI-MS and ESI-MS/MS spectra of kuriloside A_3 (**1**).

Figure S7. The ^{13}C NMR (176.03 MHz) spectrum of kuriloside D_1 (**2**) in C_5D_5N/D_2O (4/1).

Figure S8. The ^{1}H NMR (700.00 MHz) spectrum of kuriloside D_1 (**2**) in C_5D_5N/D_2O (4/1).

Figure S9. The COSY (700.00 MHz) spectrum of kuriloside D_1 (**2**) in C_5D_5N/D_2O (4/1).

Figure S10. The HSQC (700.00 MHz) spectrum of kuriloside D_1 (**2**) in C_5D_5N/D_2O (4/1).

Figure S11. The HMBC (700.00 MHz) spectrum of kuriloside D_1 (**2**) in C_5D_5N/D_2O (4/1).

Figure S12. The ROESY (700.00 MHz) spectrum of kuriloside D_1 (**2**) in C_5D_5N/D_2O (4/1).

Figure S13. HR-ESI-MS and ESI-MS/MS spectra of kuriloside D_1 (**2**).

Figure S14. The ^{13}C NMR (176.03 MHz) spectrum of kuriloside G (**3**) in C_5D_5N/D_2O (4/1).

Figure S15. The ^{1}H NMR (700.00 MHz) spectrum of kuriloside G (**3**) in C_5D_5N/D_2O (4/1).

Figure S16. The COSY (700.00 MHz) spectrum of kuriloside G (**3**) in C_5D_5N/D_2O (4/1).

Figure S17. The HSQC (700.00 MHz) spectrum of kuriloside G (**3**) in C_5D_5N/D_2O (4/1).

Figure S18. The HMBC (700.00 MHz) spectrum of kuriloside G (**3**) in C_5D_5N/D_2O (4/1).

Figure S19. The ROESY (700.00 MHz) spectrum of kuriloside G (**3**) in C_5D_5N/D_2O (4/1).

Figure S20. 1 D TOCSY (700.00 MHz) spectra of the XyloseI, QuinovoseII and GlucoseIII of kuriloside G (**3**), C_5D_5N/D_2O (4/1).

Figure S21. 1 D TOCSY (700.00 MHz) spectra of the MeGlcIV, GlucoseV and MeGlcVI of kuriloside G (**3**), C_5D_5N/D_2O (4/1).

Figure S22. HR-ESI-MS and ESI-MS/MS spectra of kuriloside G (**3**).

Figure S23. The ^{13}C NMR (176.03 MHz) spectrum of kuriloside H (**4**) in C_5D_5N/D_2O (4/1).
Figure S24. The ^{1}H NMR (700.00 MHz) spectrum of kuriloside H (**4**) in C_5D_5N/D_2O (4/1).
Figure S25. The COSY (700.00 MHz) spectrum of kuriloside H (**4**) in C_5D_5N/D_2O (4/1).
Figure S26. The HSQC (700.00 MHz) spectrum of kuriloside H (**4**) in C_5D_5N/D_2O (4/1).
Figure S27. The ROESY (700.00 MHz) spectrum of kuriloside H (**4**) in C_5D_5N/D_2O (4/1).
Figure S28. The HMBC (700.00 MHz) spectrum of kuriloside H (**4**) in C_5D_5N/D_2O (4/1).
Figure S29. 1 D TOCSY (700.00 MHz) spectra of XyloseI, QuinovoseII and GlucoseIII of kuriloside H (**4**), C_5D_5N/D_2O (4/1).
Figure S30. 1 D TOCSY (700.00 MHz) spectra of GlcIV, GlucoseV and MeGlcVI of kuriloside H (**4**) in C_5D_5N/D_2O (4/1).
Figure S31. HR-ESI-MS and ESI-MS/MS spectra of kuriloside H (**4**).
Figure S32. The ^{13}C NMR (176.03 MHz) spectrum of kuriloside I (**5**) in C_5D_5N/D_2O (4/1).
Figure S33. The ^{1}H NMR (700.00 MHz) spectrum of kuriloside I (**5**) in C_5D_5N/D_2O (4/1).
Figure S34. The COSY (700.00 MHz) spectrum of kuriloside I (**5**) in C_5D_5N/D_2O (4/1).
Figure S35. The HSQC (700.00 MHz) spectrum of kuriloside I (**5**) in C_5D_5N/D_2O (4/1).
Figure S36. The HMBC (700.00 MHz) spectrum of kuriloside I (**5**) in C_5D_5N/D_2O (4/1).
Figure S37. The ROESY (700.00 MHz) spectrum of kuriloside I (**5**) in C_5D_5N/D_2O (4/1).
Figure S38. 1D TOCSY (700.00 MHz) spectra of XyloseI, QuinovoseII and GlucoseIII kuriloside I (**5**) in C_5D_5N/D_2O (4/1).
Figure S39. 1D TOCSY (700.00 MHz) spectra of GlucoseIV and MeGlcV of kuriloside I (**5**), C_5D_5N/D_2O (4/1).
Figure S40. HR-ESI-MS and ESI-MS/MS spectra of kuriloside I (**5**).
Figure S41. The ^{13}C NMR (176.03 MHz) spectrum of kuriloside I_1 (**6**) in C_5D_5N/D_2O (4/1).
Figure S42. The ^{1}H NMR (700.00 MHz) spectrum of kuriloside I_1 (**6**) in C_5D_5N/D_2O (4/1).
Figure S43. The COSY (700.00 MHz) spectrum of kuriloside I_1 (**6**) in C_5D_5N/D_2O (4/1).
Figure S44. The HSQC (700.00 MHz) spectrum of kuriloside I_1 (**6**) in C_5D_5N/D_2O (4/1).
Figure S45. The ROESY (700.00 MHz) spectrum of kuriloside I_1 (**6**) in C_5D_5N/D_2O (4/1).
Figure S46. The HMBC (700.00 MHz) spectrum of kuriloside I_1 (**6**) in C_5D_5N/D_2O (4/1).
Figure S47. HR-ESI-MS and ESI-MS/MS spectra of kuriloside I_1 (**6**).
Figure S48. The ^{13}C NMR (176.03 MHz) spectrum of kuriloside J (**7**) in C_5D_5N/D_2O (4/1).
Figure S49. The ^{1}H NMR (700.00 MHz) spectrum of kuriloside J (**7**) in C_5D_5N/D_2O (4/1).
Figure S50. The COSY (700.00 MHz) spectrum of kuriloside J (**7**) in C_5D_5N/D_2O (4/1).
Figure S51. The HSQC (700.00 MHz) spectrum of kuriloside J (**7**) in C_5D_5N/D_2O (4/1).
Figure S52. The HMBC (700.00 MHz) spectrum of kuriloside J (**7**) in C_5D_5N/D_2O (4/1).
Figure S53. The ROESY (700.00 MHz) spectrum of kuriloside J (**7**) in C_5D_5N/D_2O (4/1).
Figure S54. 1 D TOCSY (700.00 MHz) spectra of XyloseI, QuinovoseII and GlucoseIII of kuriloside J (**7**) in C_5D_5N/D_2O (4/1).
Figure S55. 1 D TOCSY (700.00 MHz) spectra of GlcIV and MeGlcV of kuriloside J (**7**) in C_5D_5N/D_2O (4/1)
Figure S56. HR-ESI-MS and ESI-MS/MS spectra of kuriloside J (**7**).
Figure S57. The ^{13}C NMR (176.03 MHz) spectrum of kuriloside K (**8**) in C_5D_5N/D_2O (4/1).
Figure S58. The ^{1}H NMR (700.00 MHz) spectrum of kuriloside K (**8**) in C_5D_5N/D_2O (4/1).
Figure S59. The COSY (700.00 MHz) spectrum of kuriloside K (**8**) in C_5D_5N/D_2O (4/1).
Figure S60. The HSQC (700.00 MHz) spectrum of kuriloside K (**8**) in C_5D_5N/D_2O (4/1).
Figure S61. The HMBC (700.00 MHz) spectrum of kuriloside K (**8**) in C_5D_5N/D_2O (4/1).
Figure S62. The ROESY (700.00 MHz) spectrum of kuriloside K (**8**) in C_5D_5N/D_2O (4/1).
Figure S63. 1 D TOCSY (700.00 MHz) spectra of XyloseI, QuinovoseII and GlucoseIII of kuriloside K (**8**) in C_5D_5N/D_2O (4/1).
Figure S64. 1 D TOCSY (700.00 MHz) spectra of GlcIV and MeGlcV of kuriloside K (**8**) in C_5D_5N/D_2O (4/1).
Figure S65. HR-ESI-MS and ESI-MS/MS spectra of kuriloside K (**8**).
Figure S66. The ^{13}C NMR (176.03 MHz) spectrum of kuriloside K_1 (**9**) in C_5D_5N/D_2O (4/1).
Figure S67. The ^{1}H NMR (700.00 MHz) spectrum of kuriloside K_1 (**9**) in C_5D_5N/D_2O (4/1).
Figure S68. HR-ESI-MS and ESI-MS/MS spectra of kuriloside K_1 (**9**).
Figure S69. The ^{13}C NMR (176.03 MHz) spectrum of DS-kuriloside L (**10**) in C_5D_5N.
Figure S70. The ^{1}H NMR (700.00 MHz) spectrum of DS-kuriloside L (**10**) in C_5D_5N.

Figure S71. The COSY (700.00 MHz) spectrum of DS-kuriloside L (**10**) in C_5D_5N.
Figure S72. The HSQC (700.00 MHz) spectrum of DS-kuriloside L (**10**) in C_5D_5N.
Figure S73. The HMBC (700.00 MHz) spectrum of DS-kuriloside L (**10**) in C_5D_5N.
Figure S74. The ROESY (700.00 MHz) spectrum of DS-kuriloside L (**10**) in C_5D_5N.
Figure S75. 1 D TOCSY (700.00 MHz) spectra of XyloseI, QuinovoseII and GlucoseIII of DS-kuriloside L (**10**) in C_5D_5N.
Figure S76. HR-ESI-MS (−) and ESI-MS/MS spectra of DS-kuriloside L (**10**).
Figure S77. The ^{13}C NMR (176.03 MHz) spectrum of DS-kuriloside M (**11**) in C_5D_5N.
Figure S78. The ^{1}H NMR (700.00 MHz) spectrum of DS-kuriloside M (**11**) in C_5D_5N.
Figure S79. The COSY (700.00 MHz) spectrum of DS-kuriloside M (**11**) in C_5D_5N.
Figure S80. The HSQC (700.00 MHz) spectrum of DS-kuriloside M (**11**) in C_5D_5N.
Figure S81. The HMBC (700.00 MHz) spectrum of DS-kuriloside M (**11**) in C_5D_5N.
Figure S82. The ROESY (700.00 MHz) spectrum of DS-kuriloside M (**11**) in C_5D_5N.
Figure S83. 1 D TOCSY (700.00 MHz) spectra of XyloseI, QuinovoseII and GlucoseIII of DS-kuriloside M (**11**) in C_5D_5N.
Figure S84. 1 D TOCSY (700.00 MHz) spectra of GlcIV and MeGlcV of DS-kuriloside M (**11**) in C_5D_5N.
Figure S85. HR-ESI-MS (−) and ESI-MS/MS spectra of DS-kuriloside M (**11**).

Table S1. ^{13}C and ^{1}H NMR chemical shifts, HMBC and ROESY correlations of the carbohydrate moiety of kuriloside A_3 (**1**).

Atom.	δ_C mult.[a, b, c]	δ_H mult.[d] (*J* in Hz)	HMBC	ROESY
Xyl1 (1→C-3)				
1	104.7 CH	4.64 d (7.1)	C-3	H-3; H-3, 5 Xyl1
2	**82.0** CH	3.95 t (7.1)	C: 1 Qui2; C: 1, 3 Xyl1	H-1 Qui2
3	75.1 CH	4.15 t (7.9)	C: 2, 4 Xyl1	H-1 Xyl1
4	**78.0** CH	4.13 t (7.9)	C: 1 Glc4; C: 3 Xyl1	H-1 Glc4
5	63.5 CH_2	4.36 dd (5.0; 11.4)	C: 3, 4 Xyl1	
		3.61 m		
Qui2 (1→2Xyl1)				
1	104.5 CH	5.03 d (7.6)	C: 2 Xyl1	H-2 Xyl1; H-3, 5 Qui2
2	75.6 CH	3.88 t (8.4)	C: 1, 3 Qui2	H-4 Qui2
3	75.1 CH	4.00 t (8.4)	C: 2, 4 Qui2	H-1, 5 Qui2
4	**86.3** CH	3.53 t (8.4)	C: 1 Glc3; C: 3, 5 Qui2	H-1 Glc3; H-2 Qui2
5	71.4 CH	3.70 dd (5.9; 9.3)		H-1 Qui2
6	17.9 CH_3	1.62 d (5.9)	C: 4, 5 Qui2	
Glc3 (1→4Qui2)				
1	104.6 CH	4.81 d (7.5)	C: 4 Qui2	H-4 Qui2; H-3,5 Glc3
2	74.3 CH	3.87 t (8.7)	C: 1, 3 Glc3	
3	77.6 CH	4.13 t (8.7)	C: 4 Glc3	H-1 Glc3
4	70.9 CH	3.93 t (8.7)	C: 5 Glc3	
5	77.5 CH	3.92 t (8.7)	C: 6 Glc3	H-1, 3 Glc3
6	61.8 CH_2	4.39 d (12.4)		
		4.06 dd (5.0; 12.4)	C: 5 Glc3	
Glc4 (1→4Xyl1)				
1	102.3 CH	4.87 d (7.5)	C: 4 Xyl1	H-4 Xyl1; H-3, 5 Glc4
2	73.2 CH	3.83 t (8.7)	C: 1, 3 Glc4	
3	**86.0** CH	4.15 t (8.7)	C: 2, 4 Glc4, C: 1 MeGlc5	H-1 MeGlc5; H-1, 5 Glc4
4	69.2 CH	3.88 t (8.7)	C: 3, 5, 6 Glc4	
5	75.1 CH	4.02 t (8.7)		H-1, 3 Glc4
6	*67.2* CH_2	4.93 brd (11.2)	C: 4, 5 Glc4	
		4.66 dd (6,0; 11.2)	C: 5 Glc4	
MeGlc5 (1→3Glc4)				
1	104.4 CH	5.18 d (7.5)	C: 3 Glc4	H-3 Glc4; H-3, 5 MeGlc5
2	74.5 CH	3.83 t (8.6)	C: 1 MeGlc5	
3	86.9 CH	3.65 t (8.6)	C: 2, 4 MeGlc5, OMe	H-1, 5 MeGlc5
4	70.3 CH	3.89 t (8.6)	C: 3 MeGlc5	H-6 MeGlc6
5	77.3 CH	3.89 t (8.6)	C: 4 MeGlc5	H-1, 3 MeGlc5
6	61.7 CH_2	4.34 d (12.8)	C: 4, 5 MeGlc5	
		4.05 dd (6.4; 12.8)	C: 5 MeGlc5	
OMe	60.6 CH_3	3.79 s	C: 3 MeGlc5	

[a]Recorded at 176.03 MHz in C_5D_5N/D_2O (4/1). [b]Bold = interglycosidic positions. [c]Italic = sulphate position. [d]Recorded at 700.00 MHz in C_5D_5N/D_2O (4/1). Multiplicity by 1D TOCSY.

Table S2. ^{13}C and ^{1}H NMR chemical shifts, HMBC and ROESY correlations of the aglycone of kurilosides A_3 (**1**) and G (**3**).

Position	δ_C mult.[a]	δ_H mult. (*J* in Hz)[b]	HMBC	ROESY
1	36.2 CH_2	1.77 m		H-11
		1.41 m		H-3, H-5, H-11, H-31
2	27.0 CH_2	2.19 m		
		1.94 m		
3	88.4 CH	3.19 dd (4.6; 12.5)	C: 4, 30, 31, C:1 Xyl1	H-1, H-5, H-31, H1-Xyl1
4	39.7 C			
5	52.8 CH	0.92 brd (12.5)	C: 4, 10, 19, 30	H-1, H-3, H-7, H-31
6	21.2 CH_2	1.70 m		H-31
		1.47 m		H-19
7	28.4 CH_2	1.64 m		
		1.36 m		
8	41.5 CH	2.16 m		H-18, H-19
9	149.0 C			
10	39.4 C			
11	114.2 CH	5.31 brd (6.3)	C: 8, 10, 12, 13	H-1
12	35.8 CH_2	2.48 brd (16.1)		H-17, H-32
		1.94 dd (6.3; 16.1)	C: 9, 11, 13, 18	H-8, H-18
13	46.9 C			
14	46.8 C			
15	45.2 CH_2	2.07 dd (9.2; 13.2) β	C: 8, 13, 32	H-16, H-18
		1.79 d (13.2) α	C: 14, 16, 32	H-32
16	71.1 CH	5.40 brt (7.5)	C: 13, 14, 20	H-15β, H-18
17	70.0 CH	3.40 d (6.4)	C: 12, 13, 14, 16, 18, 20	H-12, H-21, H-32
18	17.3 CH_3	0.71 s	C: 12, 13, 17	H-8, H-12, H-15β, H-16, H-19
19	22.3 CH_3	1.10 s	C: 1, 5, 9, 10	H-1, H-2, H-6, H-8, H-18
20	208.8 C			
21	31.2 CH_3	2.18 s	C: 17, 20	H-12, H-17
30	16.5 CH_3	1.05 s	C: 3, 4, 5, 31	H-2, H-6, H-31, H-6 Qui2
31	27.9 CH_3	1.24 s	C: 3, 4, 5, 30	H-3, H-5, H-6, H-30, H-1 Xyl1
32	20.0 CH_3	1.24 s	C: 8, 13, 15	H-7, H-12, H-15, H-17

[a] Recorded at 176.04 MHz in C_5D_5N/D_2O (4/1). [b] Recorded at 700.13 MHz in C_5D_5N/D_2O (4/1).

Table S3. ^{13}C and ^{1}H NMR chemical shifts, HMBC and ROESY correlations of carbohydrate moiety of kuriloside D_1 (**2**).

Atom	δ_C mult. [a, b, c]	δ_H mult. (J in Hz) [d]	HMBC	ROESY
Xyl1 (1→C-3)				
1	105.0 CH	4.70 d (7.6)	C: 3	H-3; H-3, 5 Xyl1
2	**83.3** CH	3.95 t (8.3)	C: 3 Xyl1	H-1 Qui2
3	75.4 CH	4.14 t (8.3)	C: 2 Xyl1	H-1 Xyl1
4	**79.7** CH	4.07 m		H-1 Glc5
5	63.8 CH_2	4.30 dd (6.1; 12.1)		
		3.59 dd (9.1; 12.1)		H-1 Xyl1
Qui2 (1→2Xyl1)				
1	105.3 CH	5.02 d (7.6)	C: 2 Xyl1	H-2 Xyl1; H-3, 5 Qui2
2	75.8 CH	3.95 t (8.3)		H-4 Qui2
3	75.3 CH	4.05 t (8.3)	C: 2, 4 Qui2	H-1 Qui2
4	**87.1** CH	3.56 t (8.3)	C: 1 Glc3; C: 5 Qui2	H-1 Glc3; H-2 Qui2
5	71.6 CH	3.76 dd (6.1; 9.7)		H-1, 3 Qui2
6	17.9 CH_3	1.71 d (6.1)	C: 4, 5 Qui2	
Glc3 (1→4Qui2)				
1	104.8 CH	4.90 d (7.5)	C: 4 Qui2	H-4 Qui2
2	73.6 CH	4.03 t (8.2)	C: 1, 3 Glc3	
3	**88.1** CH	4.20 m	C: 4 Glc3	H-1 Glc4; H-1 Glc3
4	69.7 CH	4.00 m	C: 3, 5 Glc3	
5	78.5 CH	4.00 m		
6	62.1 CH_2	4.47 d (12.3)		
		4.13 m		
Glc4 (1→3Glc3)				
1	105.7 CH	5.28 d (8.2)	C: 3 Glc3	H-3 Glc3; H-3, 5 Glc4
2	75.3 CH	4.06 t (9.1)	C: 1, 3 Glc4	
3	77.9 CH	4.22 t (9.1)	C: 2, 4 Glc4	
4	71.5 CH	4.15 t (9.1)	C: 5, 6 Glc4	
5	78.1 CH	4.00 m		H-1 Glc4
6	62.4 CH_2	4.51 dd (3.0; 11.5)		
		4.28 dd (5.4; 11.5)		
Glc5 (1→4Xyl1)				
1	103.7 CH	4.86 d (7.8)	C: 4 Xyl1	H-4 Xyl1; H-3 Glc5
2	73.2 CH	3.88 t (7.8)	C: 1, 3 Glc5	
3	**87.0** CH	4.14 t (9.2)	C: 1 MeGlc6; C: 2, 4 Glc5	H-1 MeGlc6; H-1 Glc5
4	69.6 CH	3.92 t (9.2)	C: 3, 5, 6 Glc5	
5	76.1 CH	4.11 m		
6	*67.2* CH_2	5.18 d (9.9)		
		4.76 dd (6.6; 11.2)	C: 5 Glc5	
MeGlc6 (1→3Glc4)				
1	105.3 CH	5.24 d (7.9)	C: 3 Glc5	H-3 Glc5; H-3, 5 MeGlc6
2	74.9 CH	3.95 t (8.6)	C: 1, 3 MeGlc6	
3	87.8 CH	3.68 t (8.6)	C: 2, 4 MeGlc6; OMe	H-1, 5 MeGlc6; OMe
4	70.4 CH	4.13 t (8.6)	C: 3, 6 MeGlc6	H-6 MeGlc6
5	78.2 CH	3.92 t (8.6)		H-1, 3 MeGlc6
6	61.9 CH_2	4.44 dd (2.6; 11.8)	C: 4 MeGlc6	
		4.26 dd (5.3; 11.8)	C: 5 MeGlc6	
OMe	60.5 CH_3	3.85 s	C: 3 MeGlc6	

[a] Recorded at 176.04 MHz in C_5D_5N/D_2O (4/1). [b] Bold = interglycosidic positions. [c] Italic = sulfate position. [d] Recorded at 700.13 MHz in C_5D_5N/D_2O (4/1). Multiplicity by 1D TOCSY.

Table S4. ^{13}C and ^{1}H NMR chemical shifts, HMBC and ROESY correlations of the aglycone moiety of kurilosides H (**4**) and I$_1$ (**6**).

Position	δ_C mult.[a]	δ_H mult. (*J* in Hz)[b]	HMBC	ROESY
1	36.2 CH_2	1.77 m		H-11, H-19
		1.38 m		H-3, H-5, H-11
2	27.0 CH_2	2.19 m		H-19
		1.94 m		H-19, H-30
3	88.3 CH	3.18 dd (4.2; 11.9)	C: 1, 30, 31, C-1Xyl1	H-1, H-5, H-31, H-1Xyl1
4	39.7 C			
5	52.7 CH	0.87 brd (12.3)	C: 6, 10, 19, 30	H-1, H-3, H-7, H-31
6	21.1 CH_2	1.70 m		H-30, H-31
		1.50 m		H-19, H-30
7	28.0 CH_2	1.58 m		H-15
		1.27 m		H-5, H-32
8	41.2 CH	2.20 m		H-18
9	148.9 C			
10	39.3 C			
11	114.0 CH	5.23brd (5.6)	C: 8, 10, 12, 14	H-1
12	35.8 CH_2	2.08m		H-17, H-32
		1.78 m	C: 9, 11, 13, 15, 18	H-18, H-21
13	45.5 C			
14	43.6 C			
15	43.7 CH_2	2.10dd (6.3; 13.6)	C: 32	H-32
		1.35dd (5.0; 13.6)	C: 8, 13, 16, 32	H-18
16	73.8 CH	5.64 dd (5.2; 7.7; 13.4)	C: 15; OAc-16	H-32; OAc-16
17	53.3 CH	2.41dd (7.7; 10.6)	C: 12, 15, 18, 20, 21	H-12, H-21, H-32
18	15.2 CH_3	0.82 s	C: 12, 13, 14, 17	H-8, H-12, H-19, H-20, H-21
19	22.3 CH_3	1.14 s	C: 1, 5, 9, 10	H-1, H-2, H-6, H-8, H-18, H-30
20	69.4 CH	5.46 dd (6.1; 10.6)	C: 16, 17, 21, OAc-20	H-21, OAc-20
21	19.6 CH_3	1.32d (6.0)	C: 17, 20	H-12, H-17, H-18, H-20
30	16.4 CH_3	1.04 s	C: 3, 4, 5, 31	H-2, H-6, H-31
31	27.8 CH_3	1.24 s	C: 3, 4, 5, 30	H-3, H-5, H-6, H-30, H-1 Xyl1
32	18.9 CH_3	0.76 s	C: 8, 13, 14, 15	H-7, H-12, H-15, H-16, H-17
<u>C</u>OOCH$_3$-16	169.8 C			
COO<u>C</u>H$_3$-16	20.2 CH_3	2.12 s		H-16, H-18
<u>C</u>OOCH$_3$-20	169.9 C			
COO<u>C</u>H$_3$-20	21.0 CH_3	2.05 s		H-21

[a]Recorded at 176.03 MHz in C_5D_5N/D_2O (4/1). [b]Recorded at 700.00 MHz in C_5D_5N/D_2O (4/1).

Table S5. ^{13}C and ^{1}H NMR chemical shifts, HMBC and ROESY correlations of carbohydrate moiety of DS-kuriloside L (**10**).

Atom	δ_C mult. [a, b]	δ_H mult. (*J* in Hz) [c]	HMBC	ROESY
Xyl1 (1→C-3)				
1	105.2 CH	4.75 d (7.6)	C: 3; C: 5 Xyl1	H-3; H-3, 5 Xyl1
2	**83.2** CH	4.10 dd (7.6; 8.7)	C: 1 Qui2; C: 1, 3 Xyl1	H-1 Qui2
3	75.7 CH	4.23 t (8.7)	C: 2, 4 Xyl1	H-1, 5 Xyl1
4	**77.5** CH	4.29 m	C: 1 Glc3; C: 3 Xyl1	H-1 Glc3
5	63.9 CH_2	4.39 dd (4.6; 11.6)	C: 1, 3, 4 Xyl1	H-1 Xyl1
		3.63 dd (8.7; 11.6)	C: 4 Xyl1	H-1, 3 Xyl1
Qui2 (1→2Xyl1)				
1	106.0 CH	5.20 d (8.0)	C: 2 Xyl1	H-2 Xyl1; H-3, 5 Qui2
2	77.0 CH	4.06 t (8.0)	C: 1, 3 Qui2	H-4 Qui2
3	77.6 CH	4.14 t (8.6)	C: 4 Qui2	H-1, 5 Qui2
4	76.5 CH	3.73 t (9.2)	C: 3, 5 Qui2	H-2 Qui2
5	73.3 CH	3.82 dd (6.3; 9.2)	C: 3, 4, 6 Qui2	H-1, 3 Qui2
6	18.4 CH_3	1.70 d (6.3)	C: 4, 5 Qui2	H-4, 5 Qui2
Glc3 (1→4Xyl1)				
1	103.3 CH	5.02 d (8.4)	C: 4 Xyl1	H-4 Xyl1; H-3, 5 Glc3
2	74.2 CH	4.00 t (9.0)	C: 3 Glc3	
3	78.0 CH	4.20 t (9.0)	C: 4 Glc3	H-1 Glc3
4	71.5 CH	4.19 t (9.0)	C: 3 Glc3	
5	78.5 CH	3.98 m		H-1, 3 Glc3
6	62.4 CH_2	4.54 brd (10.3)		
		4.32 dd (5.2; 10.3)	C: 5 Glc3	

[a] Recorded at 176.04 MHz in C_5D_5N. [b] Bold = interglycosidic positions. [c] Recorded at 700.13 MHz in C_5D_5N. Multiplicity by 1D TOCSY.

Table S6. ^{13}C and ^{1}H NMR chemical shifts, HMBC and ROESY correlations of the aglycone moiety of DS-kuriloside L (**10**).

Position	δ_C mult.[a]	δ_H mult. (*J* in Hz)[b]	HMBC	ROESY
1	36.2 CH_2	1.75 m		H-5, H-11
		1.41 m		H-11
2	26.9 CH_2	2.20 m		
		1.96 m		H-19, H-30
3	88.5 CH	3.23 dd (4.2; 11.7)	C: 4, 30, 31, C-1Xyl1	H-1, H-5, H-31, H-1Xyl1
4	39.8 C			
5	52.9 CH	0.94 brdd (1.9; 11.7)	C: 4, 19, 30, 31	H-1, H-3, H-31
6	21.0 CH_2	1.74 m		
		1.53 m		H-8, H-19, H-30
7	27.9 CH_2	1.67 m		
		1.35 m		H-5, H-32
8	39.4 CH	2.39 brd (12.2)	C: 7	
9	149.1 C			
10	39.4 C			
11	115.2 CH	5.35 brd (5.6)	C: 8, 13	H-1
12	32.1 CH_2	2.61 brdd (5.6; 17.8)	C: 9, 11, 13, 14	H-18
		2.49 brd (17.8)		H-32
13	49.6 C			
14	47.0 C			
15	41.7 CH_2	2.26 brd (16.8)	C: 16, 17, 32	H-18
		2.06 brdd (3.7; 16.8)	C: 13, 14, 16, 17, 32	H-7, H-32
16	144.3 CH	6.63 brt (2.6)		
17	152.1 C			
18	19.3 CH_3	1.00 s	C: 12, 13, 14, 17	H-8, H-12, H-15
19	22.1 CH_3	1.12 s	C: 1, 5, 8, 9	H-2, H-6, H-8
20	196.1 C			
21	26.7 CH_3	2.29 s	C: 20	
30	16.5 CH_3	1.17 s	C: 3, 4, 5, 31	H-2, H-6, H-31
31	28.0 CH_3	1.32 s	C: 3, 4, 5, 30	H-3, H-6, H-30, H-1 Xyl1
32	19.8 CH_3	0.88 s	C: 8, 13, 14, 15	H-7, H-12, H-15

[a]Recorded at 176.03 MHz in C_5D_5N. [b]Recorded at 700.00 MHz in C_5D_5N.

Table S7. ^{13}C and ^{1}H NMR chemical shifts, HMBC and ROESY correlations of carbohydrate moiety of DS-kuriloside M (**11**).

Atom	δ_C mult. [a, b]	δ_H mult. (*J* in Hz) [c]	HMBC	ROESY
Xyl1 (1→C-3)				
1	105.1 CH	4.75 d (6.4)	C: 3	H-3; H-3, 5 Xyl1
2	**83.5** CH	4.03 t (8.9)	C: 1, 3 Xyl1	H-1 Qui2
3	75.6 CH	4.22 t (8.9)	C: 2, 4 Xyl1	H-1, 5 Xyl1
4	**77.2** CH	4.26 dd (5.1; 8.5)	C: 1 Glc4; C: 3 Xyl1	H-1 Glc4
5	63.9 CH_2	4.39 dd (5.0; 11.2)	C: 1, 3 Xyl1	
		3.64 dd (9.1; 11.2)		H-1 Xyl1
Qui2 (1→2Xyl1)				
1	105.4 CH	5.15 d (8.1)	C: 2 Xyl1	H-2 Xyl1; H-3, 5 Qui2
2	76.2 CH	4.04 t (9.1)	C: 1, 3 Qui2	H-4 Qui2
3	75.8 CH	4.15 t (9.1)	C: 2, 4 Qui2	H-1, 5 Qui2
4	**87.2** CH	3.68 t (9.1)	C: 1 Glc3; C: 3, 5 Qui2	H-1 Glc3; H-2 Qui2
5	71.5 CH	3.82 dd (6.1; 9.1)		H-1, 3 Qui2
6	18.1 CH_3	1.77 d (6.1)	C: 4, 5 Qui2	
Glc3 (1→4Qui2)				
1	105.3 CH	5.01 d (7.5)	C: 4 Qui2	H-4 Qui2; H-3, 5 Glc3
2	74.8 CH	4.05 t (8.5)	C: 1 Glc3	
3	78.1 CH	4.23 t (8.5)	C: 2, 4 Glc3	H-1 Glc3
4	71.6 CH	4.15 t (8.5)	C: 5 Glc3	H-6 Glc3
5	78.2 CH	4.08 t (8.5)		H-1, 3 Glc3
6	62.5 CH_2	4.60 brd (13.2)		
		4.29 m		
Glc4 (1→4Xyl1)				
1	102.7 CH	4.98 d (7.5)	C: 4 Xyl1	H-4 Xyl1; H-3, 5 Glc4
2	72.9 CH	4.00 t (8.5)	C: 1 Glc4	
3	**88.0** CH	4.18 t (8.5)	C: 1 MeGlc5; C: 2 Glc4	H-1 MeGlc5; H-1 Glc4
4	69.6 CH	4.07 t (8.5)	C: 5, 6 Glc4	
5	78.3 CH	3.89 m		H-1, 3 Glc4
6	62.0 CH_2	4.44 brd (13.2)		
		4.22 brdd (5.6; 13.2)		
MeGlc5 (1→3Glc4)				
1	105.5 CH	5.24 d (8.5)	C: 3 Glc4	H-3 Glc4; H-3, 5 MeGlc5
2	75.0 CH	3.99 t (8.5)	C: 1, 3 MeGlc5	
3	87.8 CH	3.71 t (8.5)	C: 2, 4 MeGlc5; OMe	
4	70.4 CH	4.14 t (8.5)	C: 5, 6 MeGlc5	
5	78.2 CH	3.95 m		H-1, 3 MeGlc5
6	62.1 CH_2	4.46 brd (12.2)		
		4.27 m		
OMe	60.5 CH_3	3.86 s	C: 3 MeGlc5	

[a] Recorded at 176.04 MHz in C_5D_5N. [b] Bold = interglycosidic positions. [c] Recorded at 700.13 MHz in C_5D_5N. Multiplicity by 1D TOCSY.

Table S8. ^{13}C and ^{1}H NMR chemical shifts, HMBC and ROESY correlations of the aglycone moiety of DS-kuriloside M (**11**).

Position	δ_C mult.[a]	δ_H mult. (*J* in Hz)[b]	HMBC	ROESY
1	35.7 CH_2	1.45 m	C: 2, 3, 10, 19	H-3, H-5, H-11, H-19
2	27.2 CH_2	2.16 m		
		1.94 m		H-19, H-30
3	88.7 CH	3.29 dd (4.1; 11.6)	C: 4, 30, 31, C-1Xyl1	H-1, H-5, H-31, H-1Xyl1
4	39.5 C			
5	49.2 CH	1.00 brdd (3.5; 12.3)		H-1, H-3, H-31
6	23.1 CH_2	2.05 m	C: 7, 8, 10	H-31
		1.99 m		H-19, H-30
7	122.8 CH	5.72 m	C: 9	
8	147.6 C			
9	47.7 CH	2.31 m		H-19
10	35.5 C			
11	22.4 CH_2	1.80 m		H-1
		1.61 m		
12	33.3 CH_2	2.18 m	C: 9, 14, 16, 18	H-17, H-32
		1.74 m	C: 13, 18	
13	52.4 C			
14	45.6 C			
15	45.0 CH_2	2.31 m	C: 13, 32	H-16, H-18
		2.06 d (12.9)	C: 13, 14, 16, 32	H-32
16	71.5 CH	5.35 m		H-18
17	71.8 CH	3.22 d (6.2)	C: 12, 14, 16, 18, 20	H-21, H-32
18	25.6 CH_3	1.02 s	C: 12, 13, 14, 17	H-9
19	24.4 CH_3	1.07 s	C: 1, 5, 9, 10	H-1, H-2, H-9, H-30
20	208.6 C			
21	30.78 CH_3	2.20 s	C: 17, 20	H-12, H-17
30	17.2 CH_3	1.17 s	C: 3, 4, 5, 31	H-2, H-6, H-19, H-31
31	28.6 CH_3	1.32 s	C: 3, 4, 5, 30	H-3, H-5, H-6, H-30, H-1 Xyl1
32	31.4 CH_3	1.59 s	C: 8, 13, 15	H-11, H-12, H-15, H-17

[a]Recorded at 176.03 MHz in C_5D_5N. [b]Recorded at 700.00 MHz in C_5D_5N.

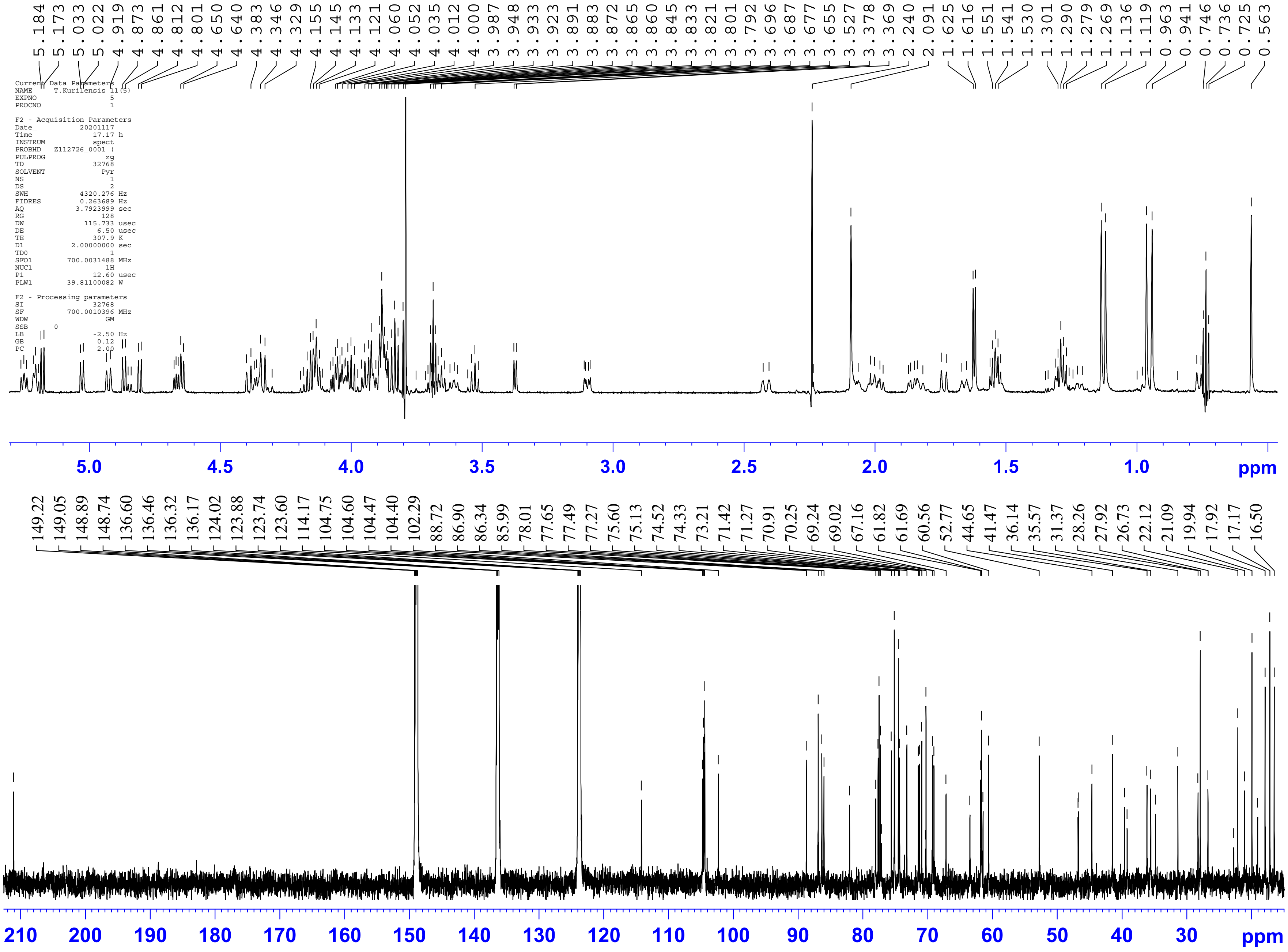

Figure S1. The ^{1}H NMR (700.00 MHz) and ^{13}C NMR (176.03 MHz) spectra of kuriloside A_3 (**1**) in C_5D_5N/D_2O (4/1)

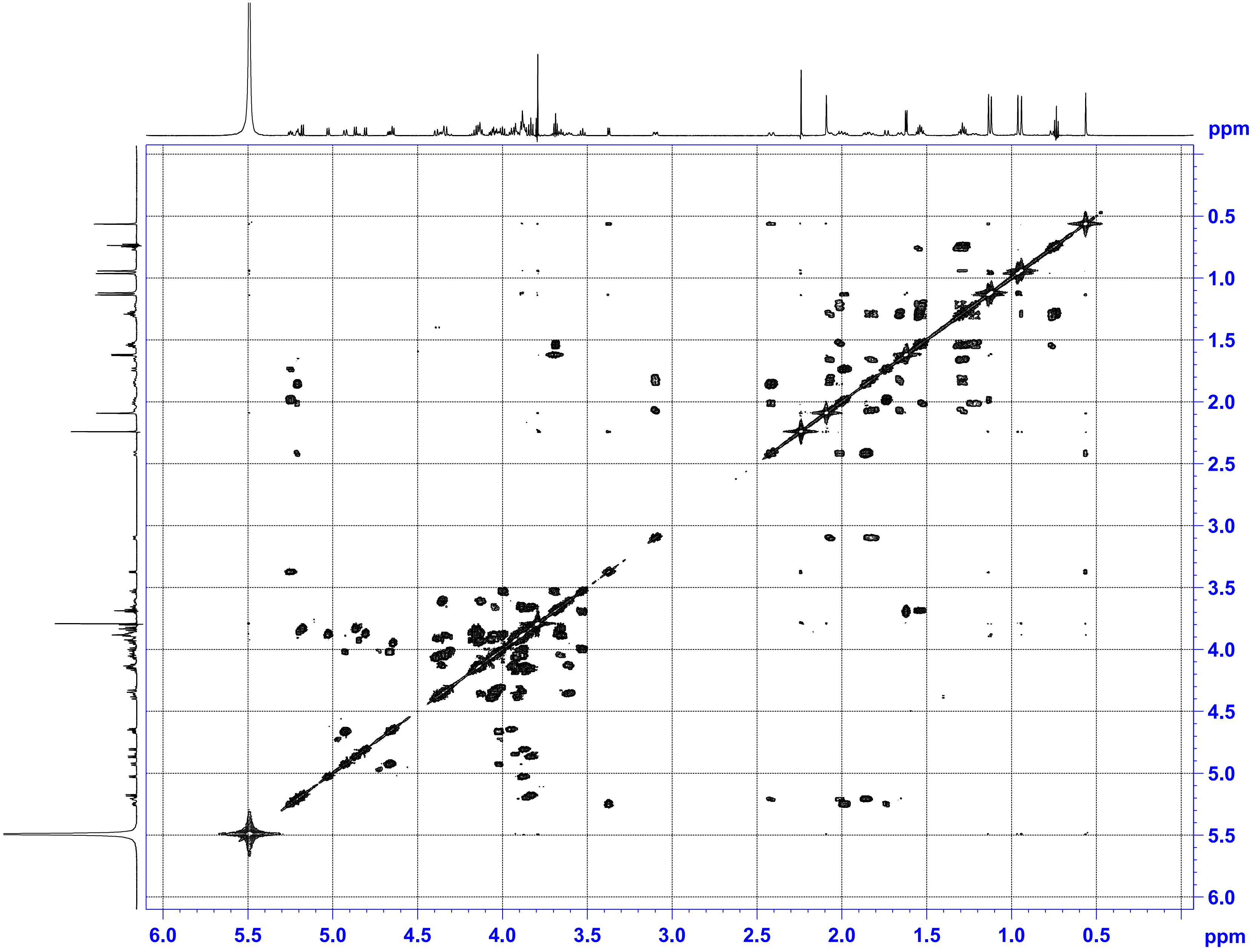

Figure S2. The COSY (700.00 MHz) spectrum of kuriloside A3 (**1**) in C_5D_5N/D_2O (4/1)

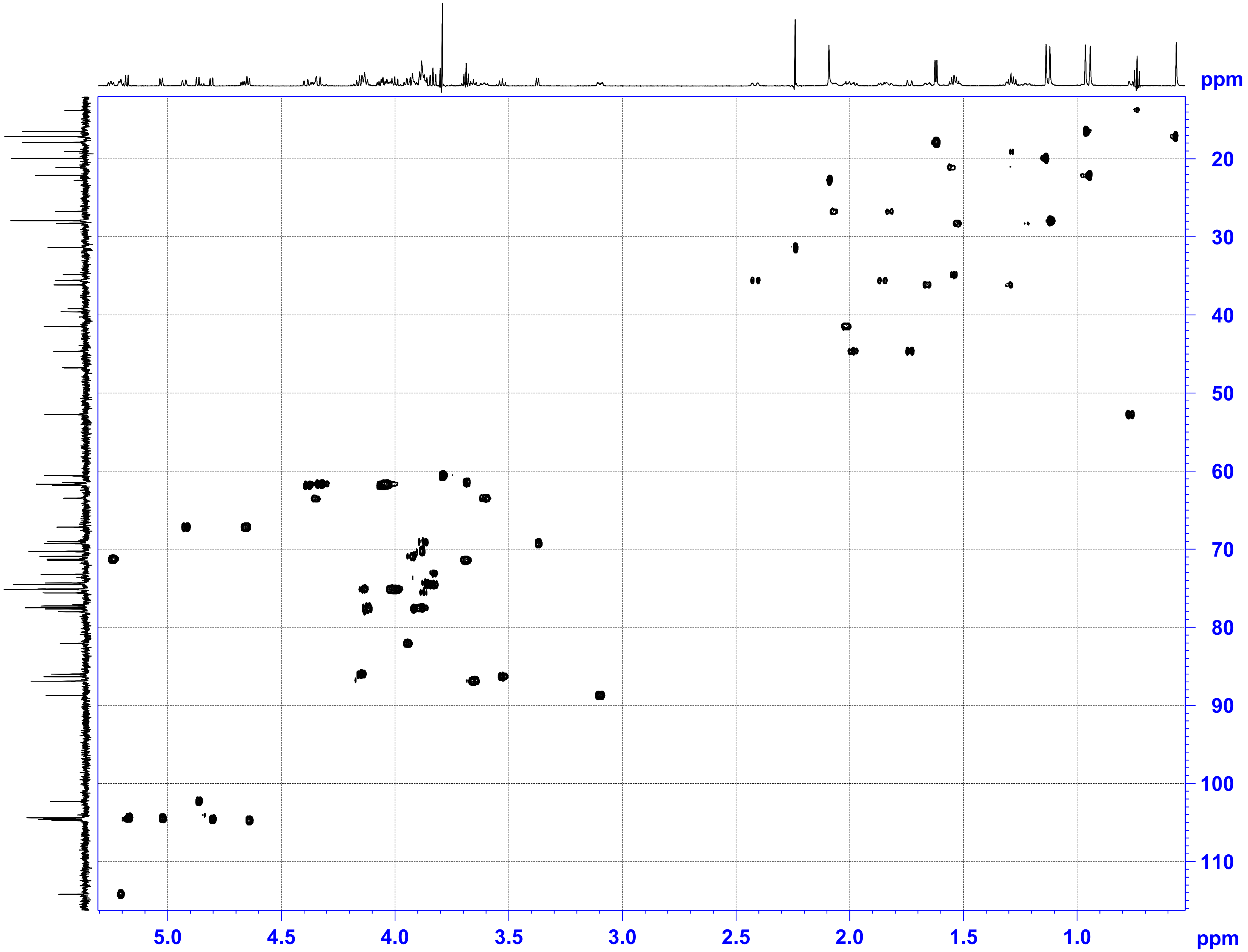

Figure S3. The HSQC (700.00 MHz) spectrum of kuriloside A3 (**1**) in C_5D_5N/D_2O (4/1)

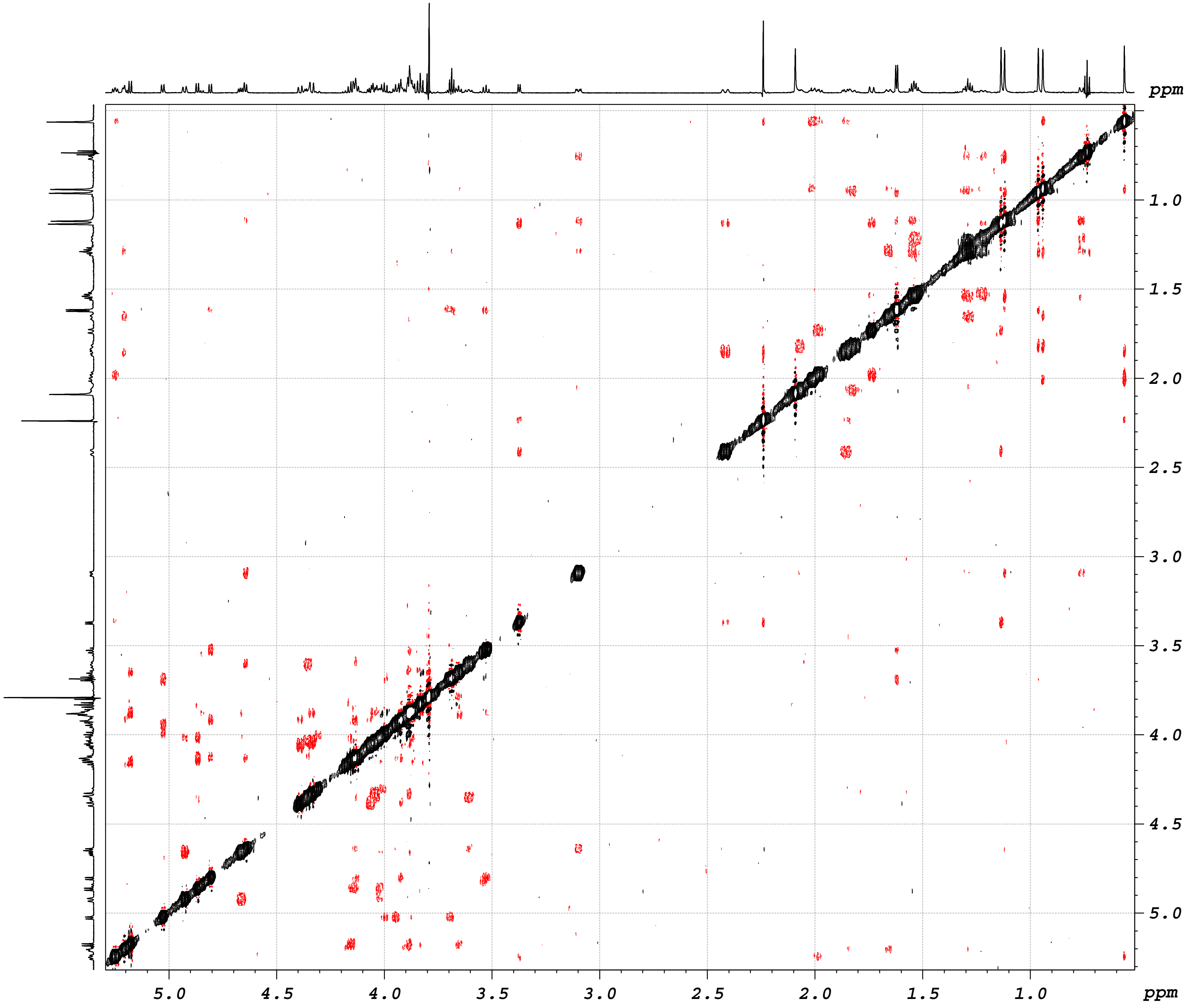

Figure S4. The ROESY (700.00 MHz) spectrum of kuriloside A3 (**1**) in C_5D_5N/D_2O (4/1)

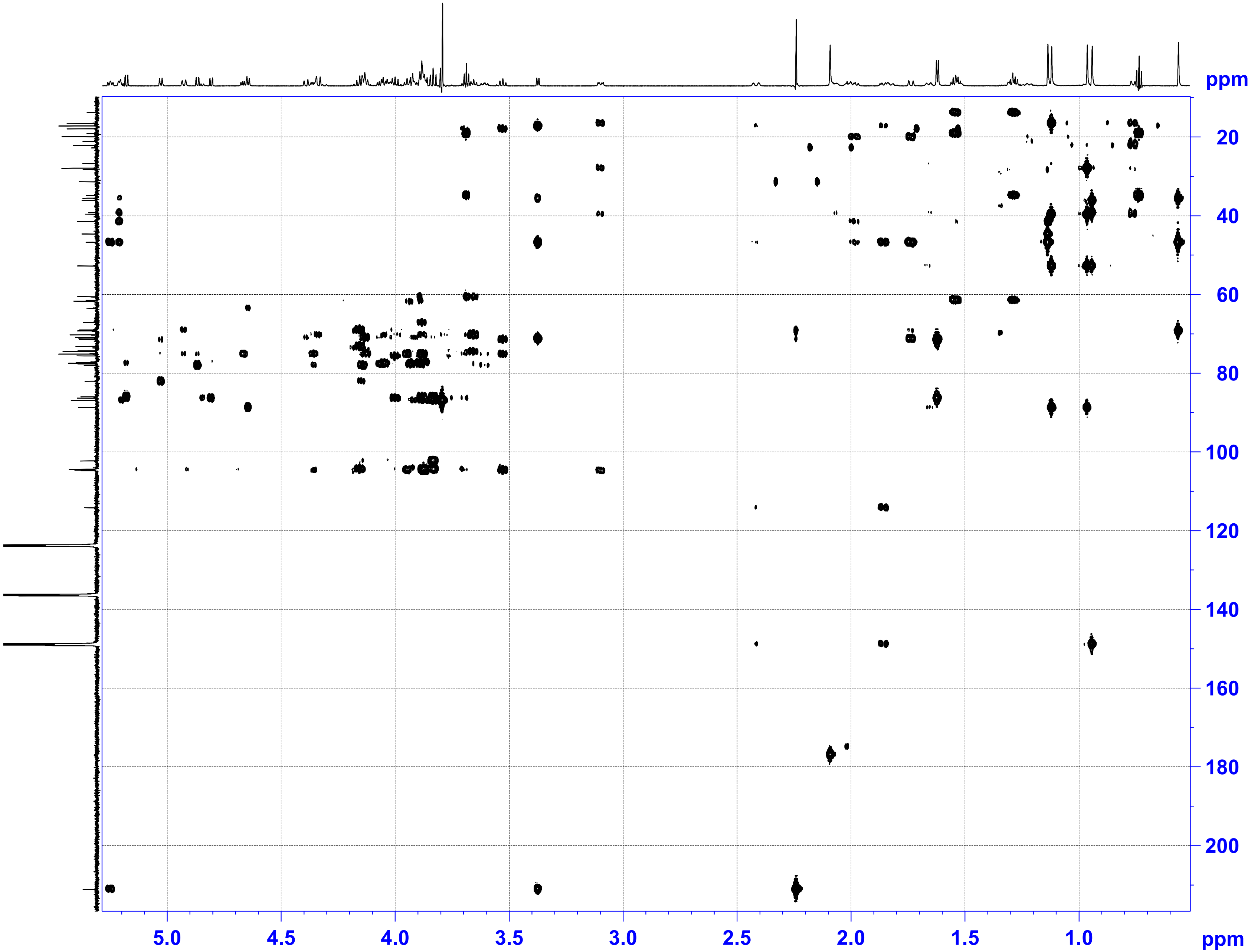

Figure S5. The HMBC (700.00 MHz) spectrum of kuriloside A3 (**1**) in C_5D_5N/D_2O (4/1)

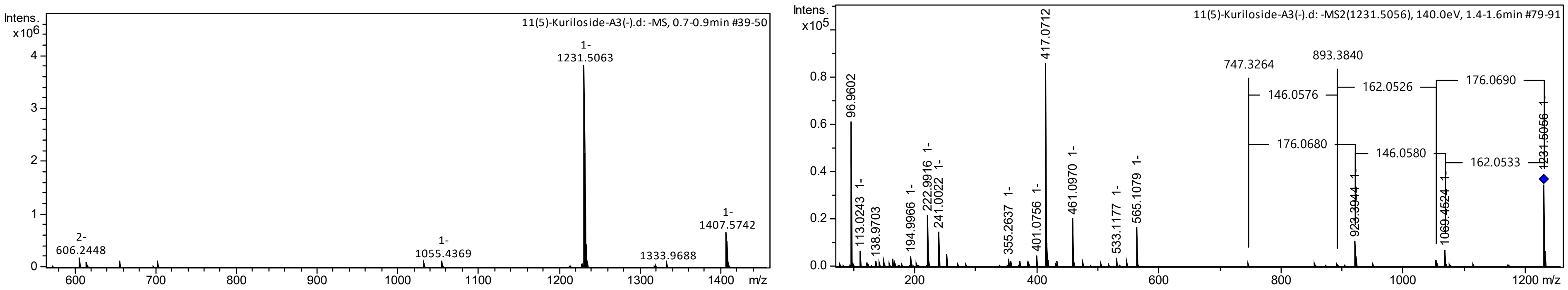

Figure S6. HR-ESI-MS and ESI-MS/MS spectra of kuriloside A3 (**1**)

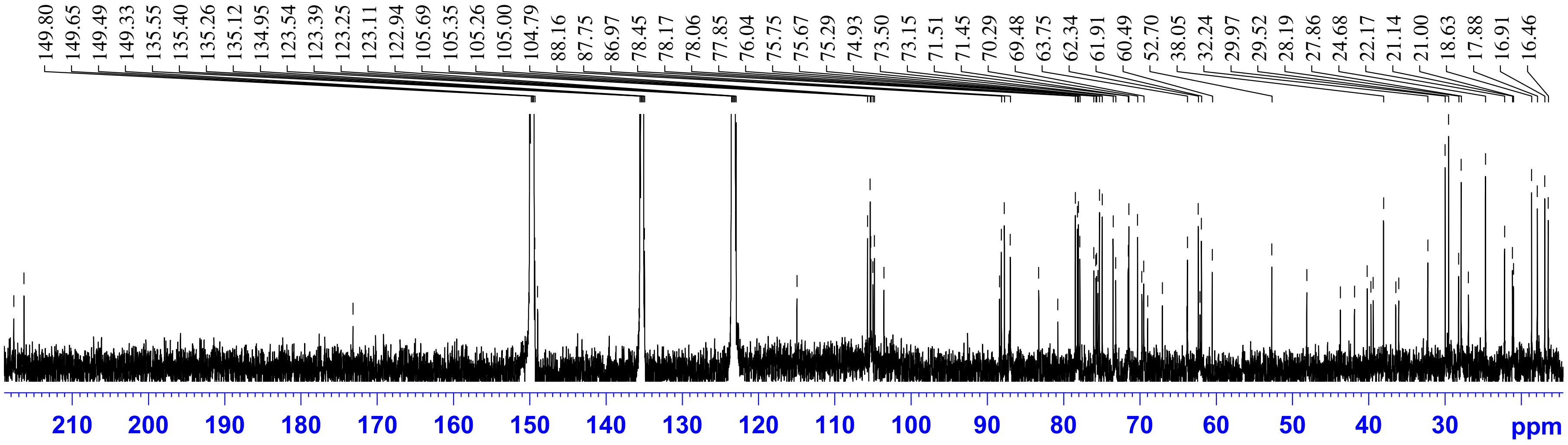

Figure S7. The ^{13}C NMR (176.03 MHz) spectrum of kuriloside D1 (**2**) in C_5D_5N/D_2O (4/1)

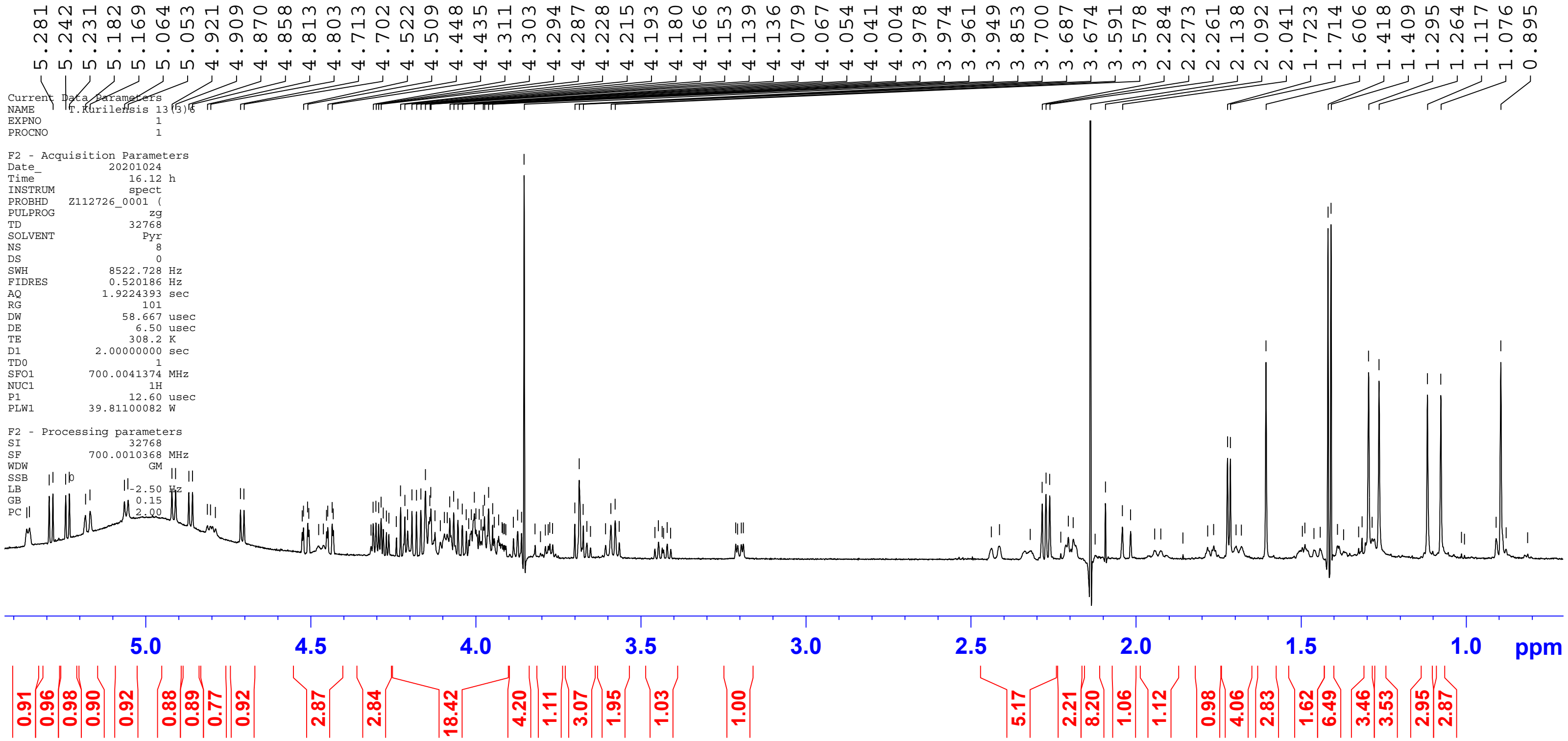

Figure S8. The ^{1}H NMR (700.00 MHz) spectrum of kuriloside D_1 (**2**) in C_5D_5N/D_2O (4/1)

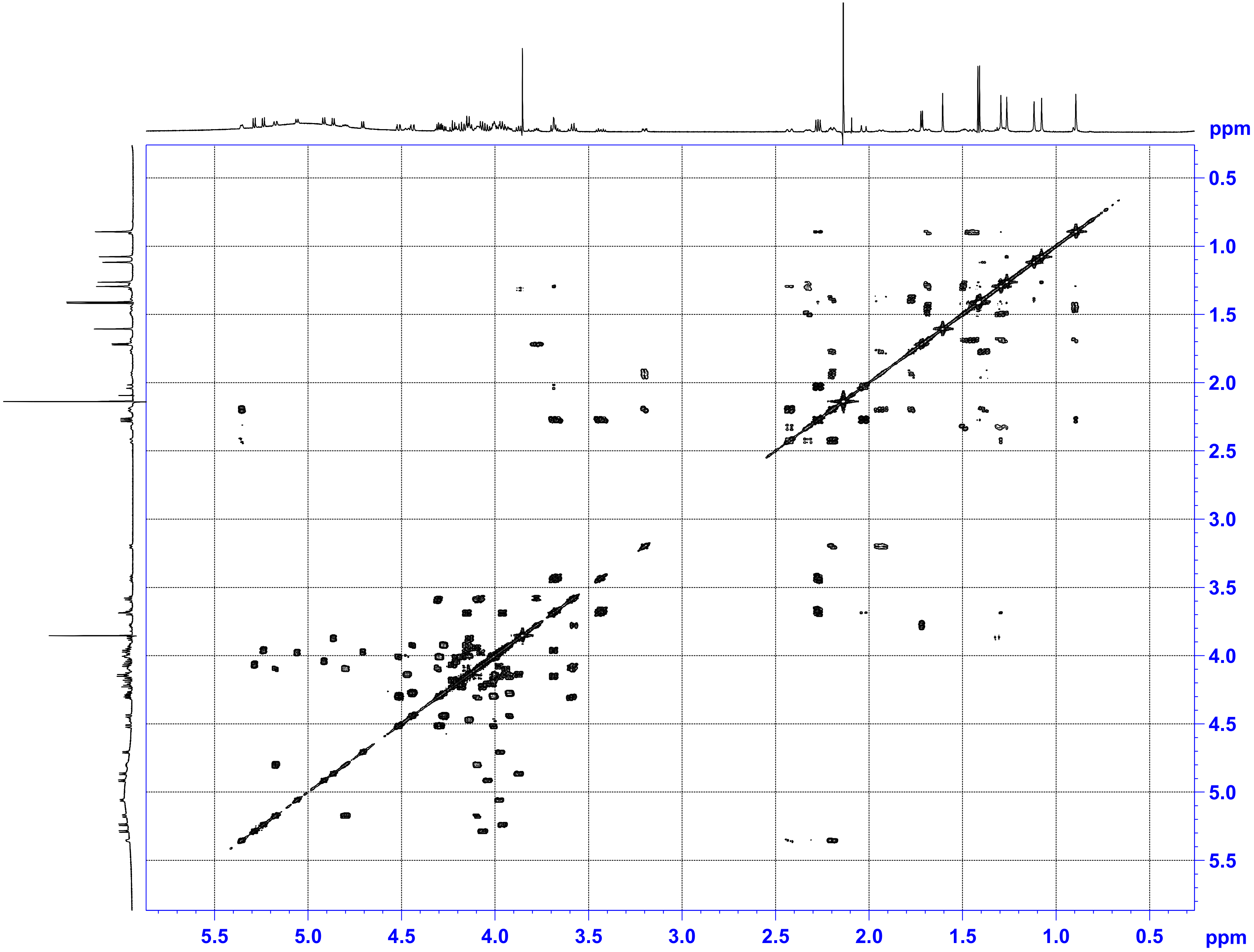

Figure S9. The COSY (700.00 MHz) spectrum of kuriloside D1 (**2**) in C_5D_5N/D_2O (4/1)

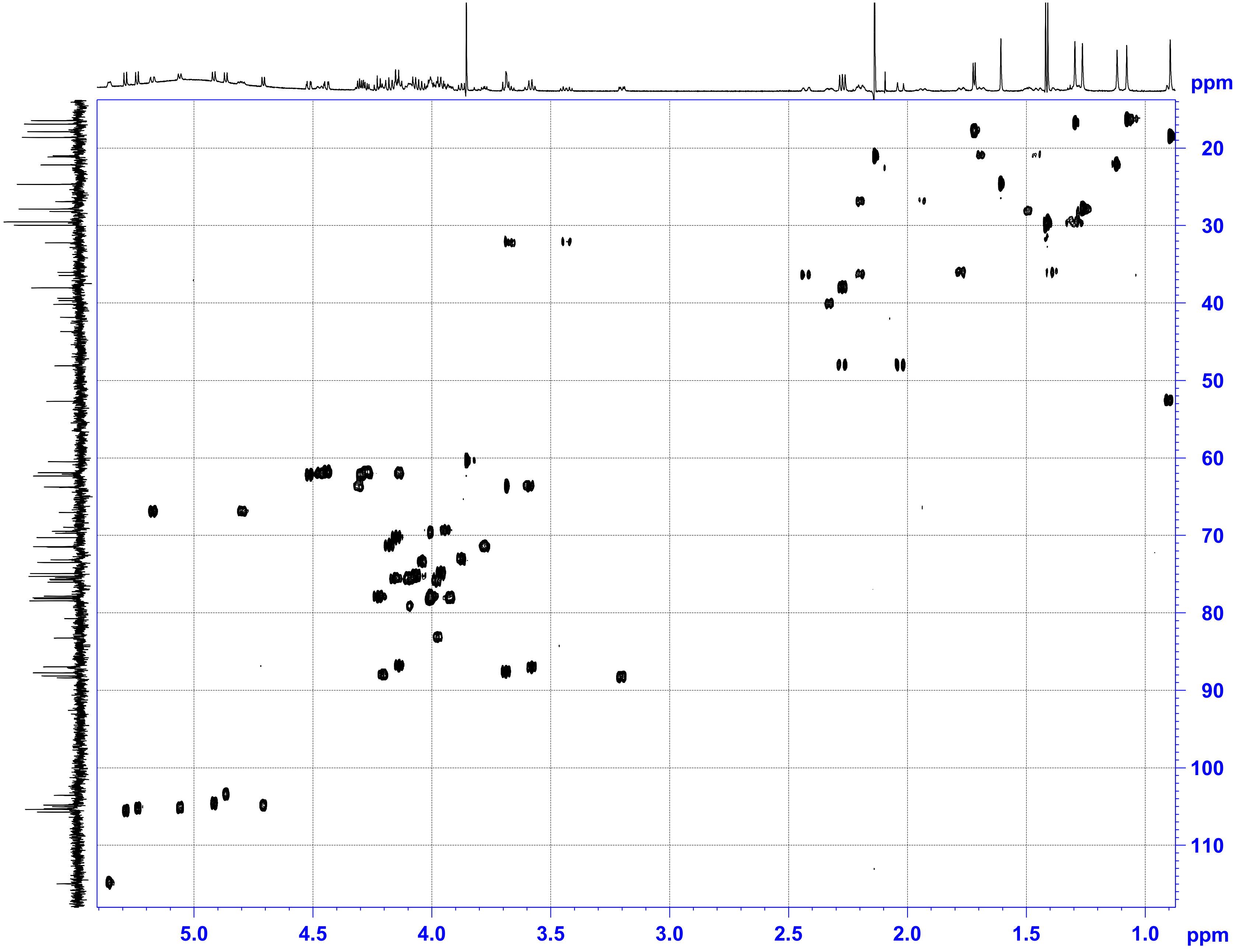

Figure S10. The HSQC (700.00 MHz) spectrum of kuriloside D_1 (**2**) in C_5D_5N/D_2O (4/1)

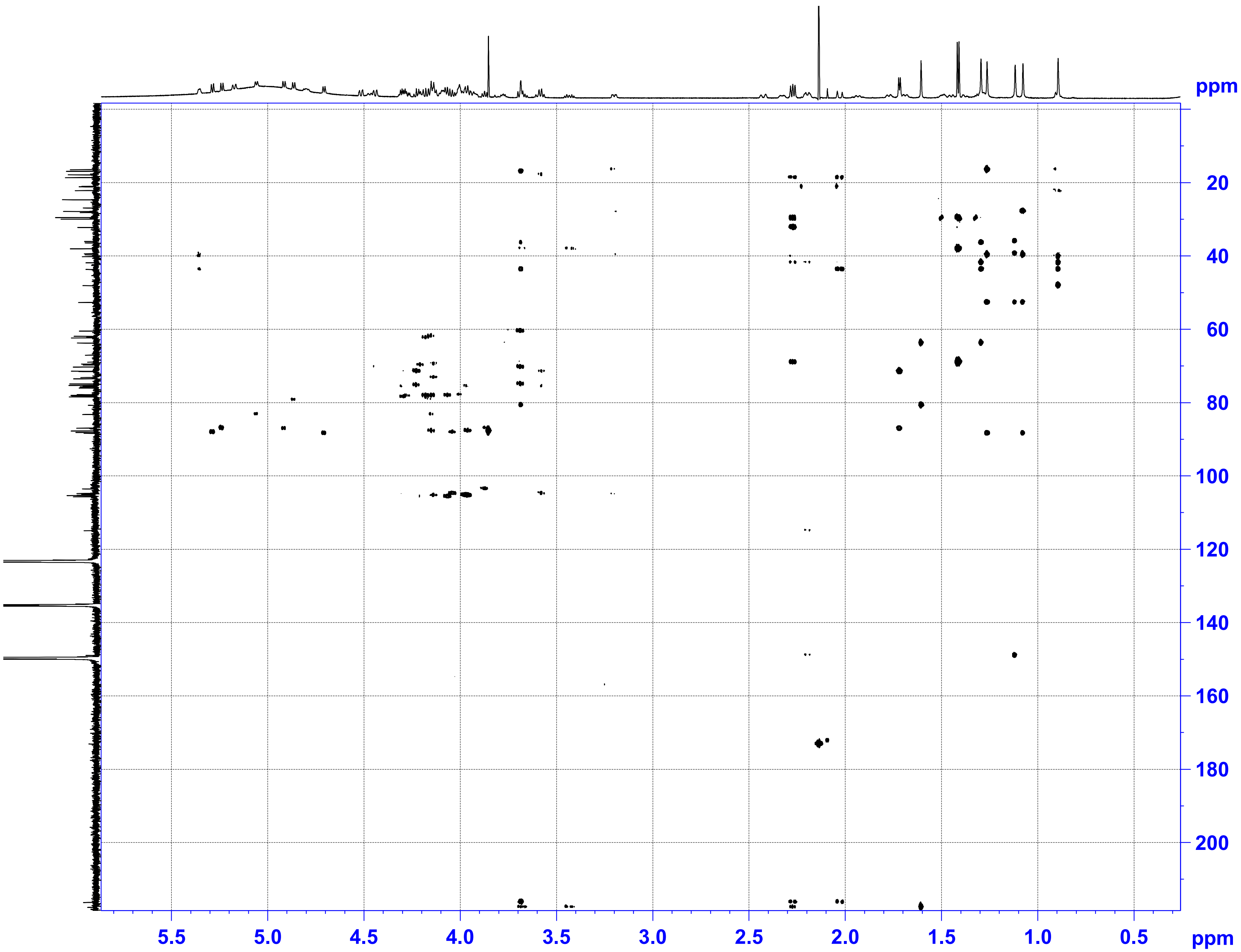

Figure S11. The HMBC (700.00 MHz) spectrum of kuriloside D_1 (**2**) in C_5D_5N/D_2O (4/1)

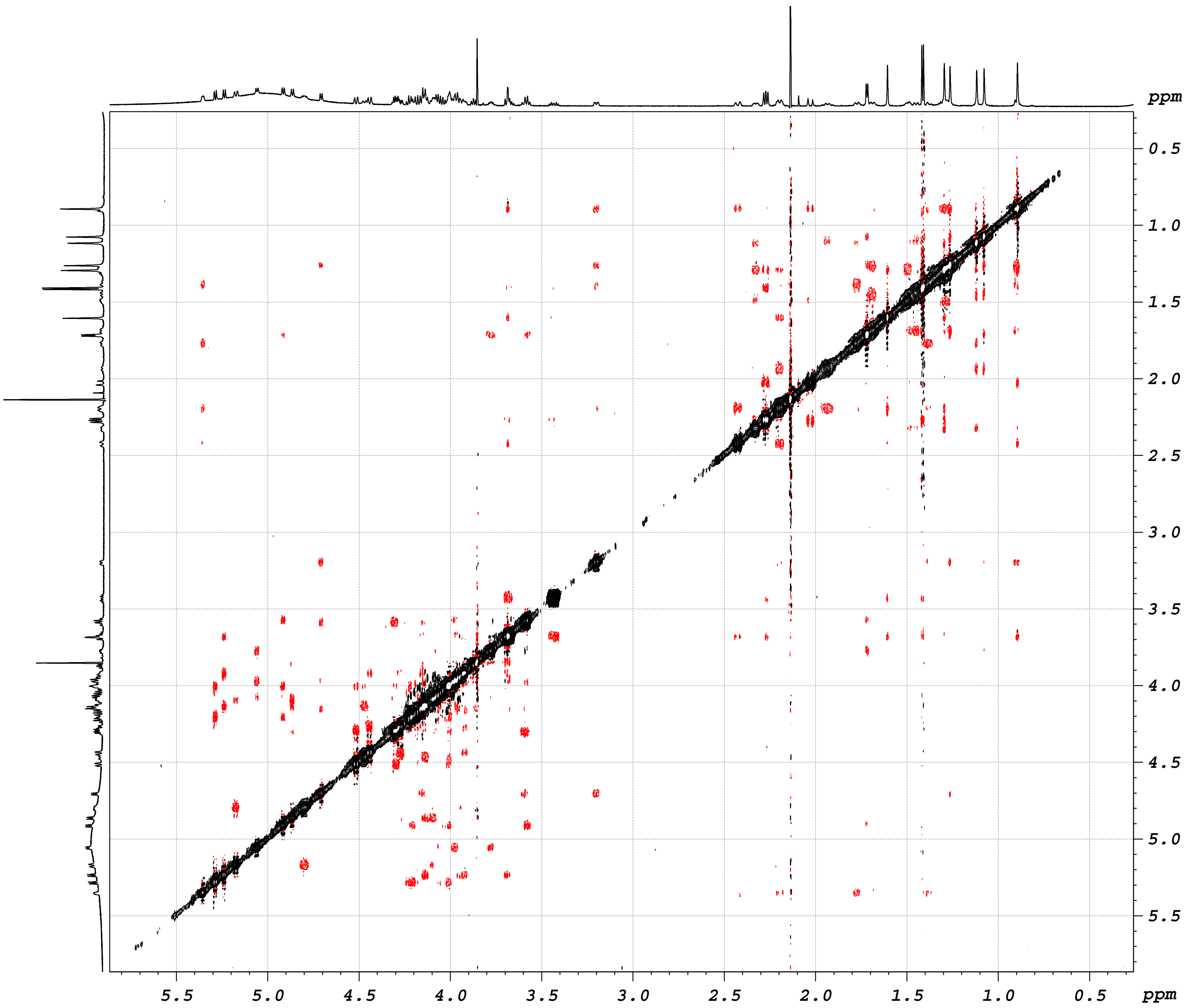

Figure S12. The ROESY (700.00 MHz) spectrum of kuriloside D_1 (**2**) in C_5D_5N/D_2O (4/1)

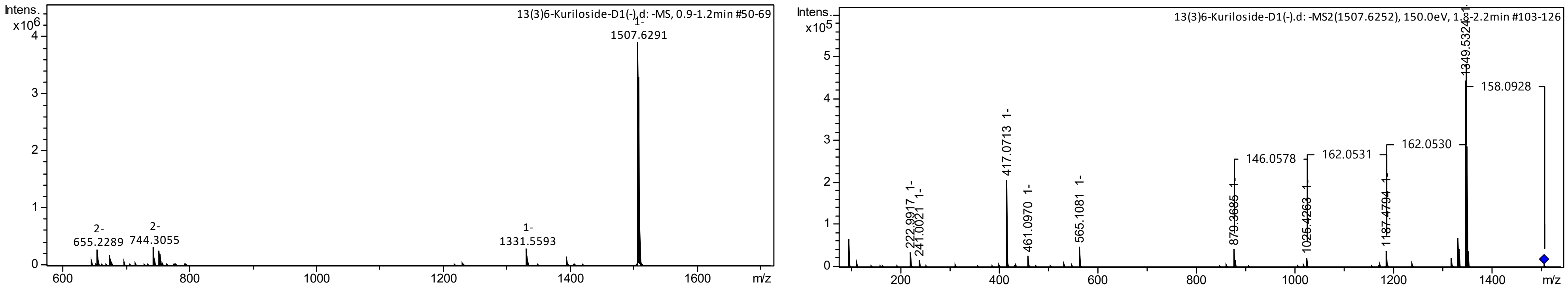

Figure S13. HR-ESI-MS and ESI-MS/MS spectra of kuriloside D1 (**2**)

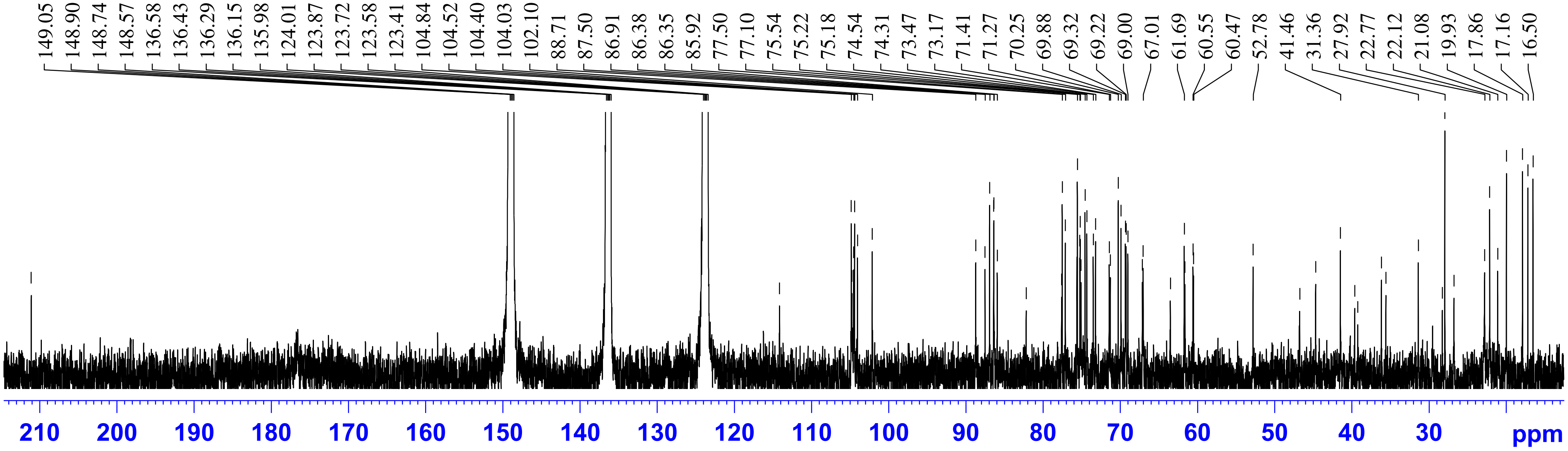

Figure S14. The ^{13}C NMR (176.03 MHz) spectrum of kuriloside G (**3**) in C_5D_5N/D_2O (4/1)

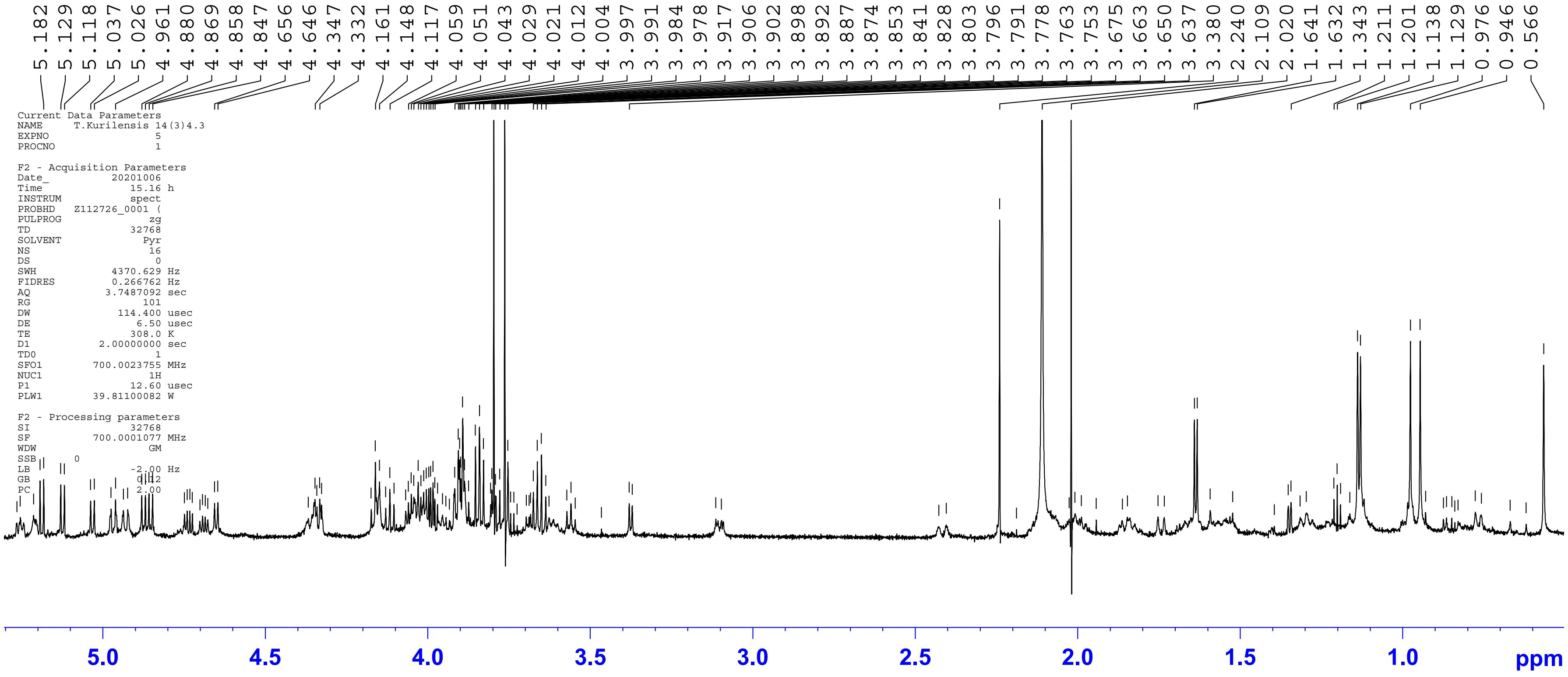

Figure S15. The ^{1}H NMR (700.00 MHz) spectrum of kuriloside G (**3**) in C_5D_5N/D_2O (4/1)

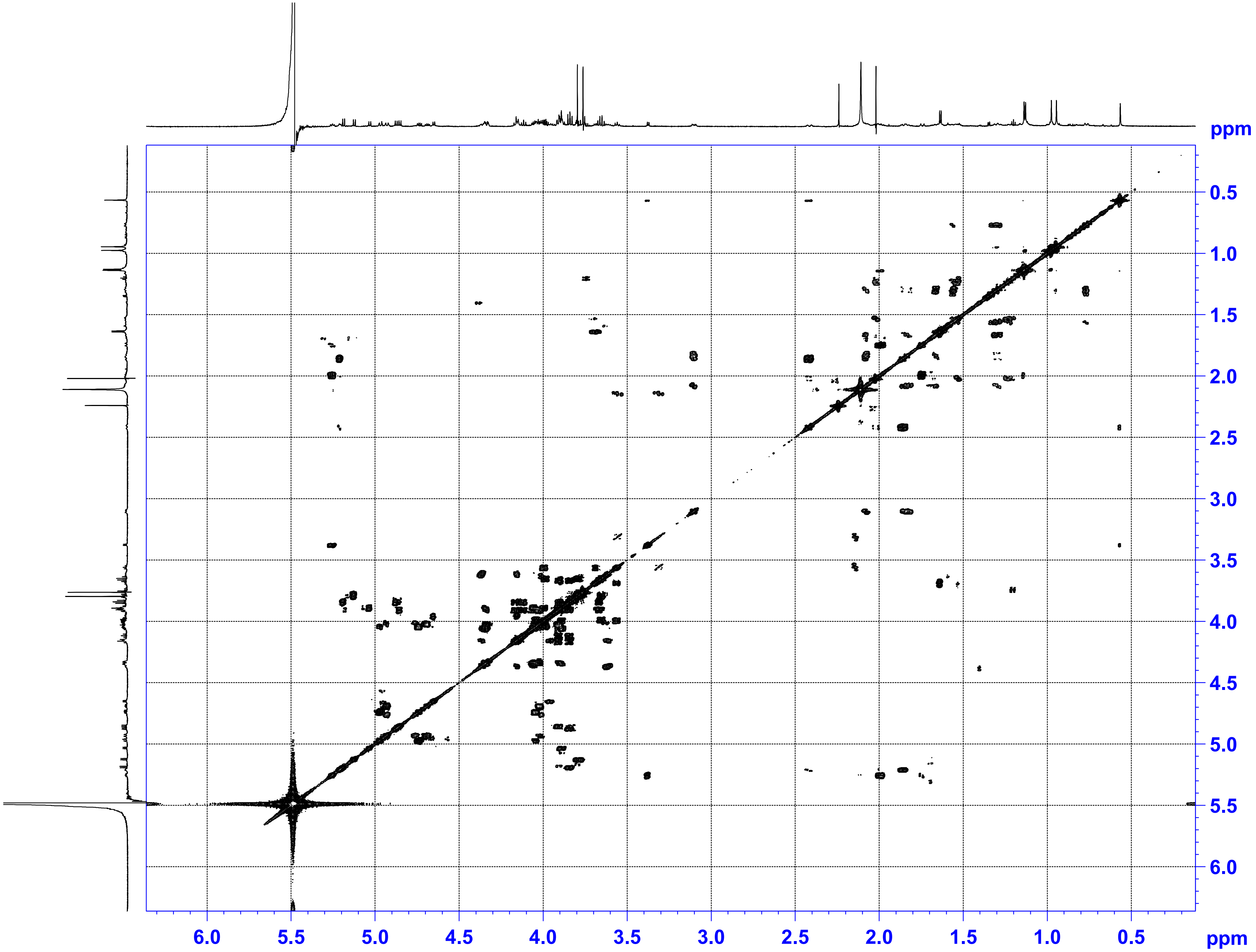

Figure S16. The COSY (700.00 MHz) spectrum of kuriloside G (**3**) in C_5D_5N/D_2O (4/1)

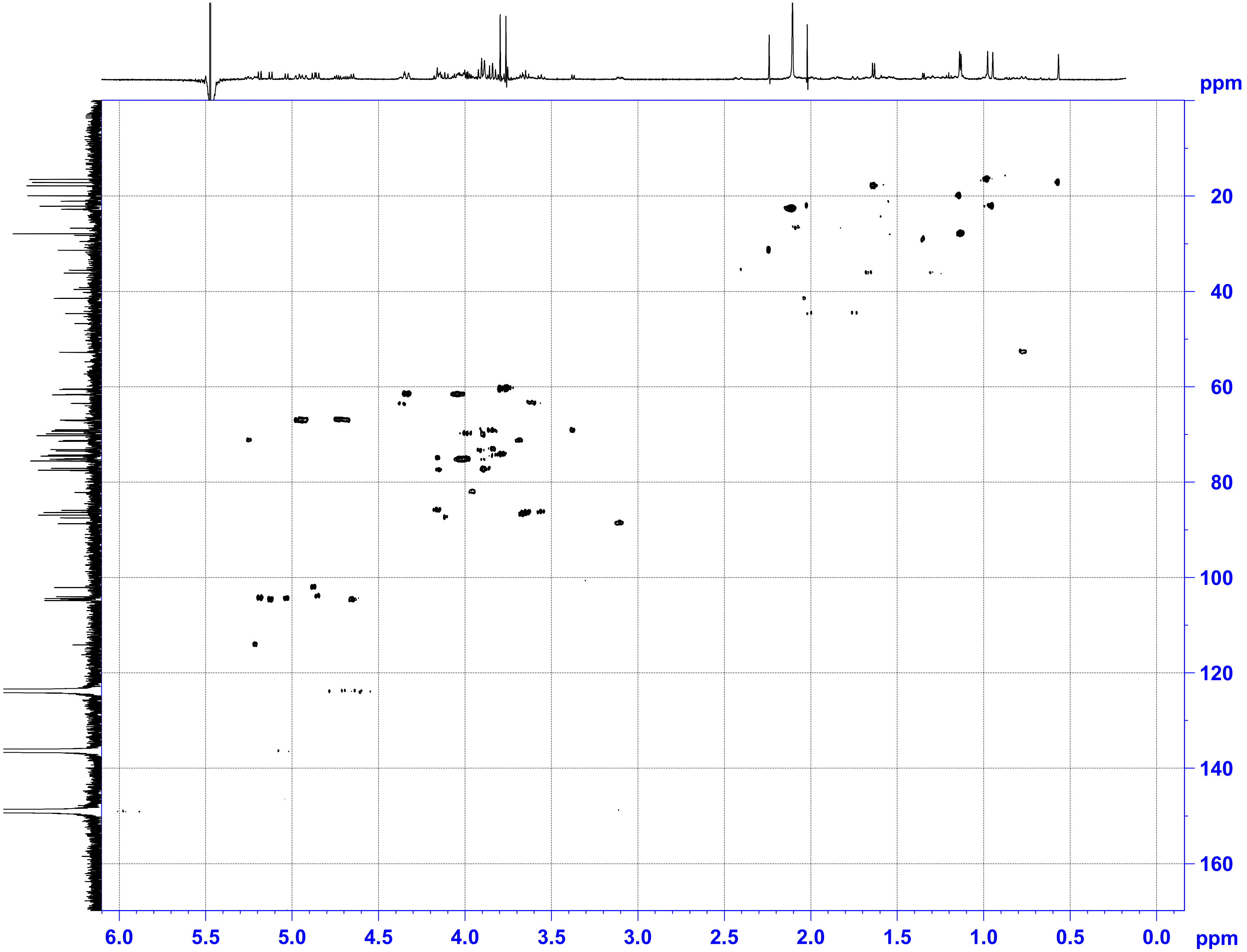

Figure S17. The HSQC (700.00 MHz) spectrum of kuriloside G (**3**) in C_5D_5N/D_2O (4/1)

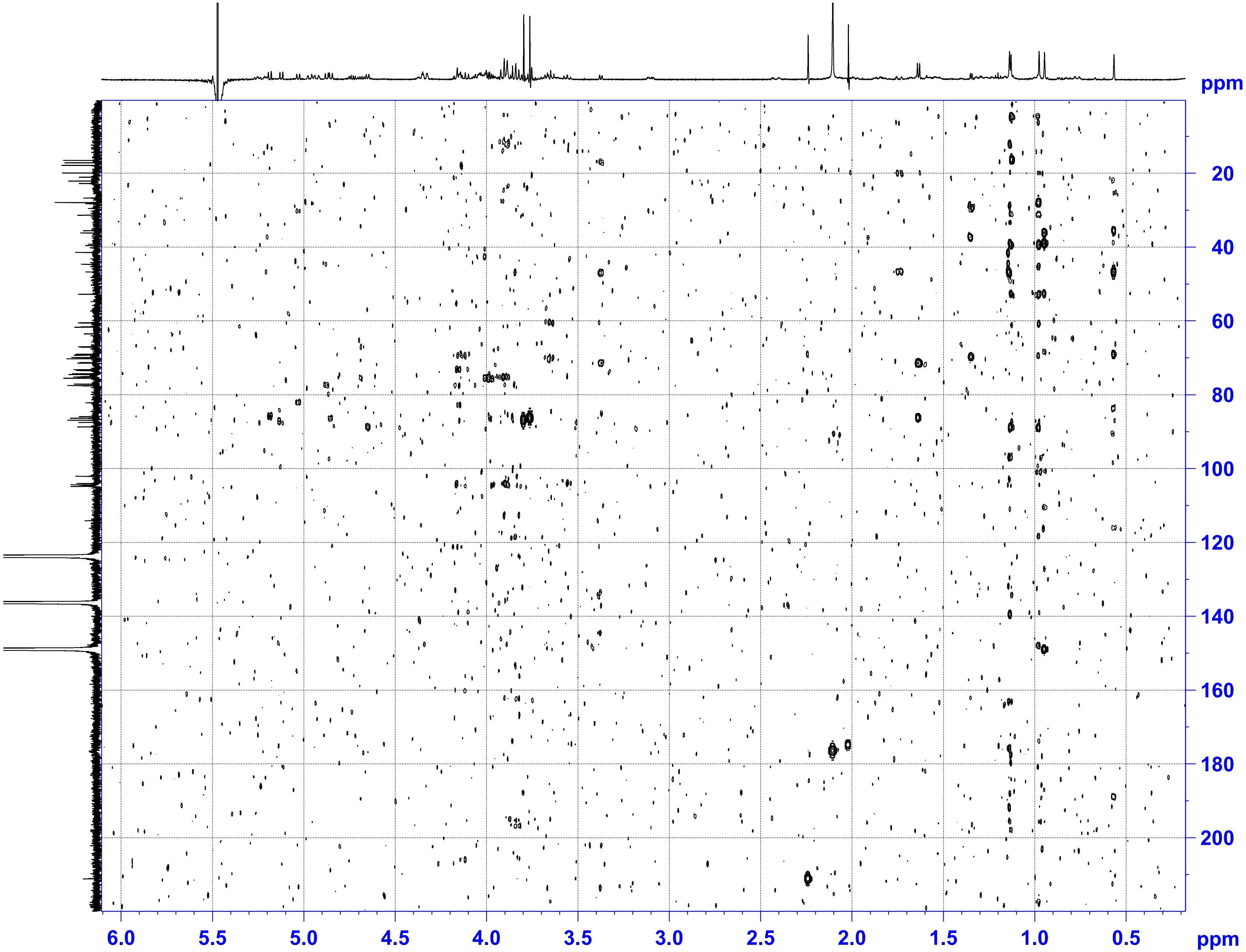

Figure S18. The HMBC (700.00 MHz) spectrum of kuriloside G (**3**) in C_5D_5N/D_2O (4/1)

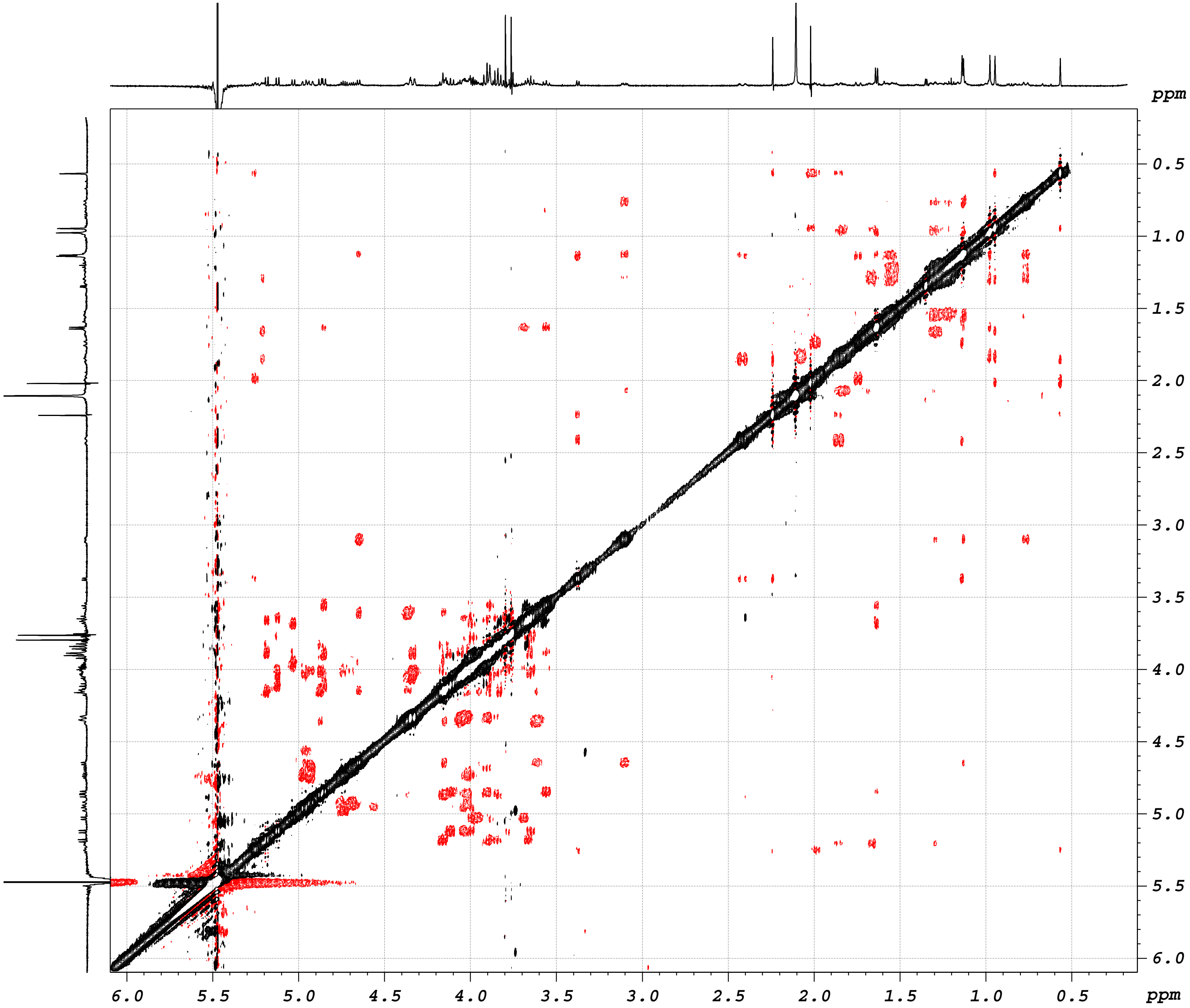

Figure S19. The ROESY (700.00 MHz) spectrum of kuriloside G (**3**) in C_5D_5N/D_2O (4/1)

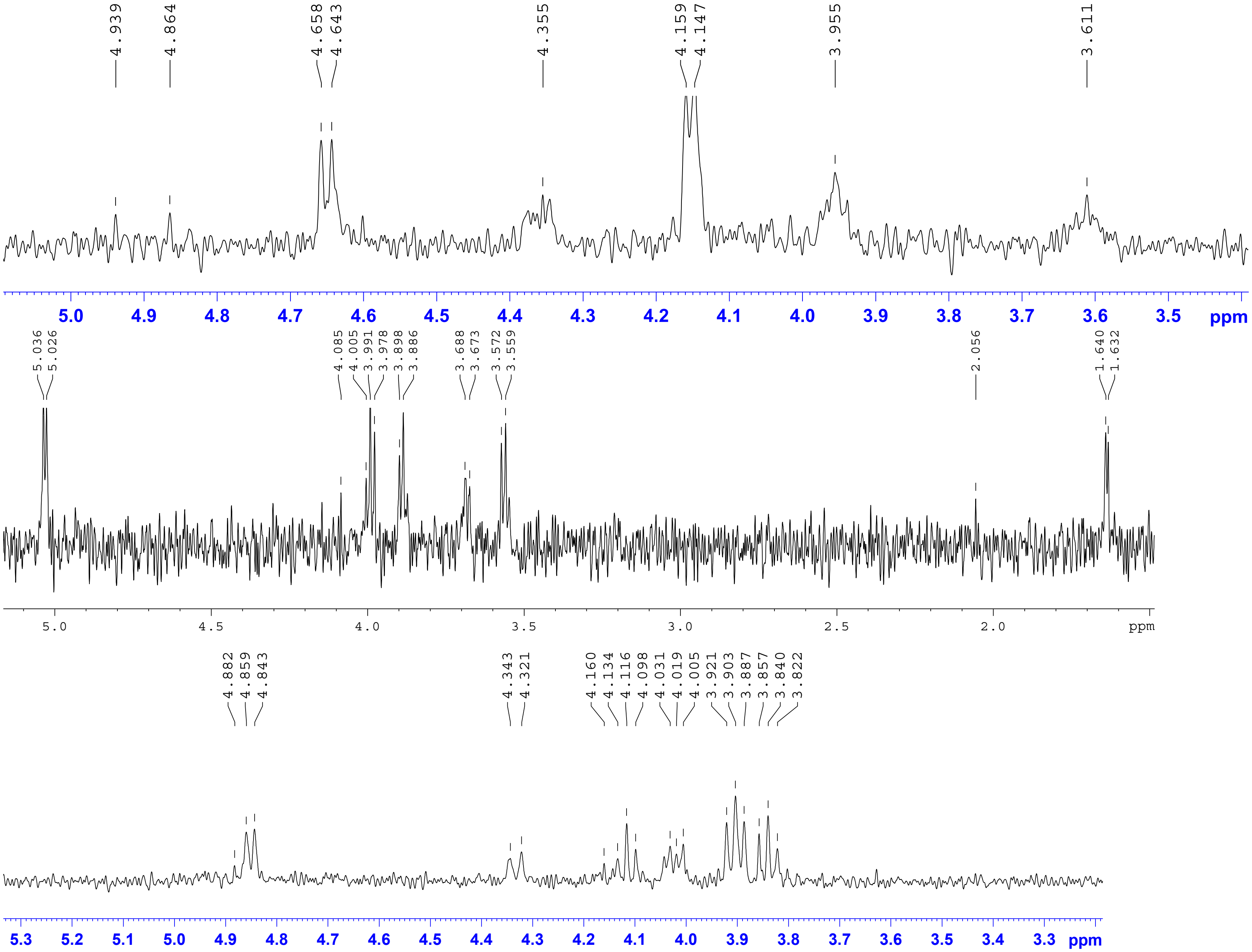

Figure S20. 1 D TOCSY (700.00 MHz) spectra of the XyloseI, QuinovoseII and GlucoseII of kuriloside G (**3**), C_5D_5N/D_2O (4/1)

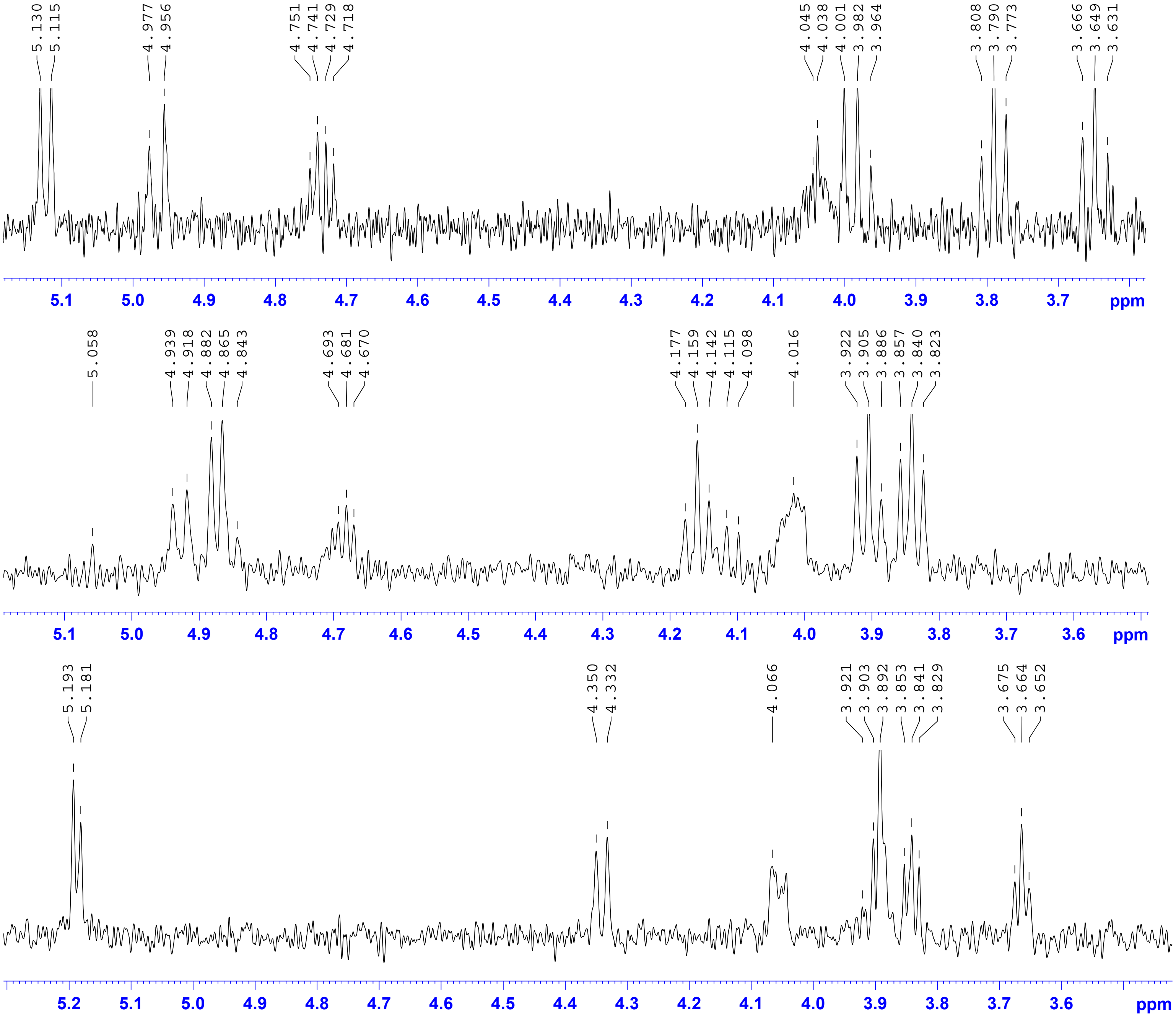

Figure S21. 1 D TOCSY (700.00 MHz) spectra of the MeGlcIV, GlucoseV and MeGlcVI of kuriloside G (**3**), C_5D_5N/D_2O (4/1)

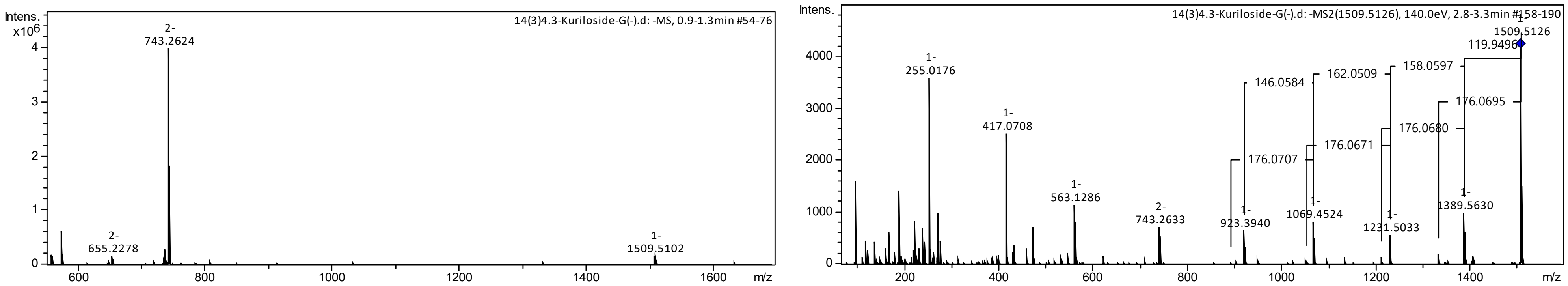

Figure S22. HR-ESI-MS and ESI-MS/MS spectra of kuriloside G (**3**)

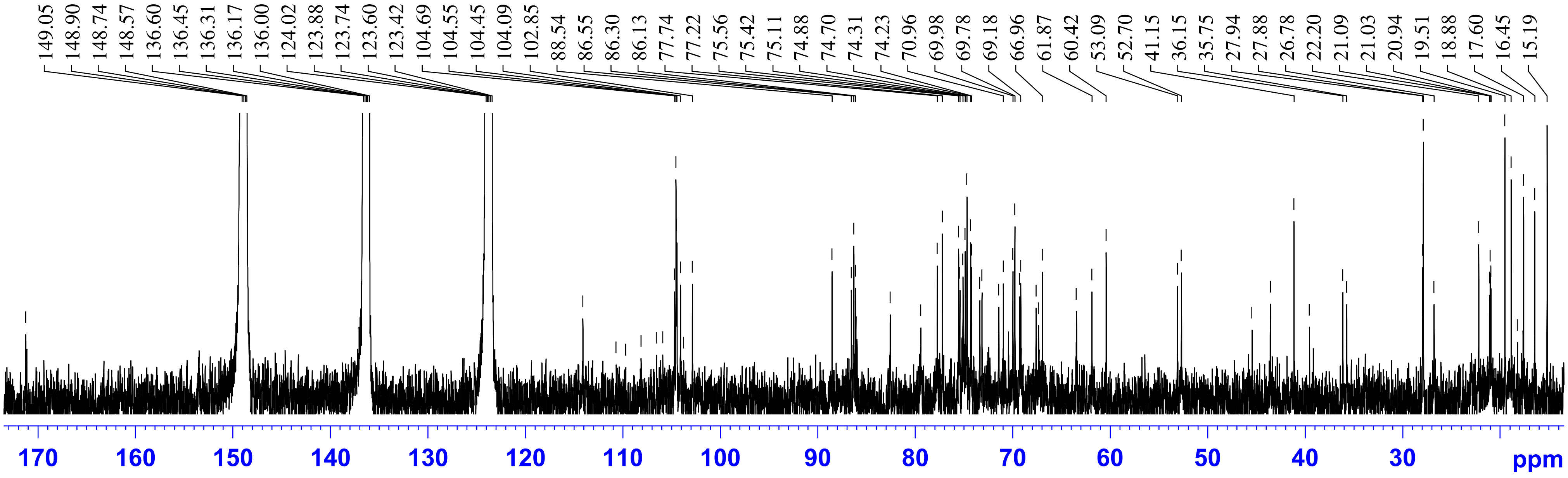

Figure S23. The ^{13}C NMR (176.03 MHz) spectrum of kuriloside H (**4**) in C_5D_5N/D_2O (4/1)

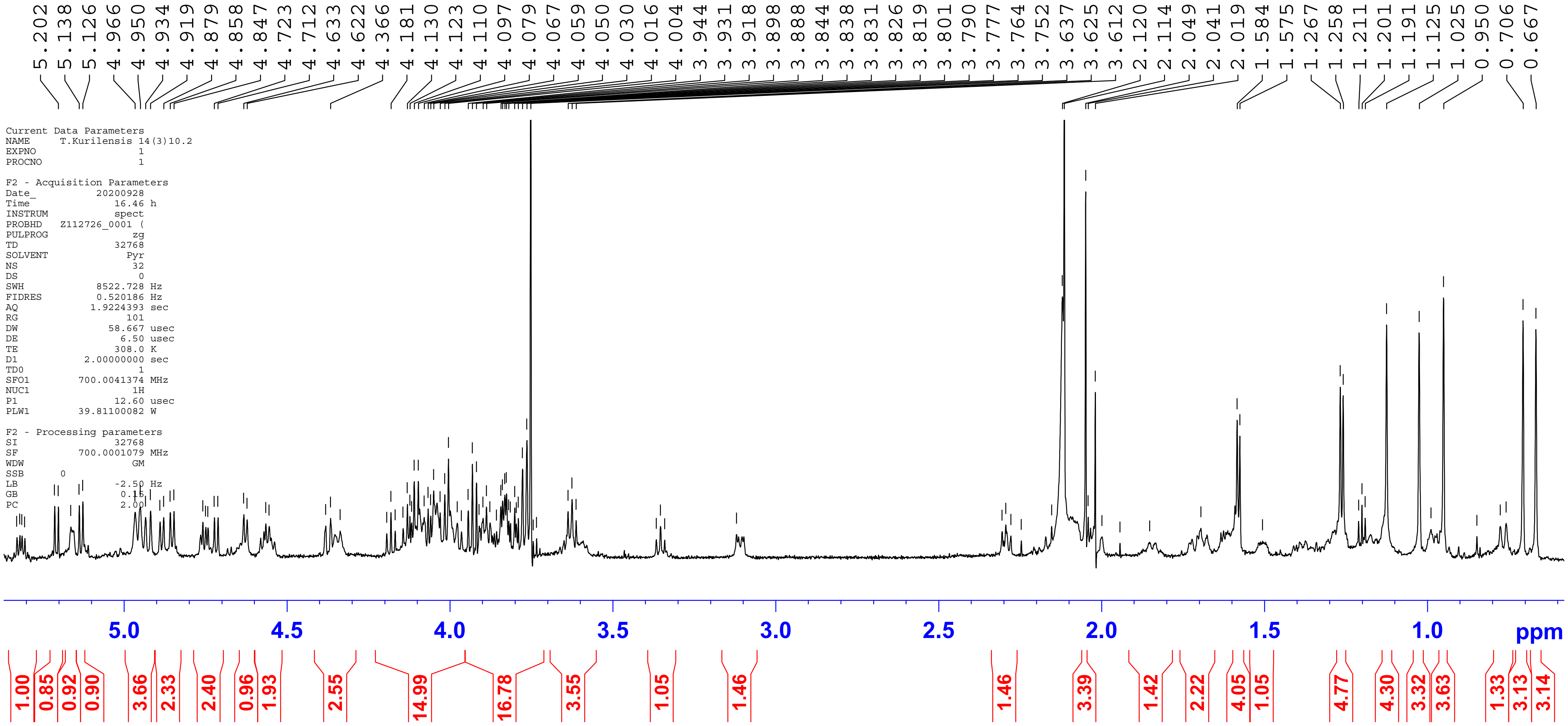

Figure S24. The ¹H NMR (700.00 MHz) spectrum of kuriloside H (**4**) in C_5D_5N/D_2O (4/1)

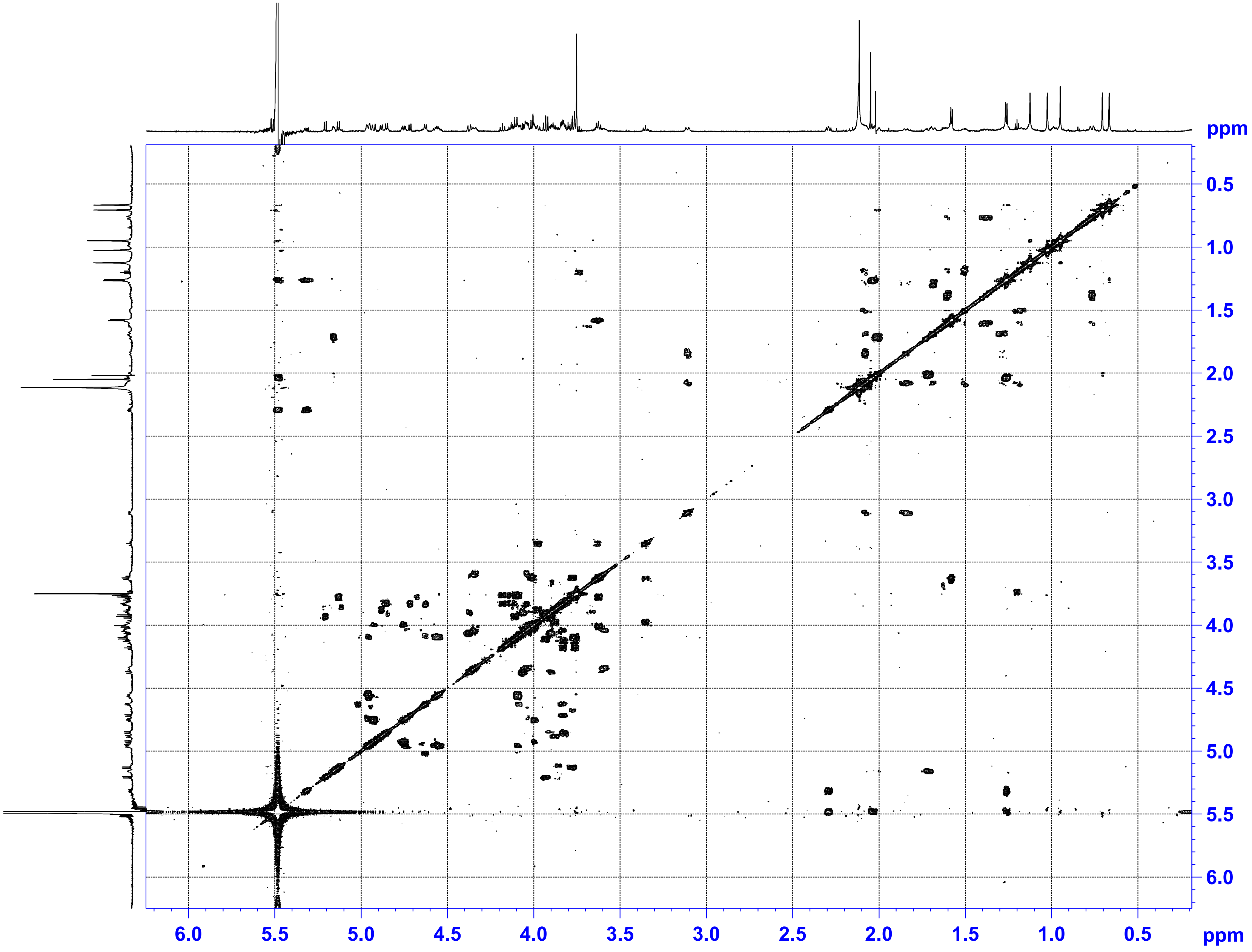

Figure S25. The COSY (700.00 MHz) spectrum of kuriloside H (**4**) in C_5D_5N/D_2O (4/1)

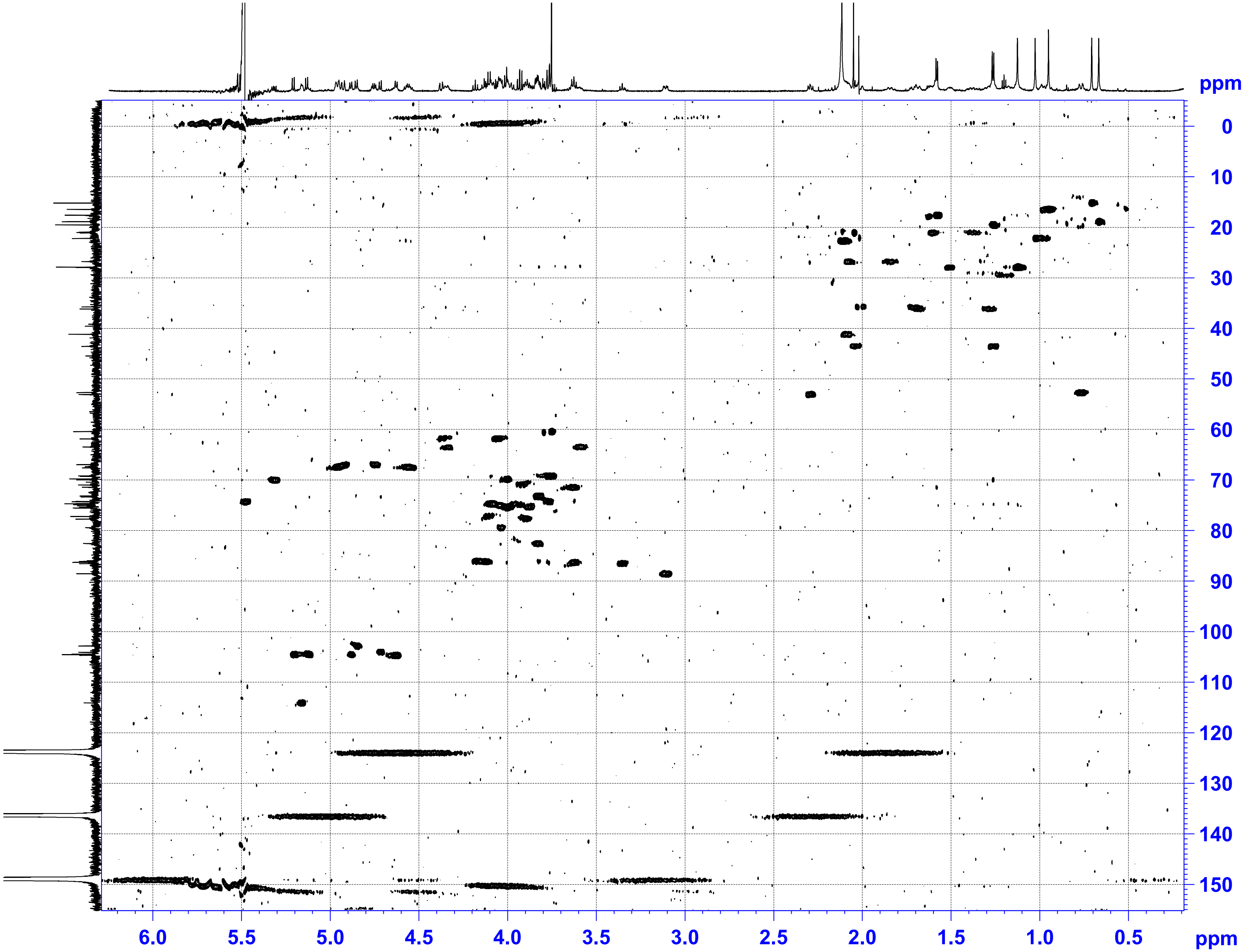

Figure S26. The HSQC (700.00 MHz) spectrum of kuriloside H (**4**) in C_5D_5N/D_2O (4/1)

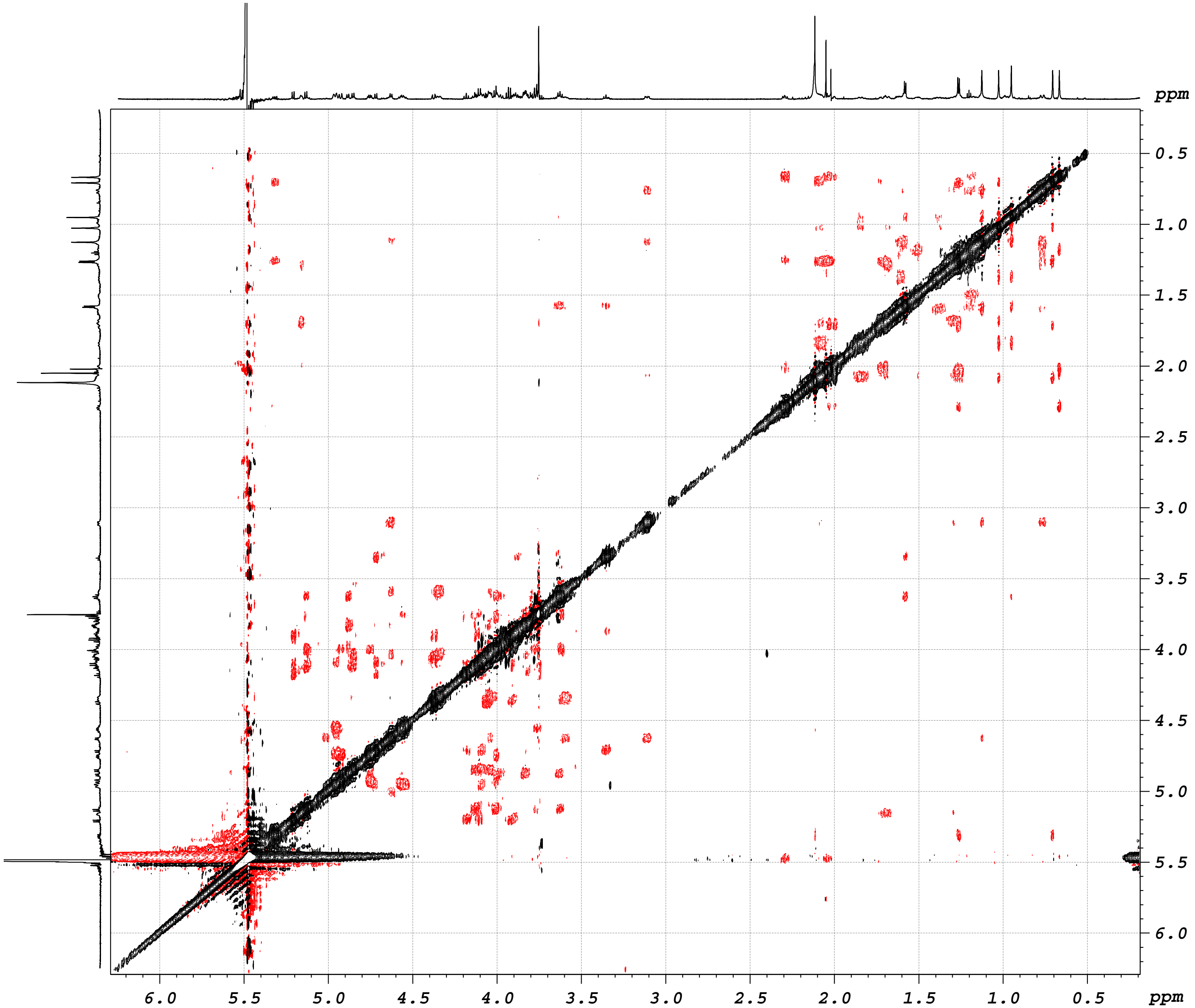

Figure S27. The ROESY (700.00 MHz) spectrum of kuriloside H (**4**) in C_5D_5N/D_2O (4/1)

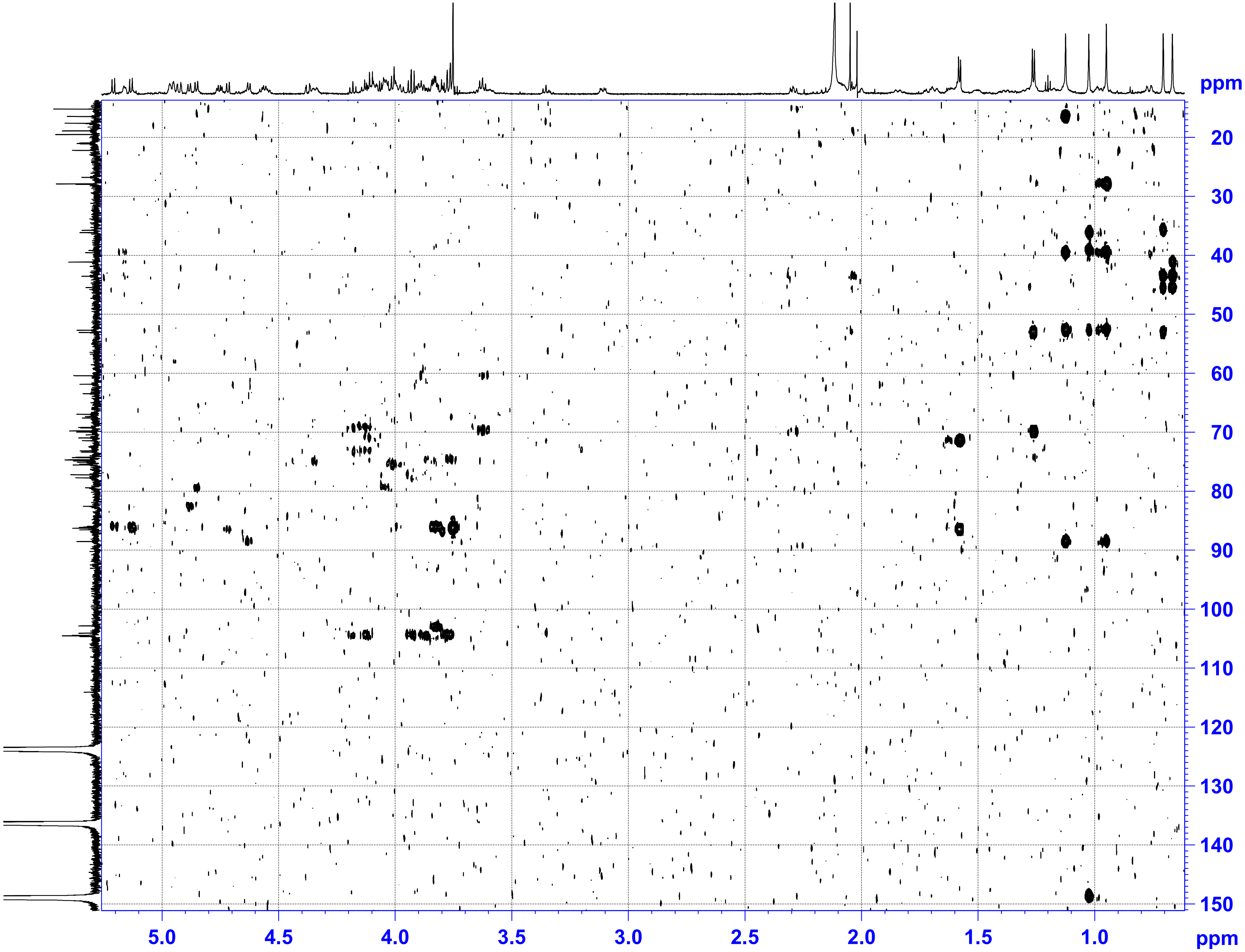

Figure S28. The HMBC (700.00 MHz) spectrum of kuriloside H (**4**) in C_5D_5N/D_2O (4/1)

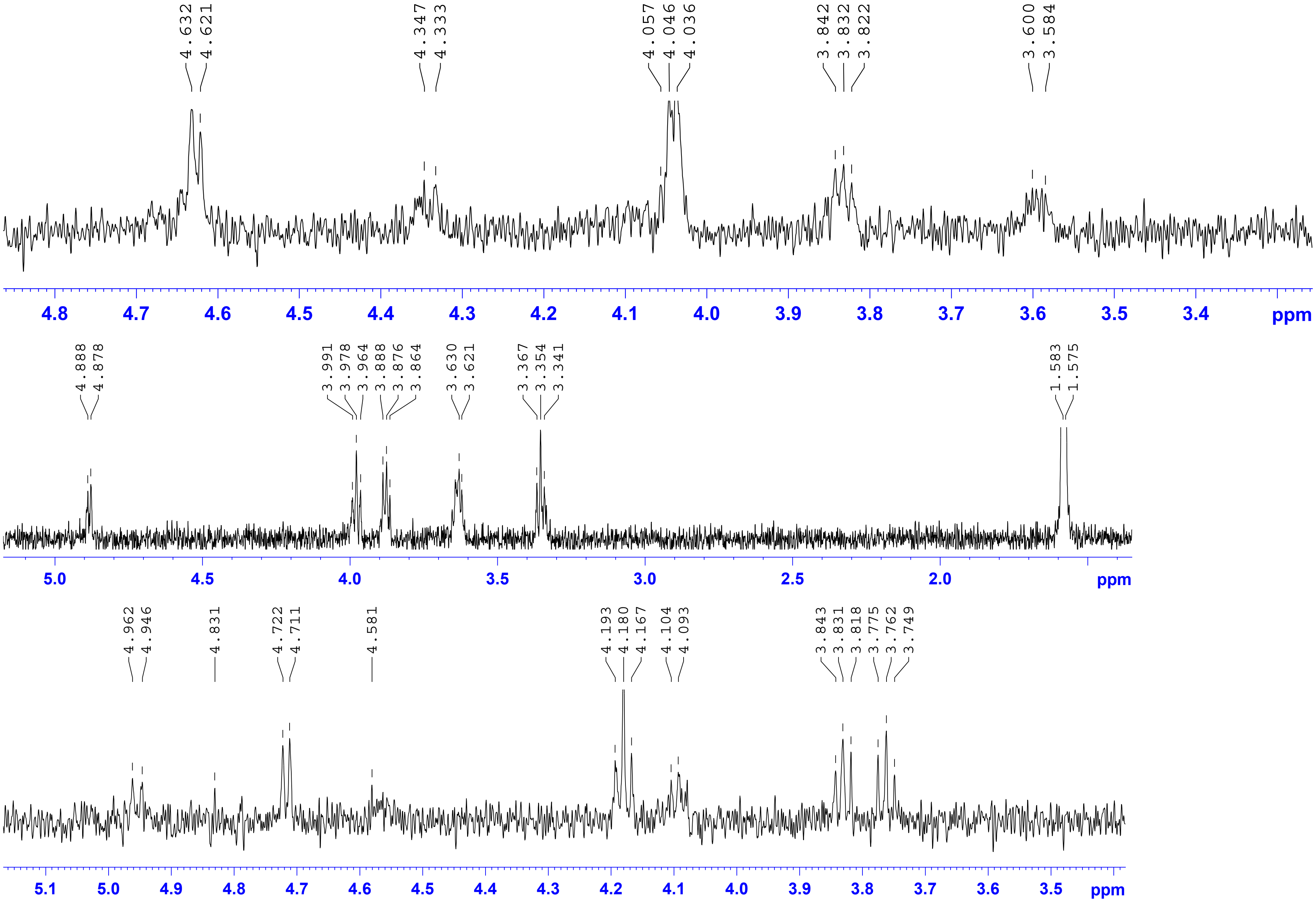

Figure S29. 1 D TOCSY (700.00 MHz) spectra of XyloseI, QuinovoseII and GlucoseIII of kuriloside H (**4**), C_5D_5N/D_2O (4/1)

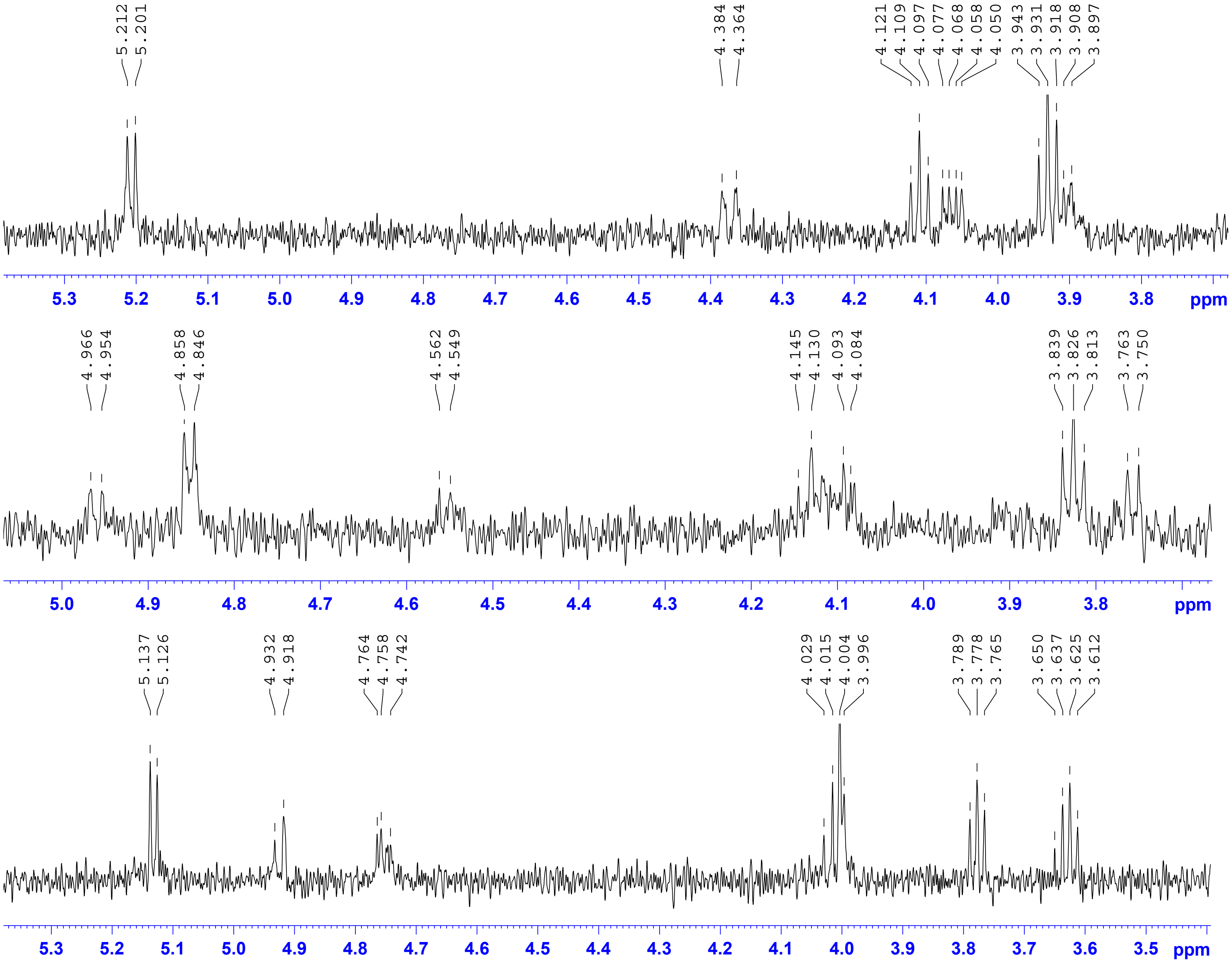

Figure S30. 1 D TOCSY (700.00 MHz) spectra of GlcIV, GlucoseV and MeGlcVI of kuriloside H (**4**) in C_5D_5N/D_2O (4/1)

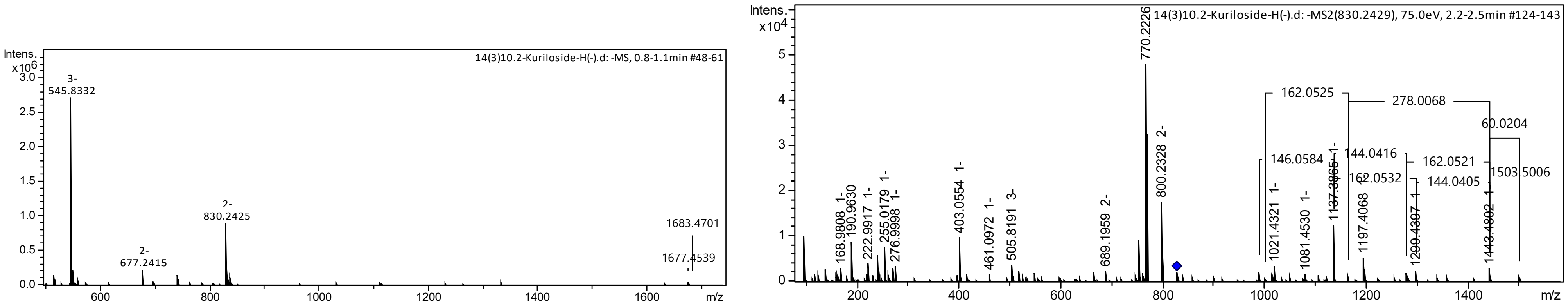

Figure S31. HR-ESI-MS and ESI-MS/MS spectra of kuriloside H (**4**)

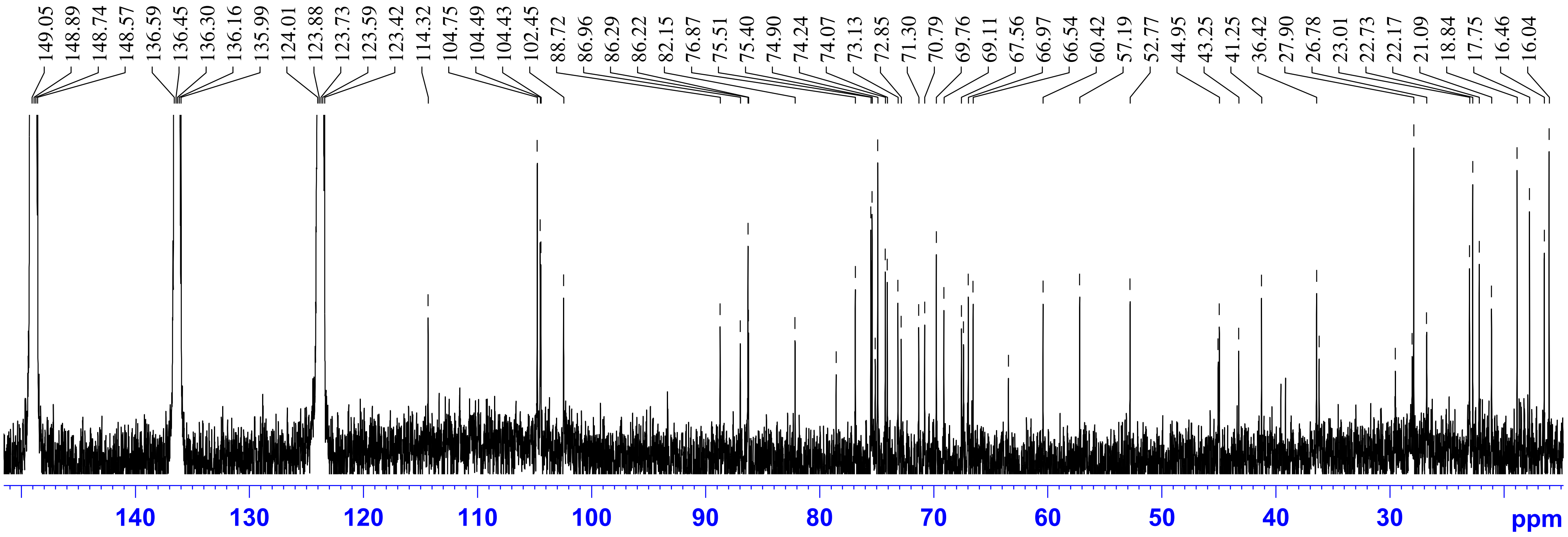

Figure S32. The ^{13}C NMR (176.03 MHz) spectrum of kuriloside I (**5**) in C_5D_5N/D_2O (4/1)

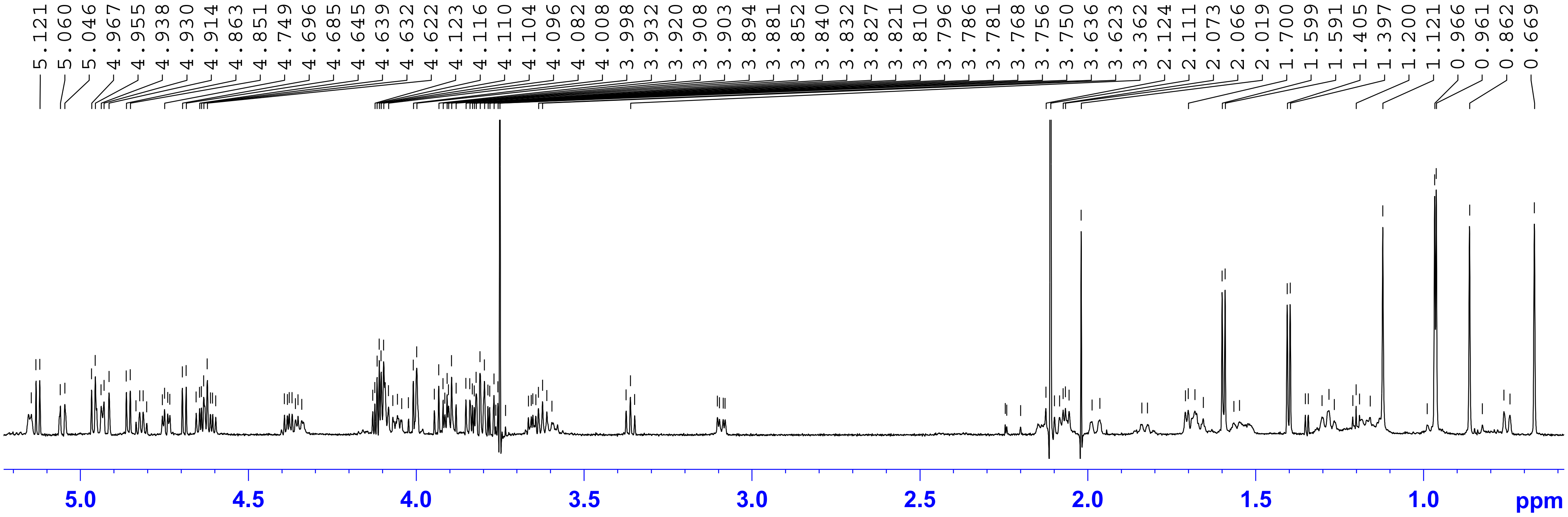

Figure S33. The ^{1}H NMR (700.00 MHz) spectrum of kuriloside I (**5**) in C_5D_5N/D_2O (4/1)

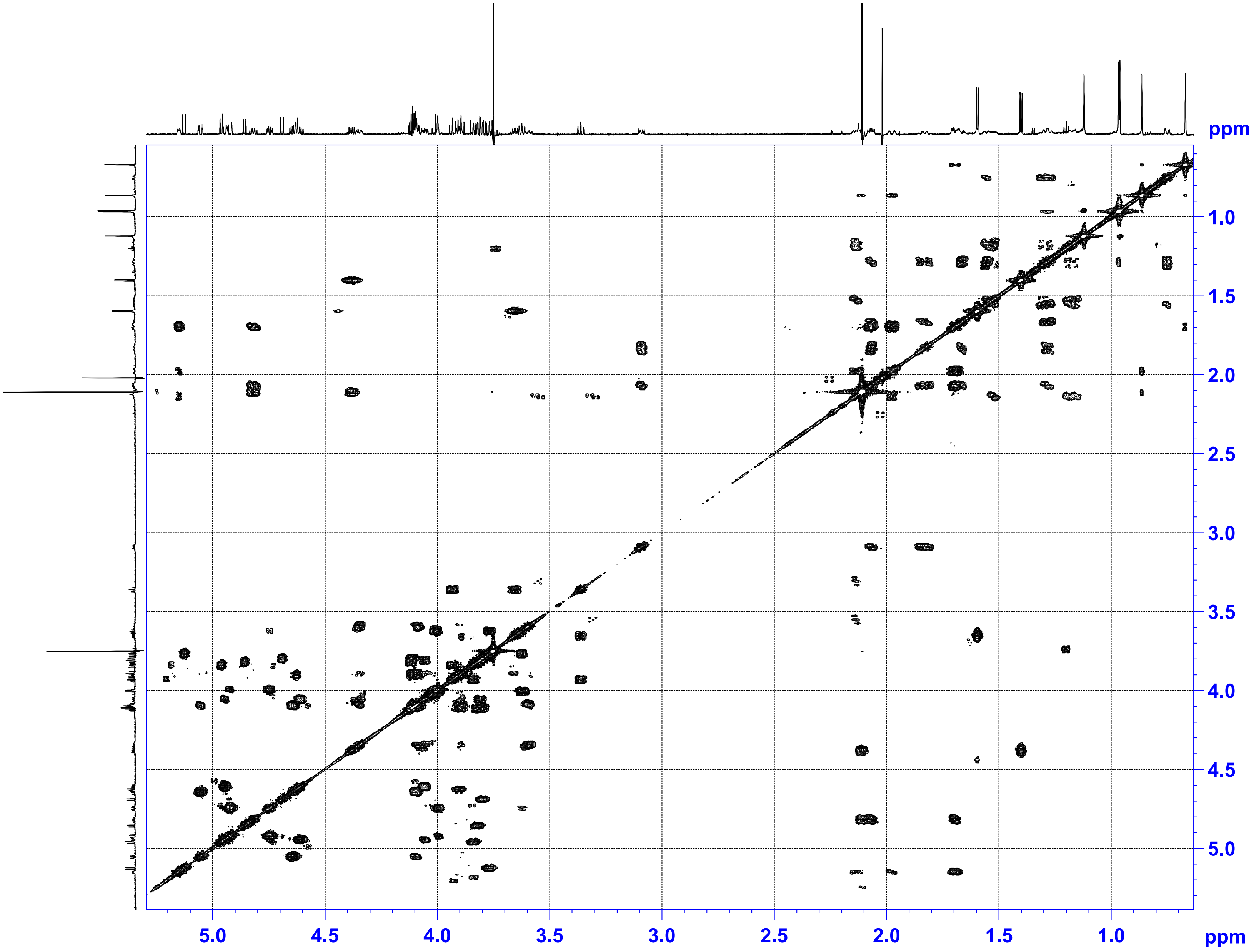

Figure S34. The COSY (700.00 MHz) spectrum of kuriloside I (**5**) in C_5D_5N/D_2O (4/1)

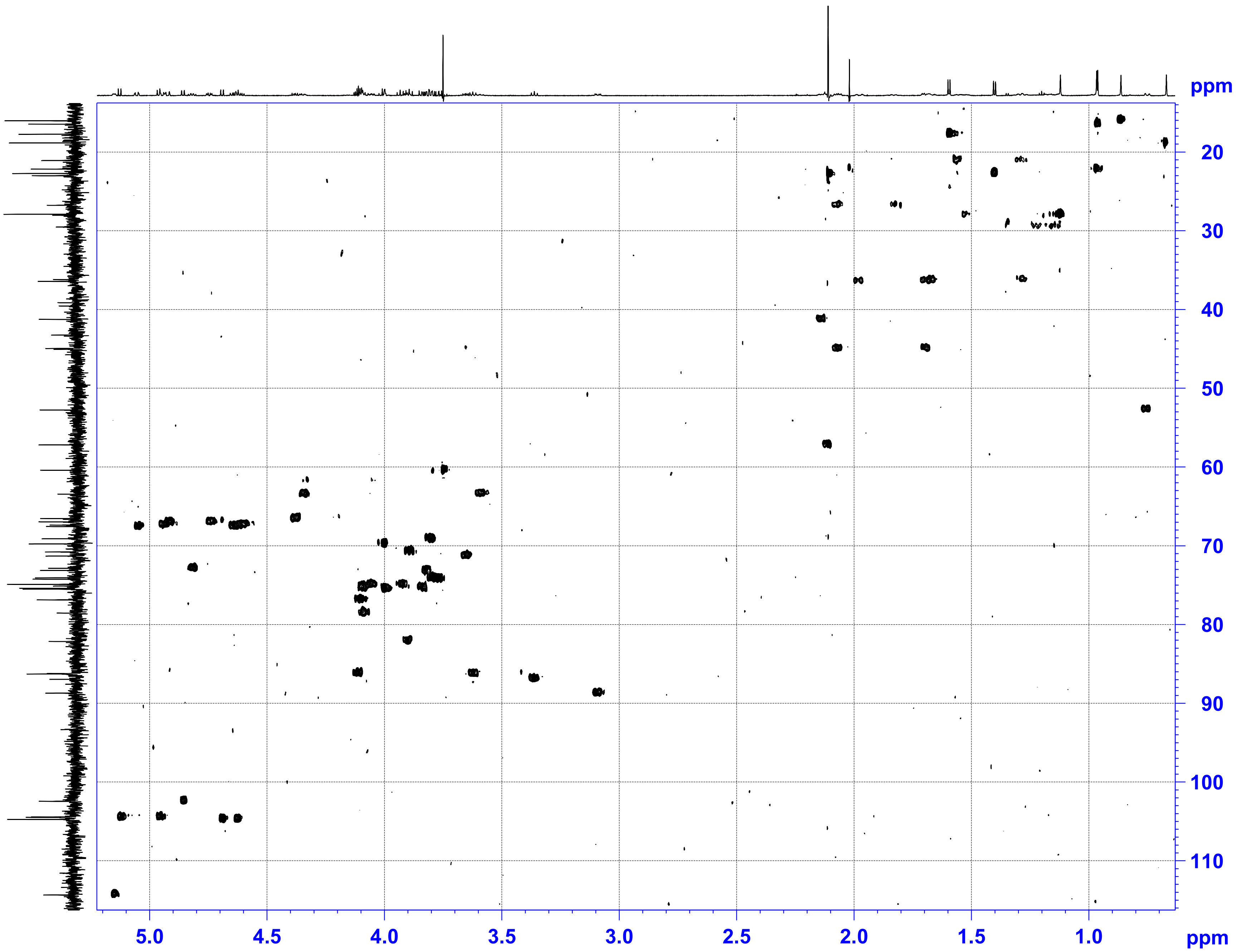

Figure S35. The HSQC (700.00 MHz) spectrum of kuriloside I (**5**) in C_5D_5N/D_2O (4/1)

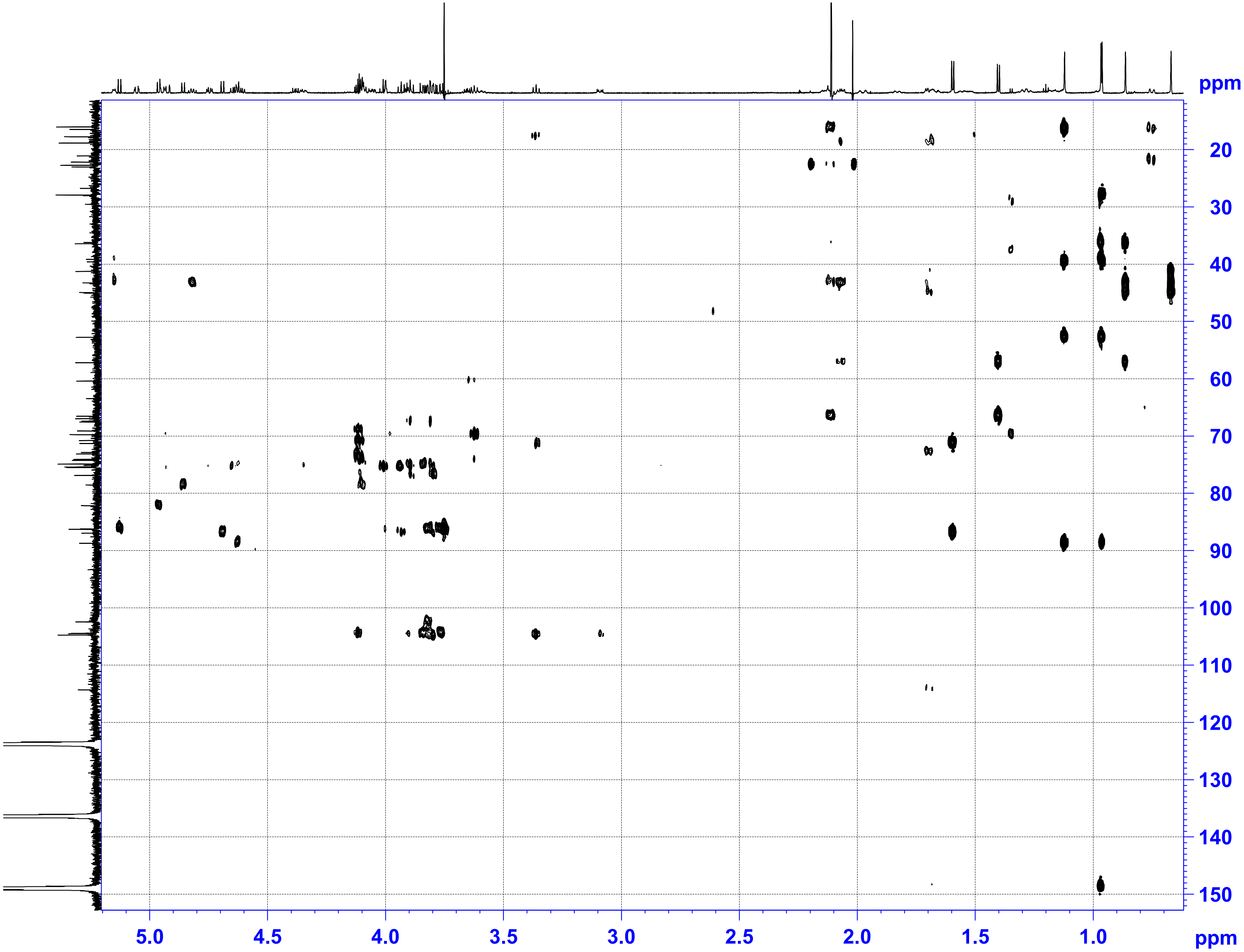

Figure S36. The HMBC (700.00 MHz) spectrum of kuriloside I (**5**) in C_5D_5N/D_2O (4/1)

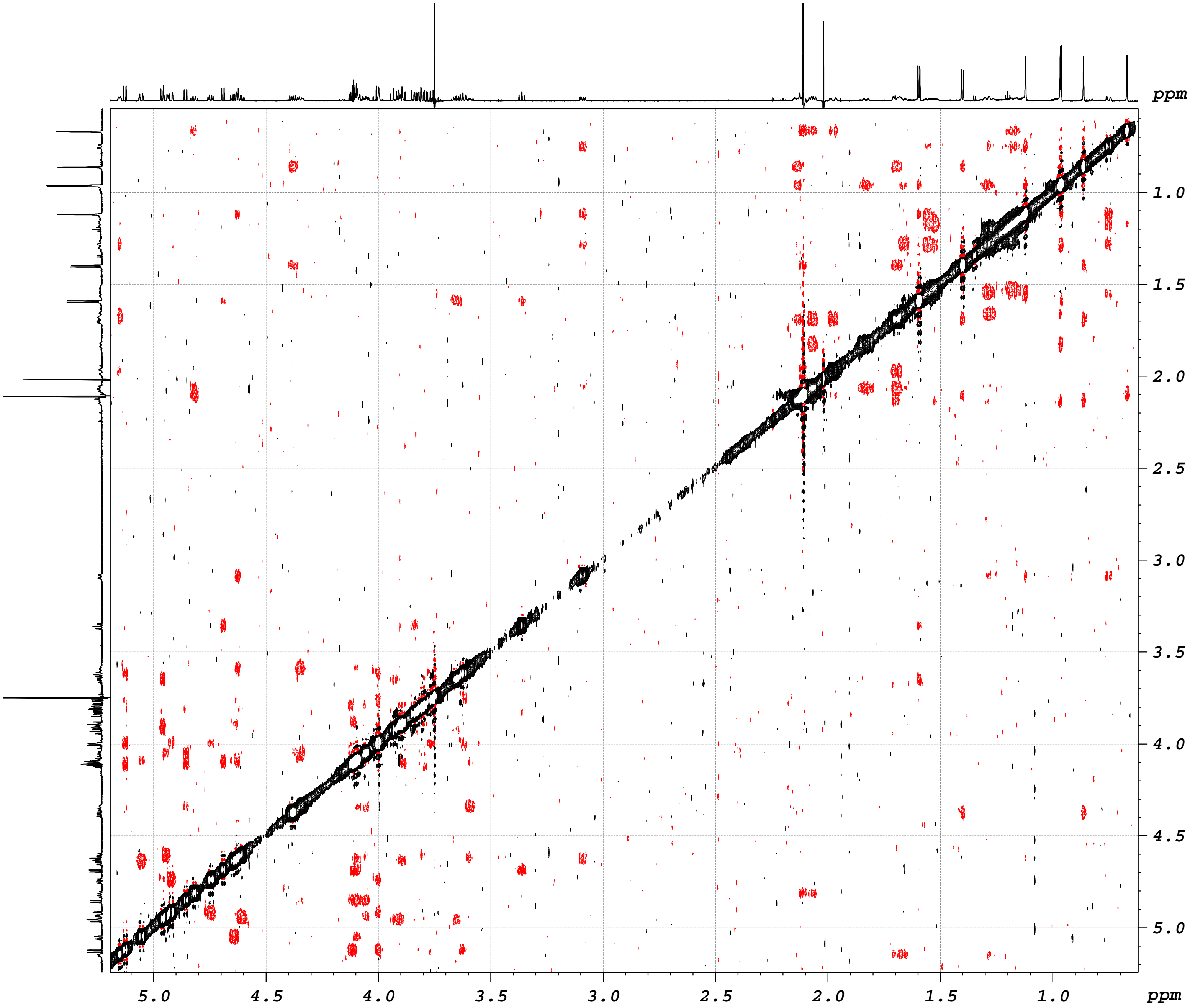

Figure S37. The ROESY (700.00 MHz) spectrum of kuriloside I (**5**) in C_5D_5N/D_2O (4/1)

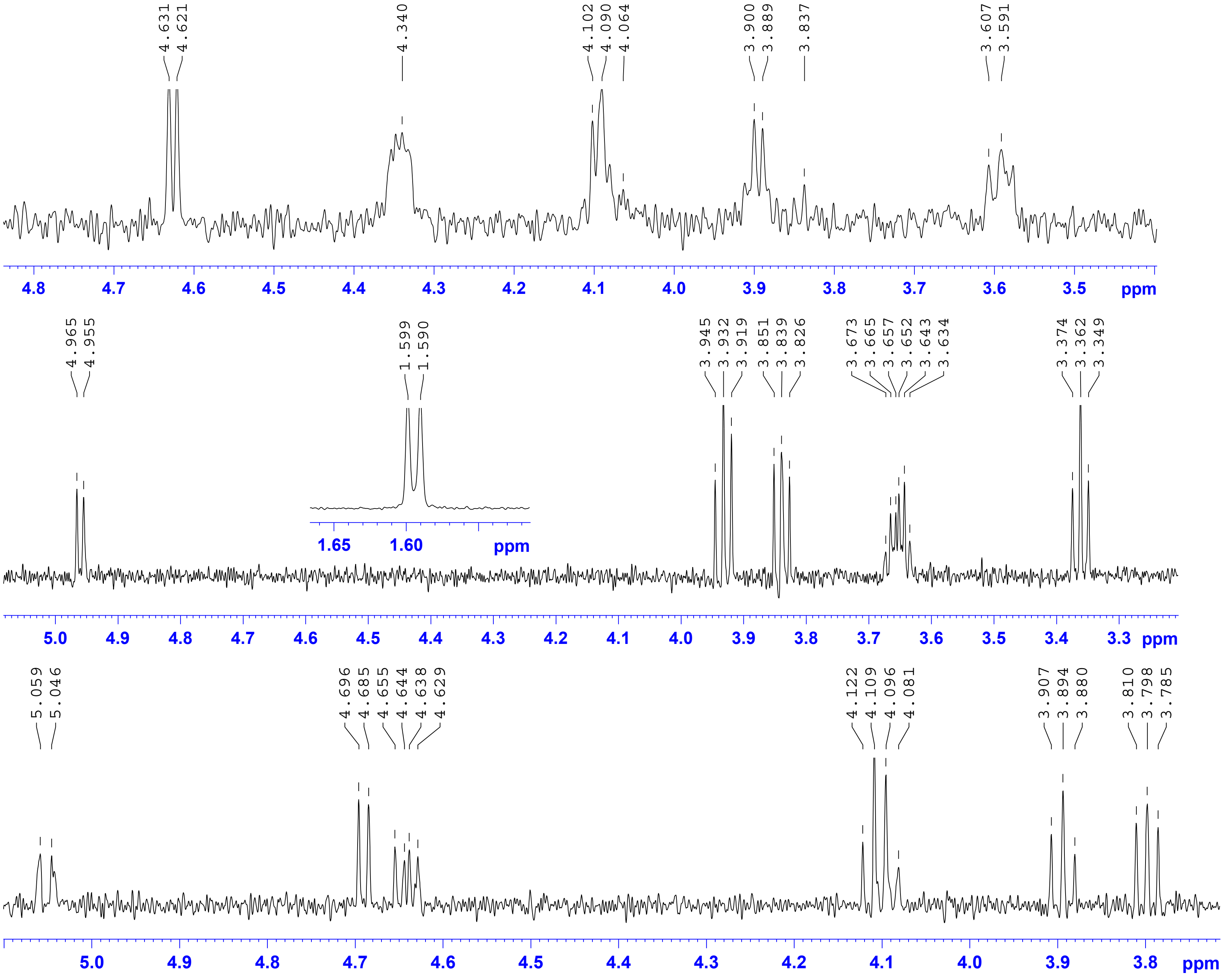

Figure S38. 1D TOCSY (700.00 MHz) spectra of XyloseI, QuinovoseII and GlucoseIII kuriloside I (**5**) in C_5D_5N/D_2O (4/1)

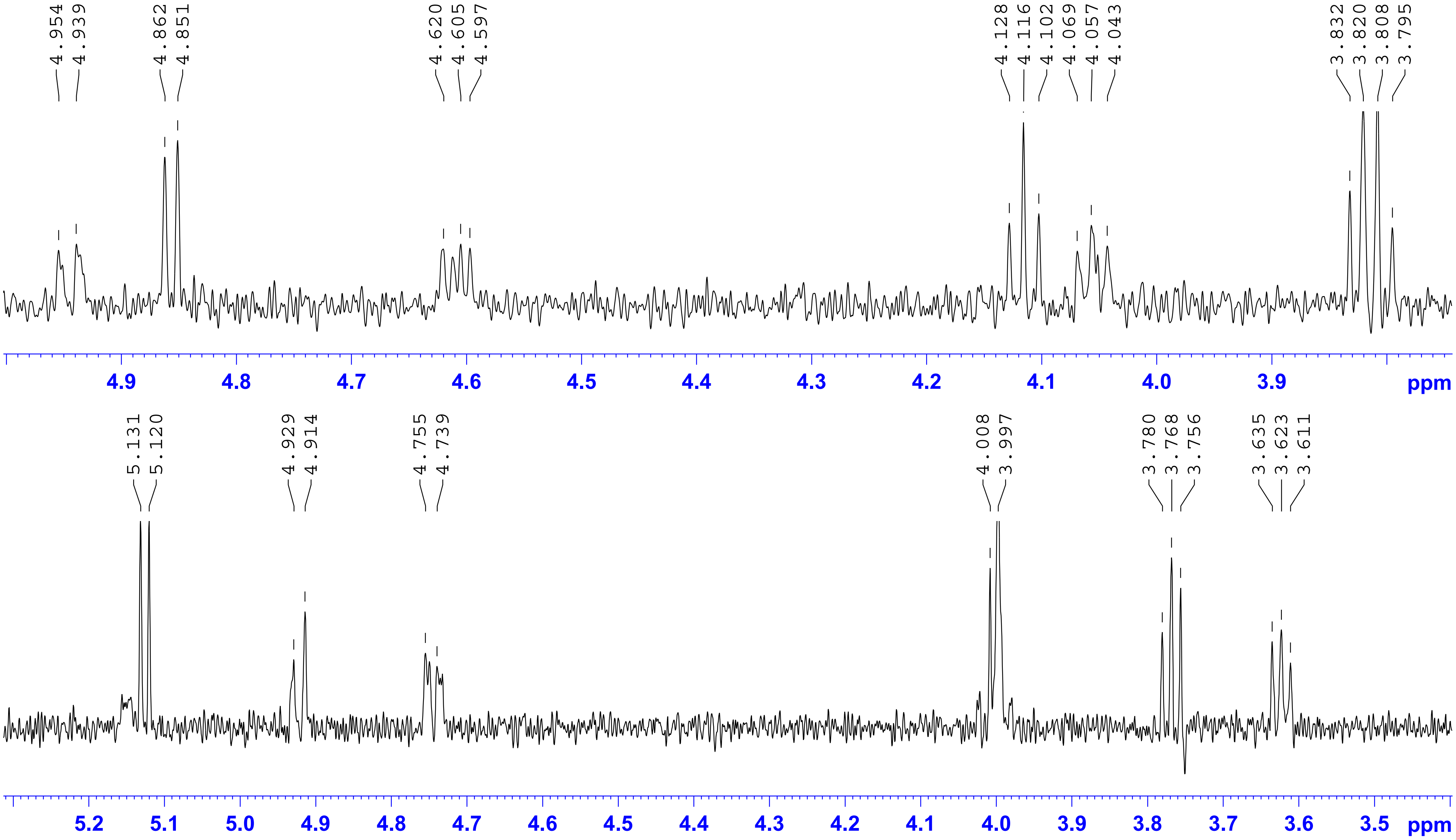

Figure S39. 1D TOCSY (700.00 MHz) spectra of GlucoseIV and MeGlcV of kuriloside I (**5**), C_5D_5N/D_2O (4/1)

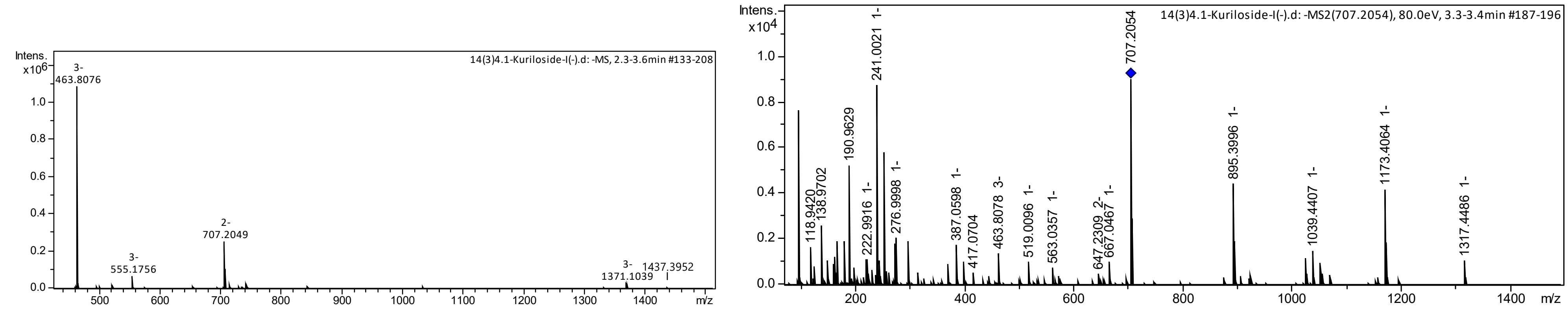

Figure S40. HR-ESI-MS and ESI-MS/MS spectra of kuriloside I (**5**)

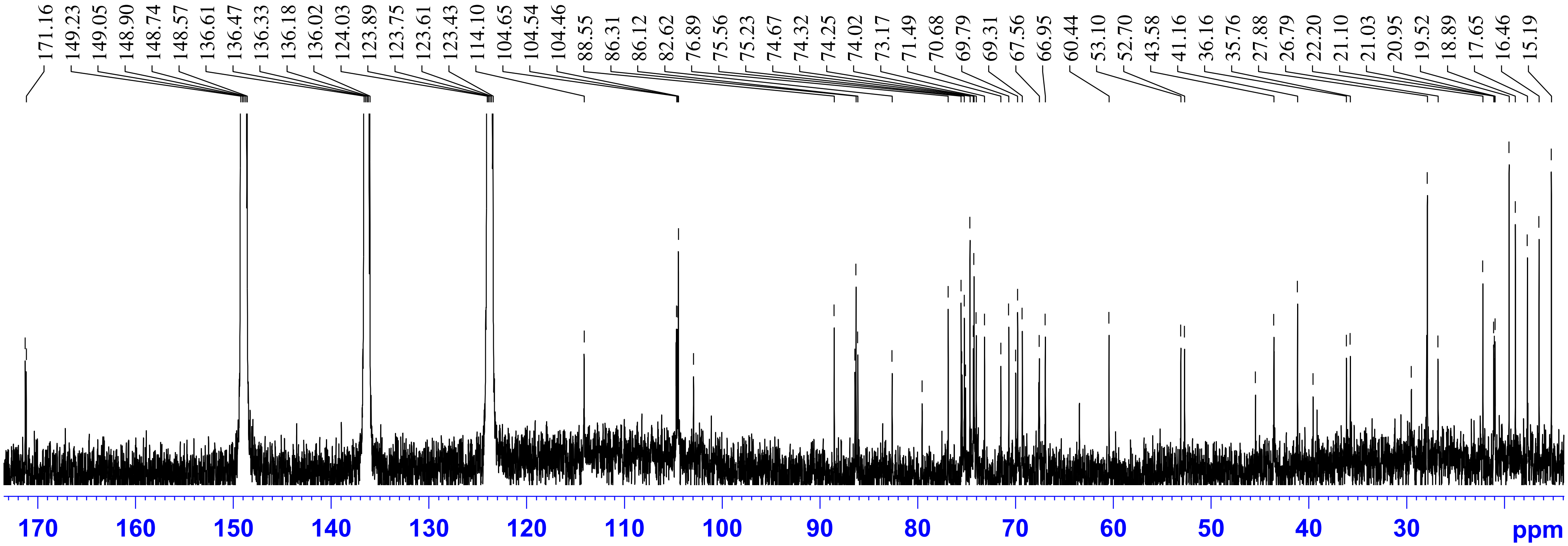

Figure S41. The ^{13}C NMR (176.03 MHz) spectrum of kuriloside I_1 (**6**) in C_5D_5N/D_2O (4/1)

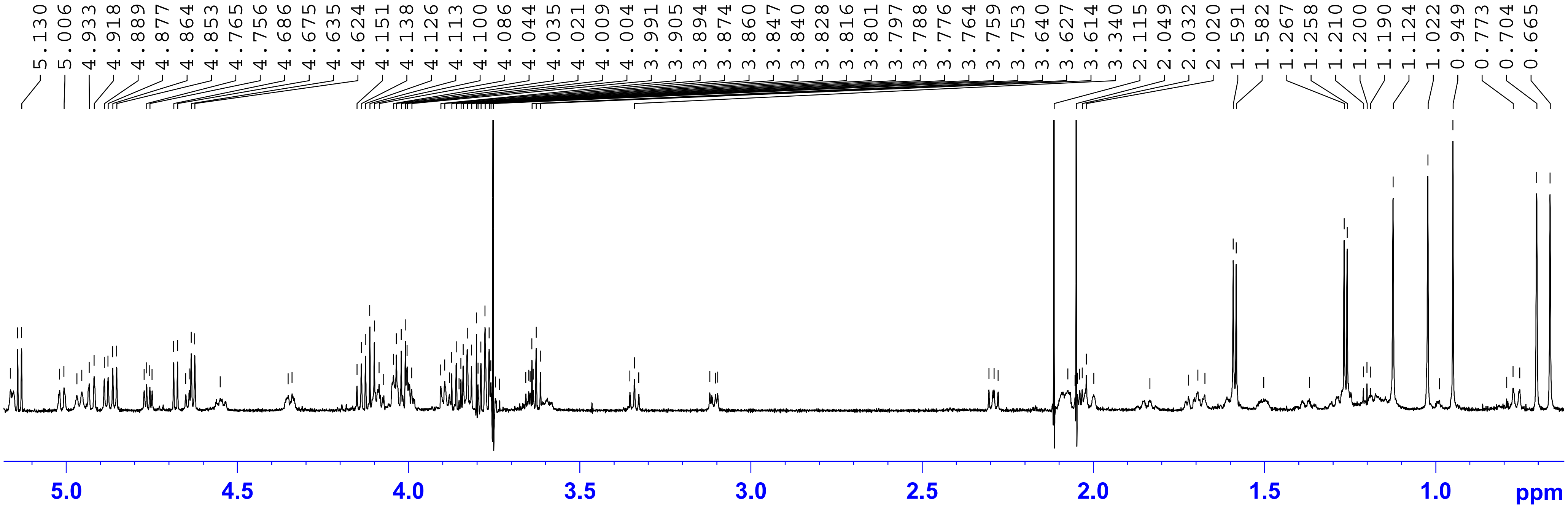

Figure S42. The ^{1}H NMR (700.00 MHz) spectrum of kuriloside I1 (**6**) in C_5D_5N/D_2O (4/1)

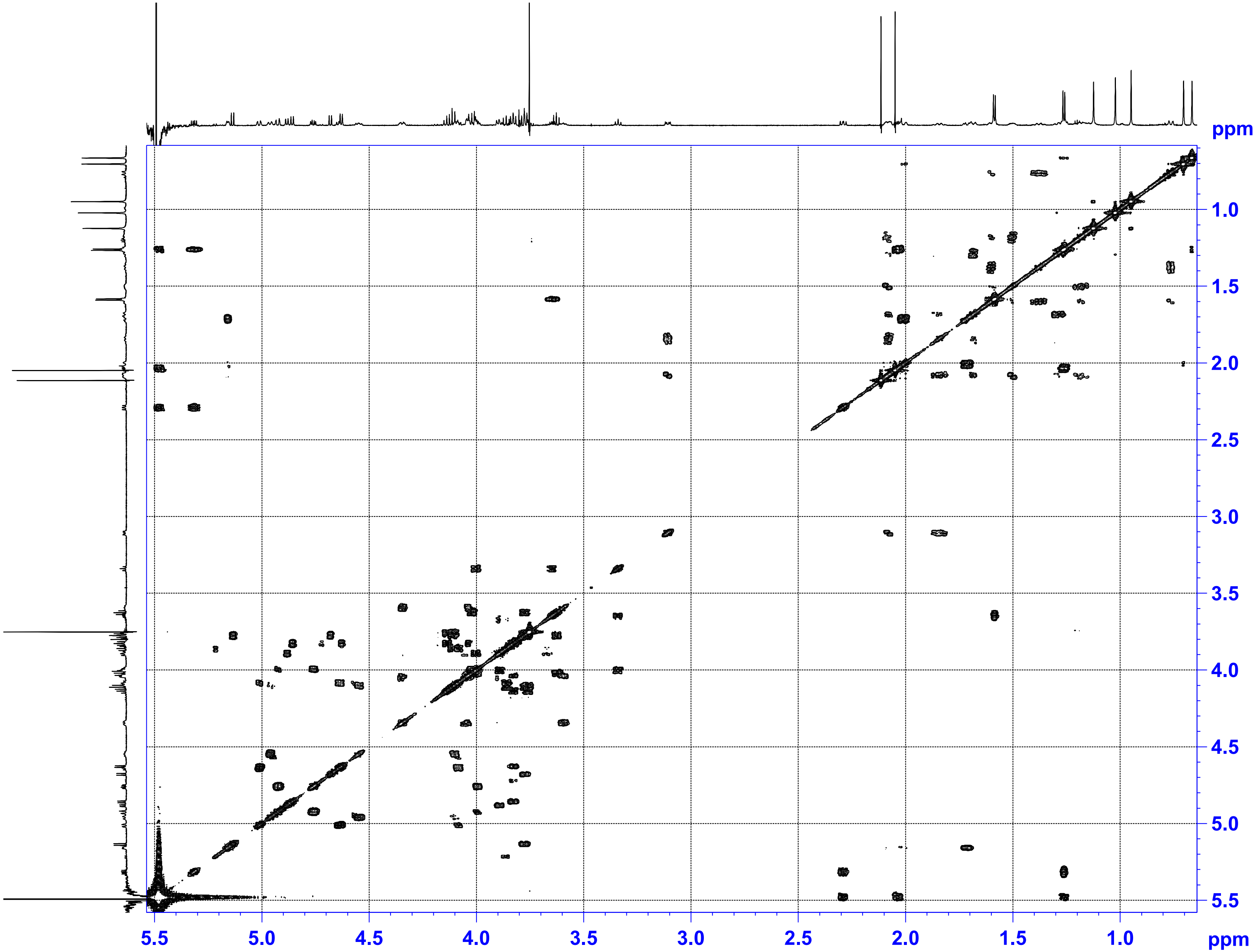

Figure S43. The COSY (700.00 MHz) spectrum of kuriloside I_1 (**6**) in C_5D_5N/D_2O (4/1)

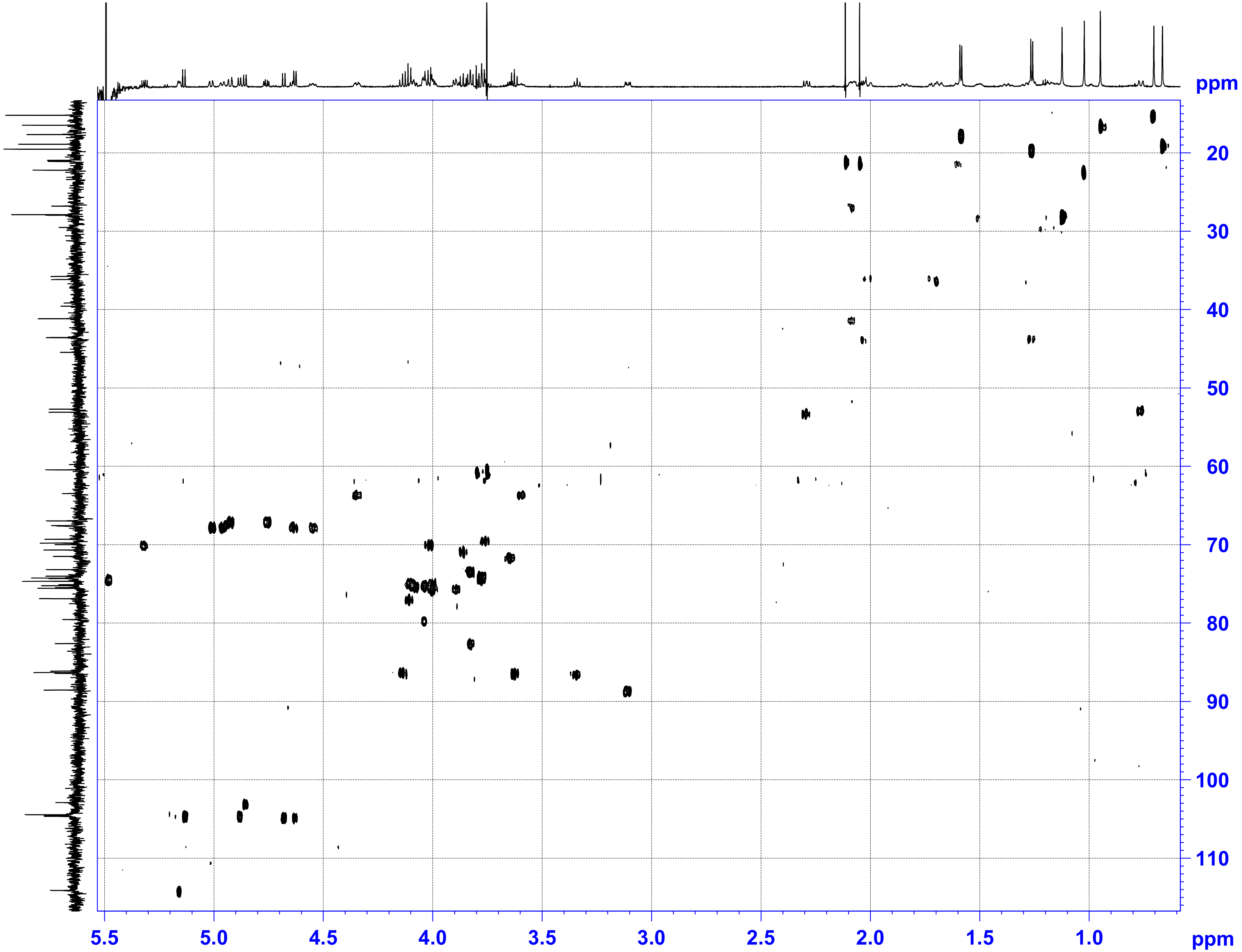

Figure S44. The HSQC (700.00 MHz) spectrum of kuriloside I1 (**6**) in C_5D_5N/D_2O (4/1)

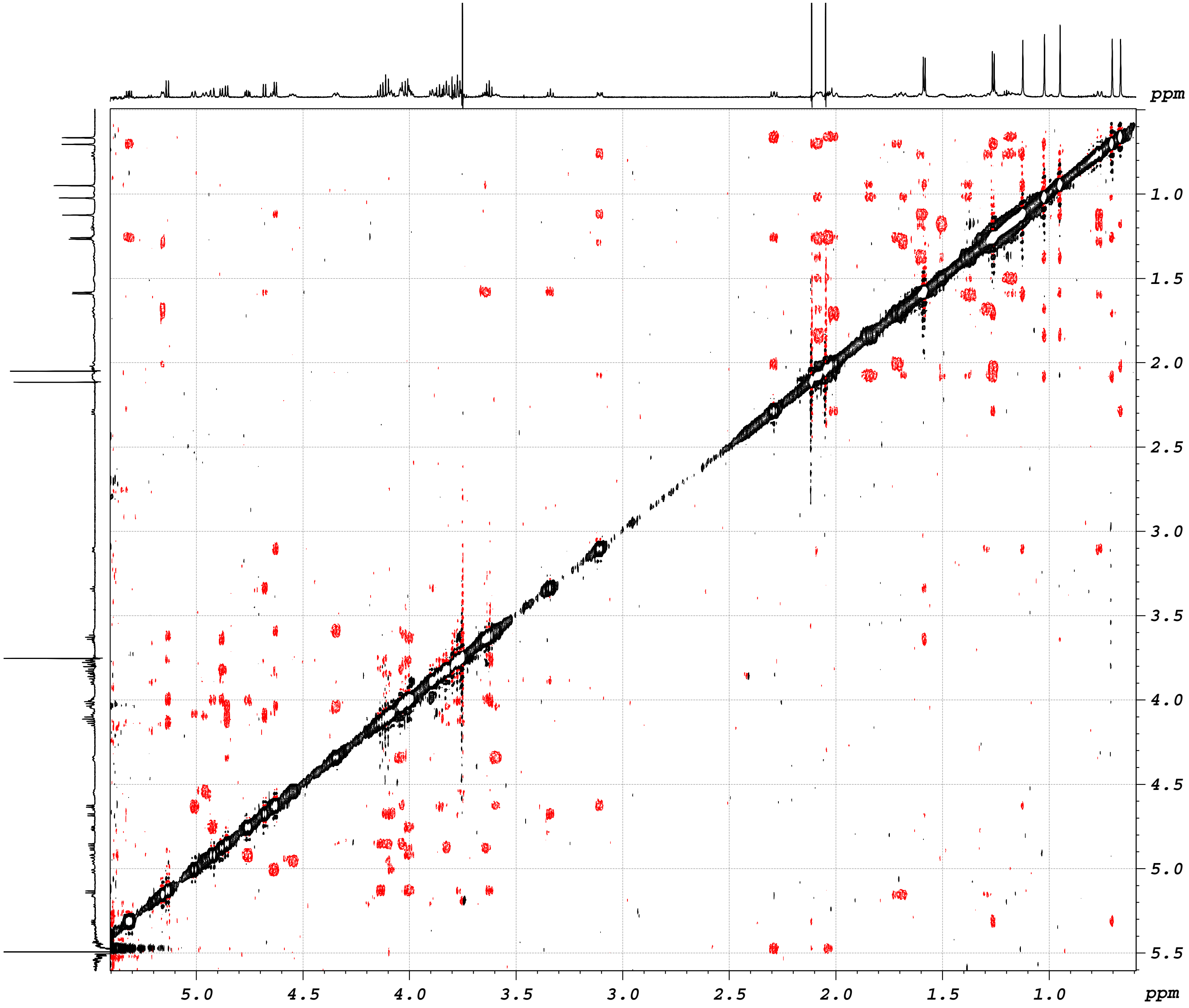

Figure S45. The ROESY (700.00 MHz) spectrum of kuriloside I_1 (**6**) in C_5D_5N/D_2O (4/1)

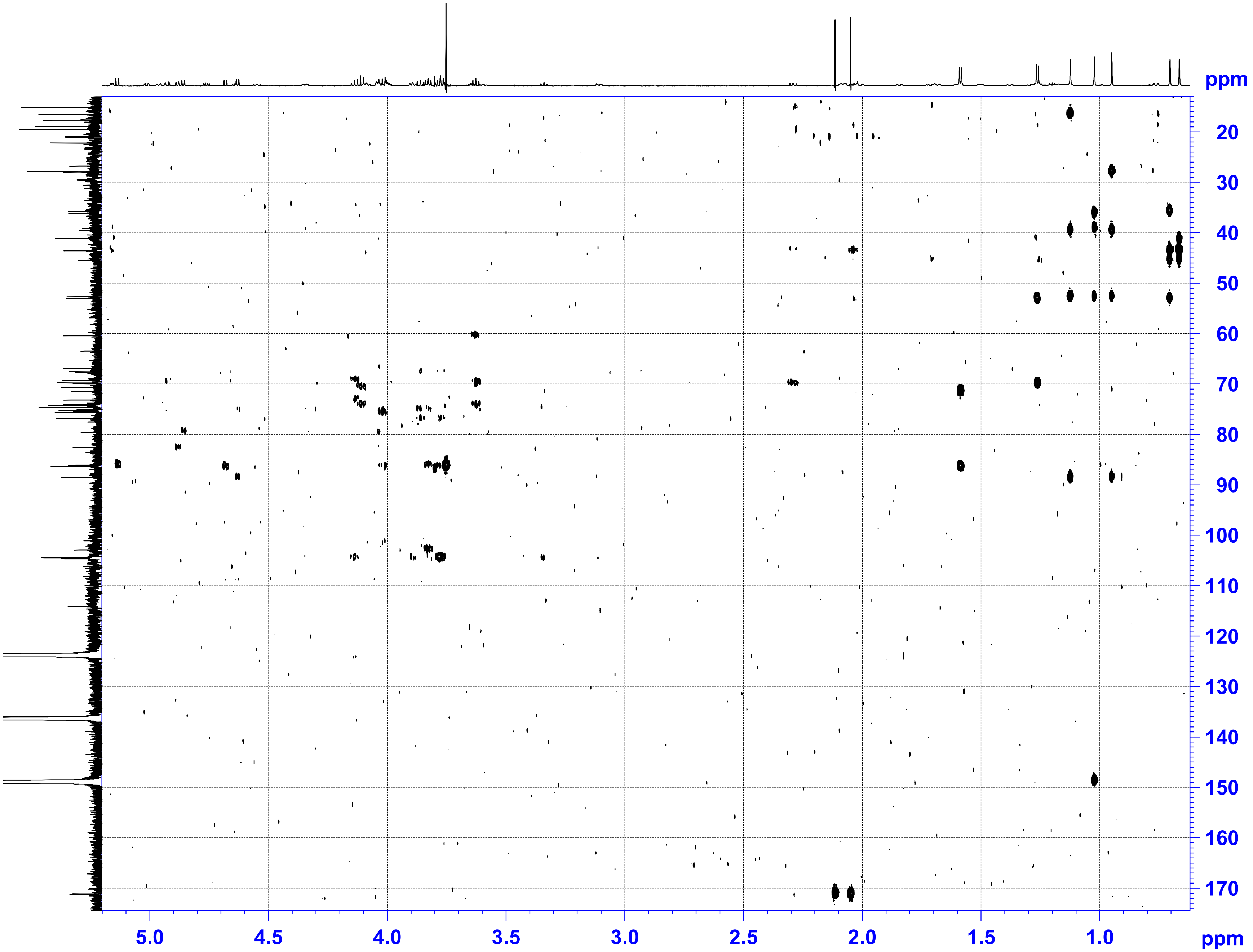

Figure S46. The HMBC (700.00 MHz) spectrum of kuriloside I$_1$ (**6**) in C_5D_5N/D_2O (4/1)

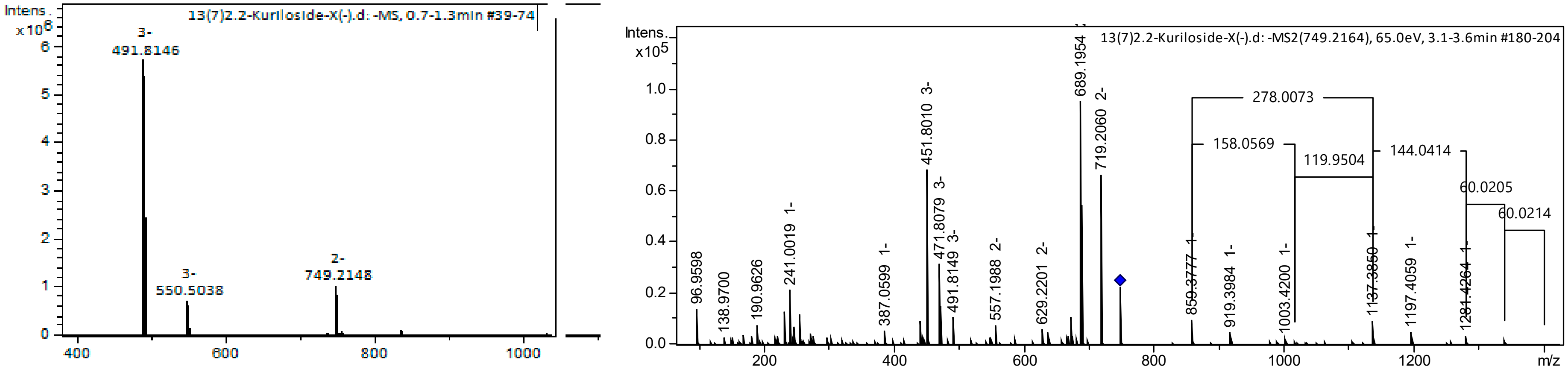

Figure S47. HR-ESI-MS and ESI-MS/MS spectra of kuriloside I1 (**6**)

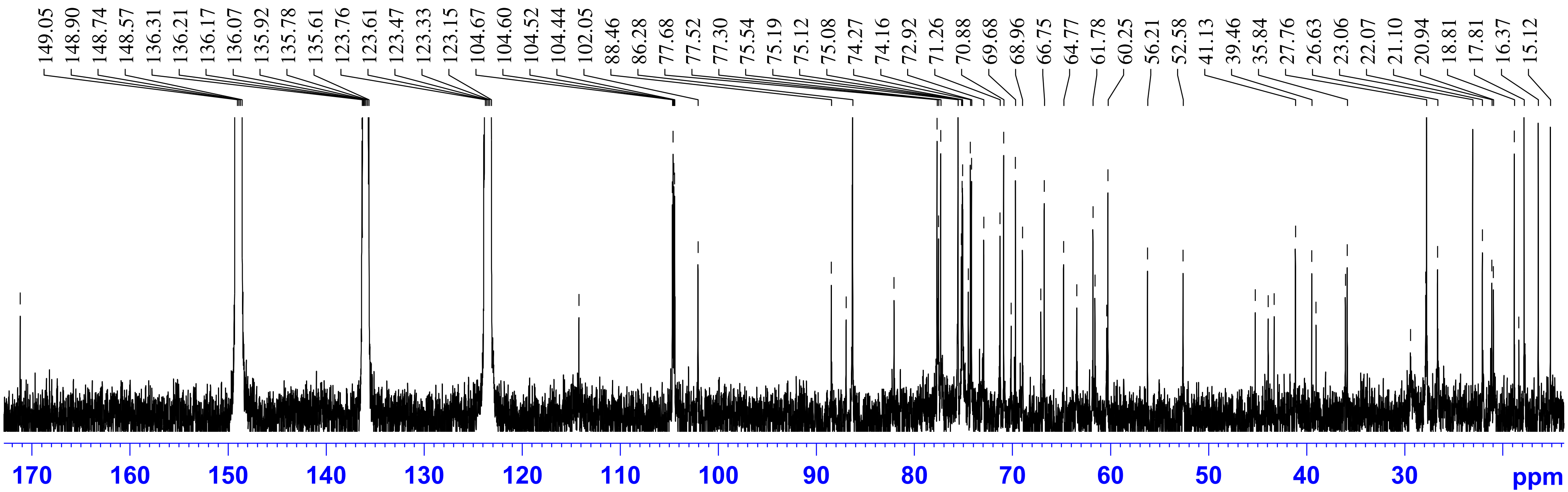

Figure S48. The ^{13}C NMR (176.03 MHz) spectrum of kuriloside J (**7**) in C_5D_5N/D_2O (4/1)

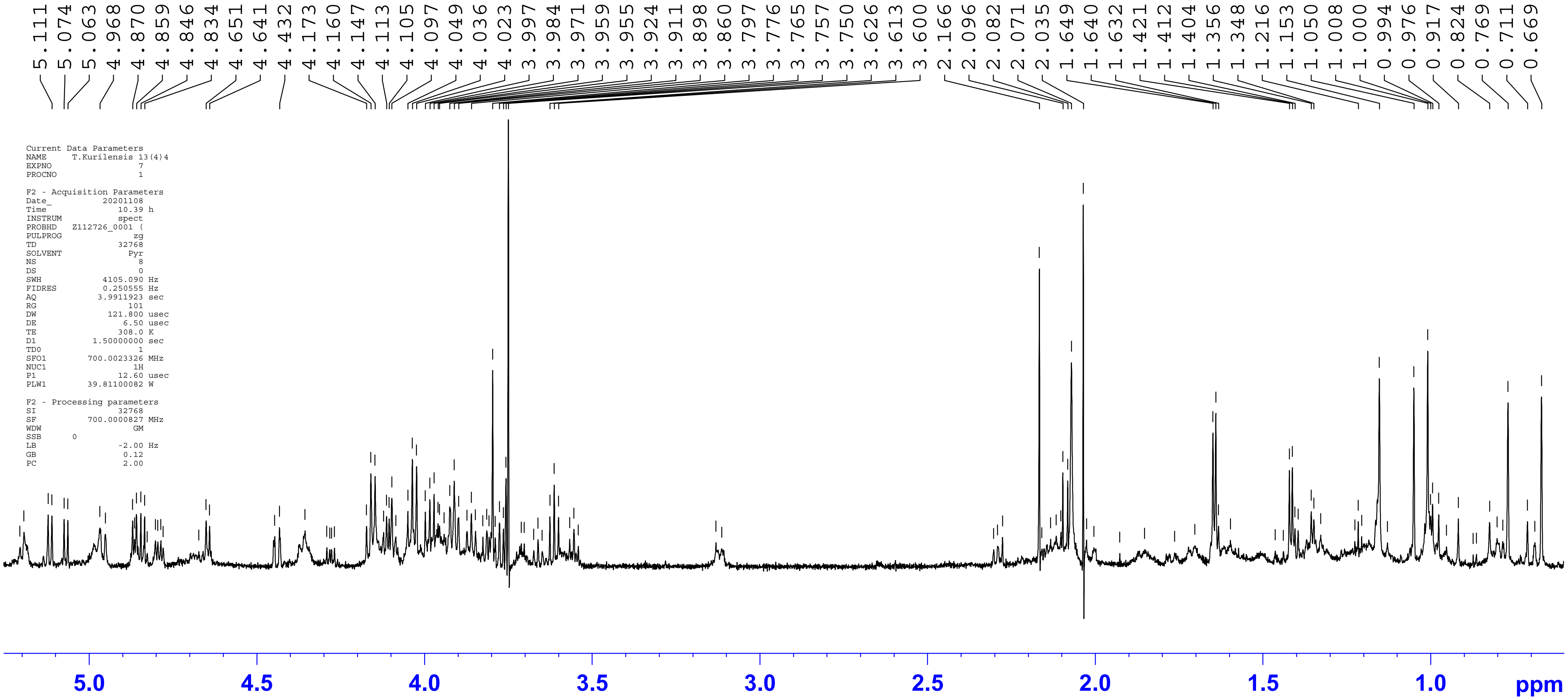

Figure S49. The ^{1}H NMR (700.00 MHz) spectrum of kuriloside J (**7**) in C_5D_5N/D_2O (4/1)

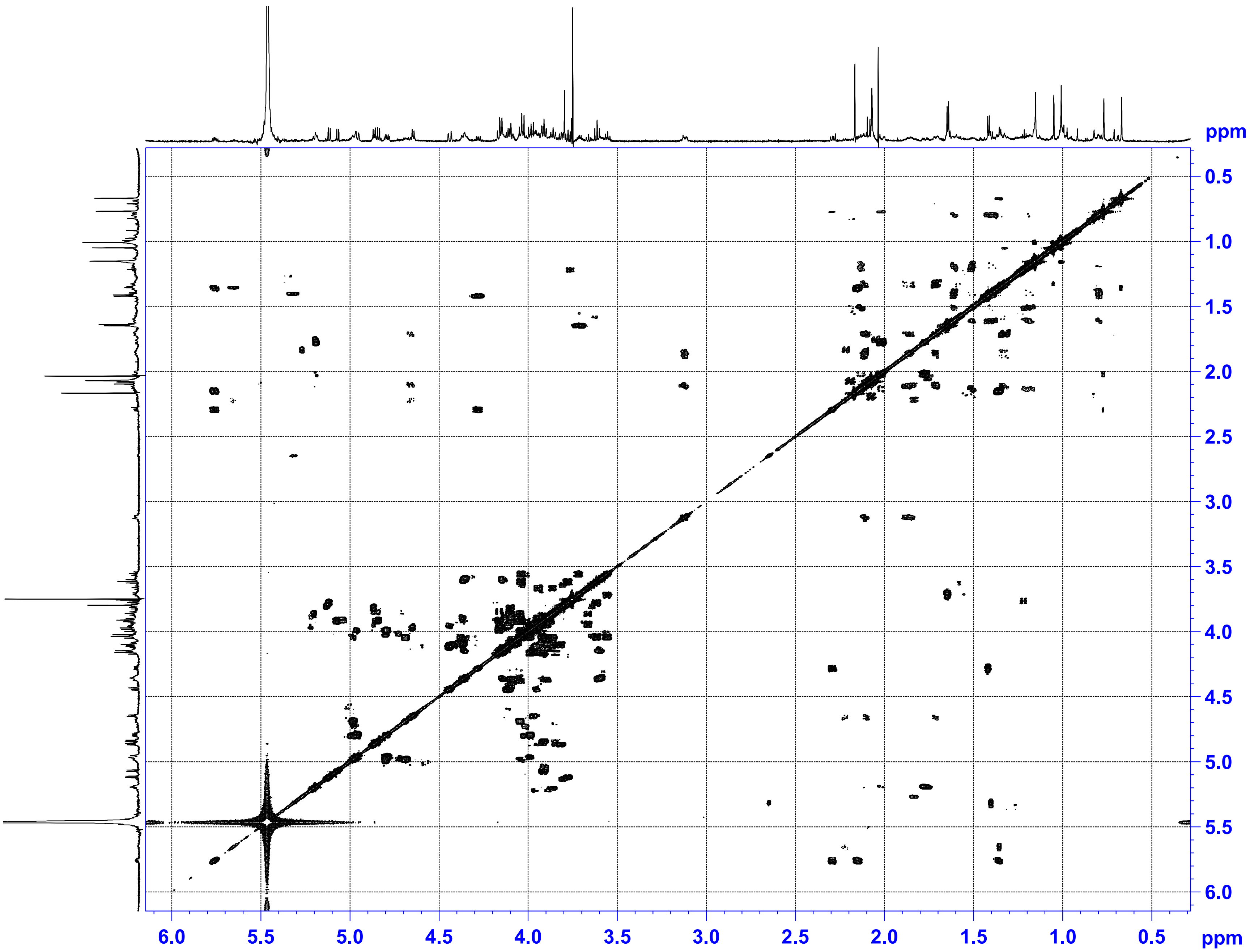

Figure S50. The COSY (700.00 MHz) spectrum of kuriloside J (**7**) in C_5D_5N/D_2O (4/1)

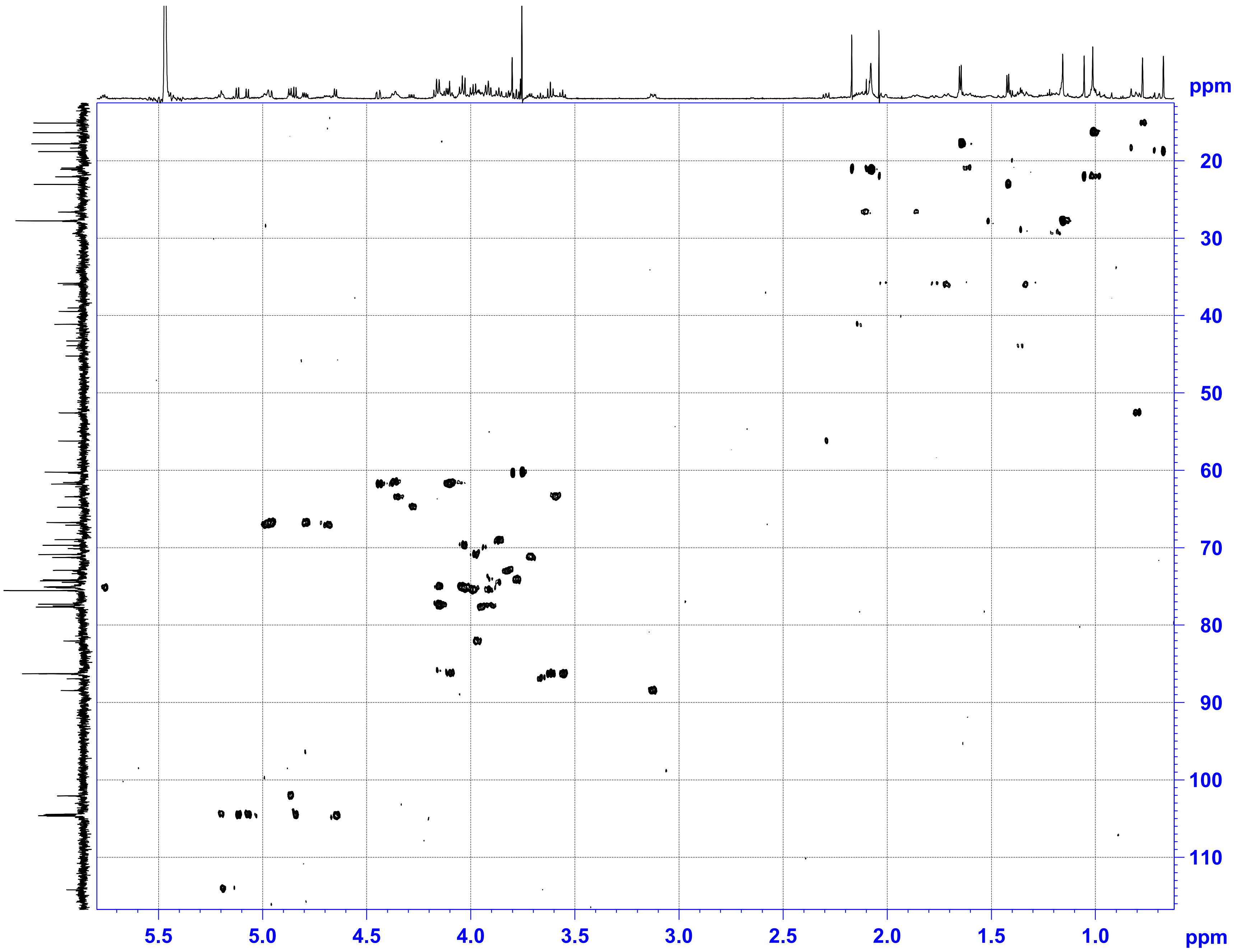

Figure S51. The HSQC (700.00 MHz) spectrum of kuriloside J (**7**) in C_5D_5N/D_2O (4/1)

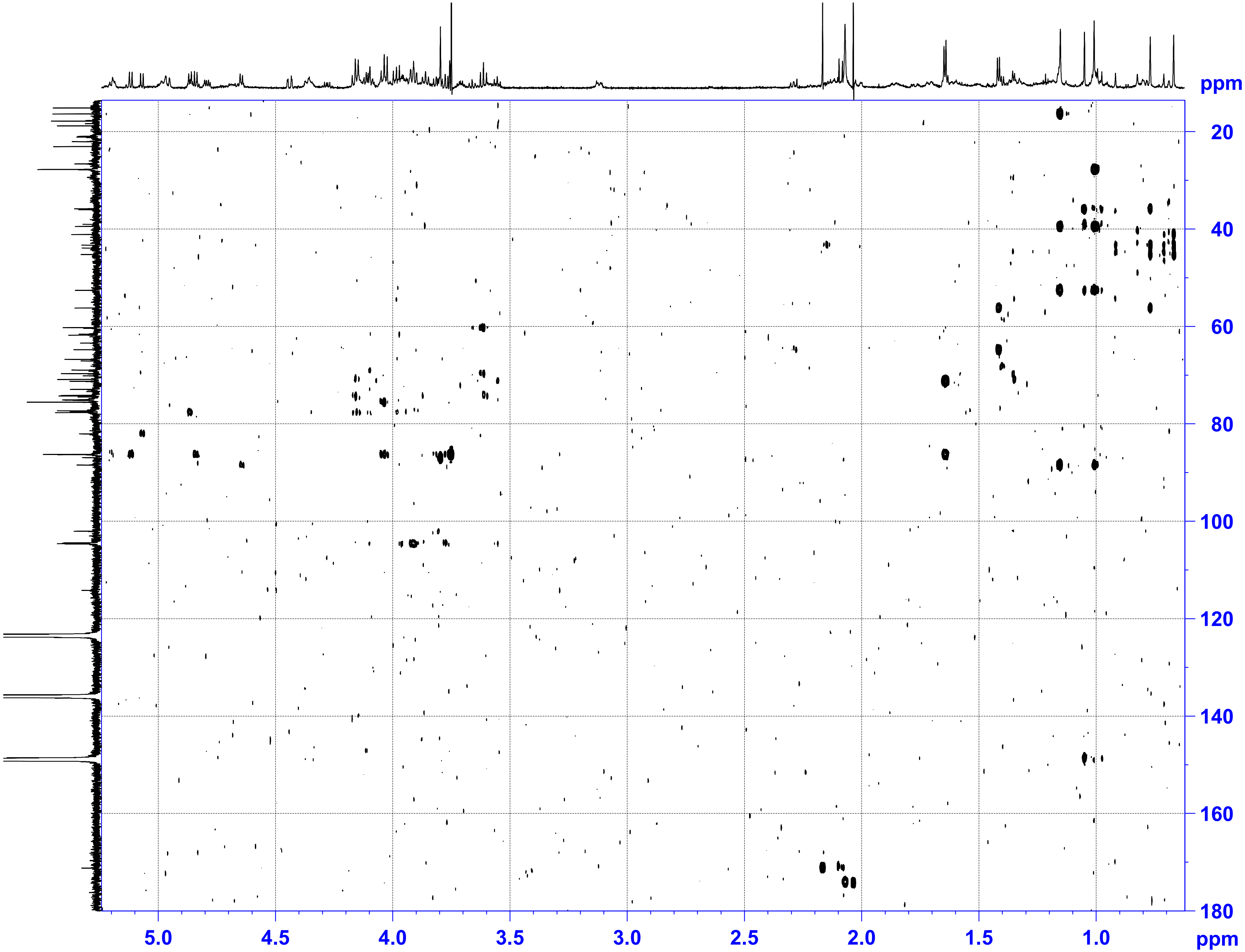

Figure S52. The HMBC (700.00 MHz) spectrum of kuriloside J (**7**) in C_5D_5N/D_2O (4/1)

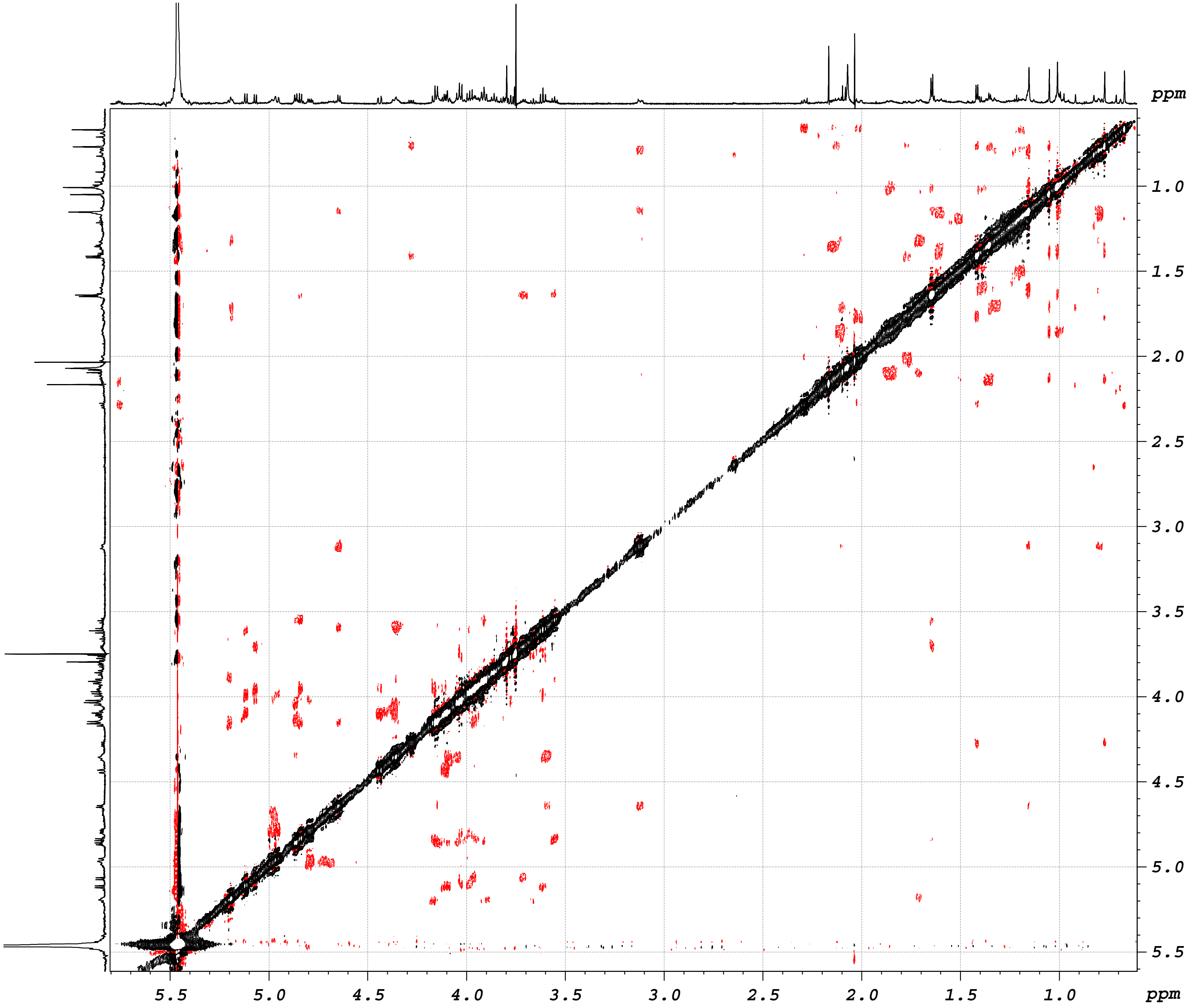

Figure S53. The ROESY (700.00 MHz) spectrum of kuriloside J (**7**) in C_5D_5N/D_2O (4/1)

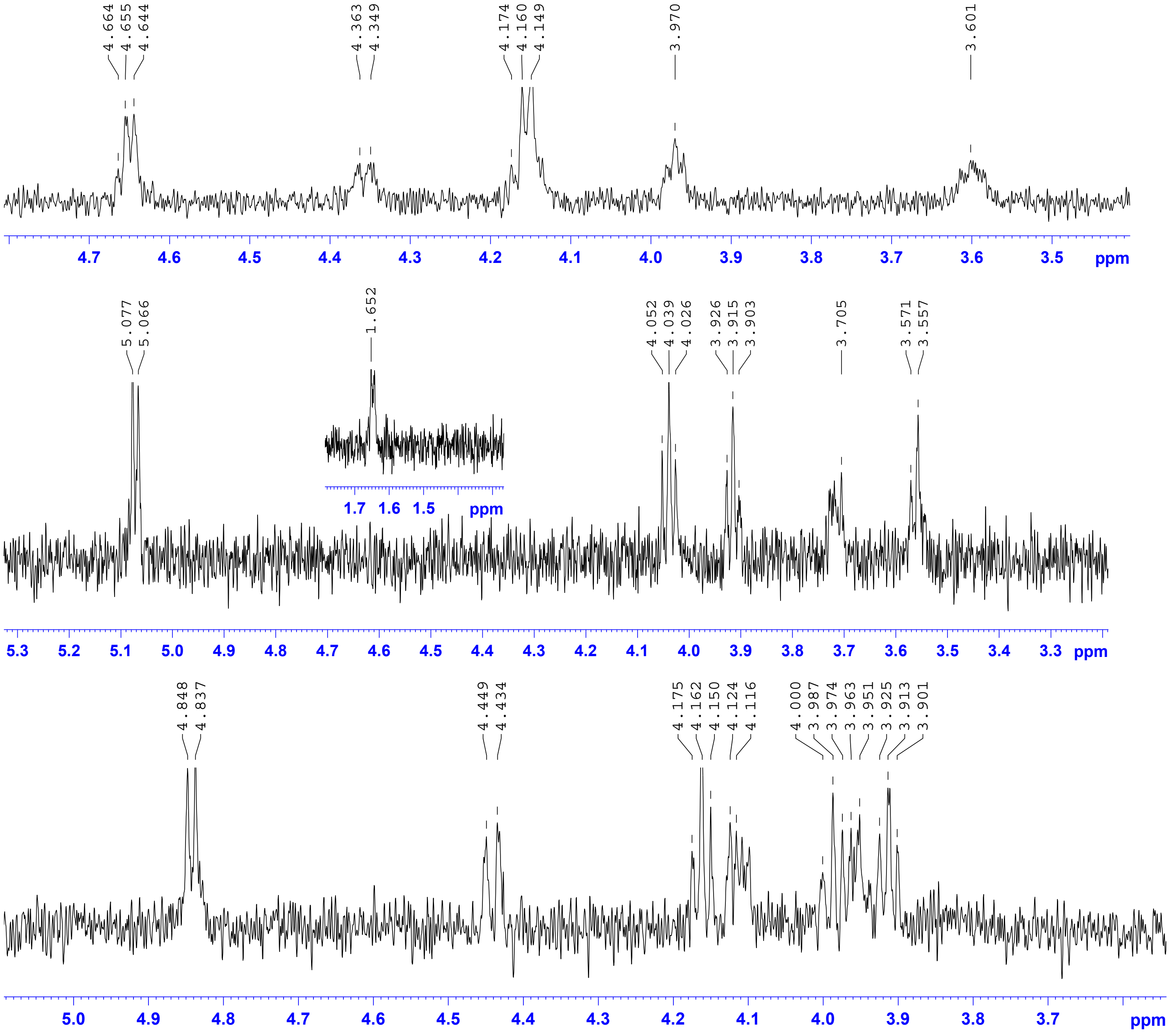

Figure S54. 1 D TOCSY (700.00 MHz) spectra of XyloseI, QuinovoseII and GlucoseIII of kuriloside J (**7**) in C_5D_5N/D_2O (4/1)

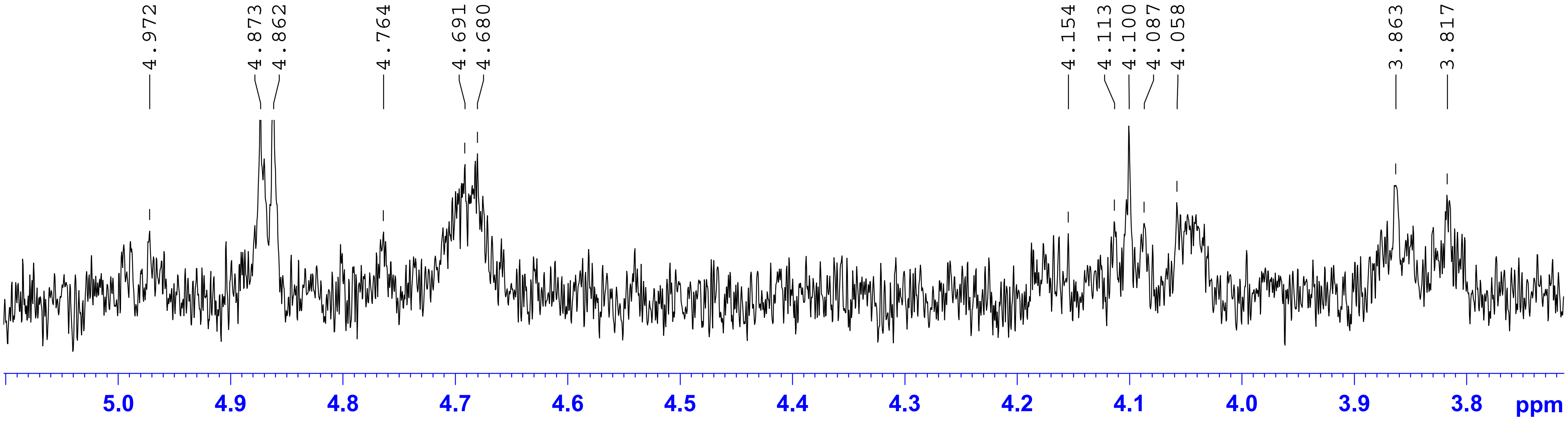

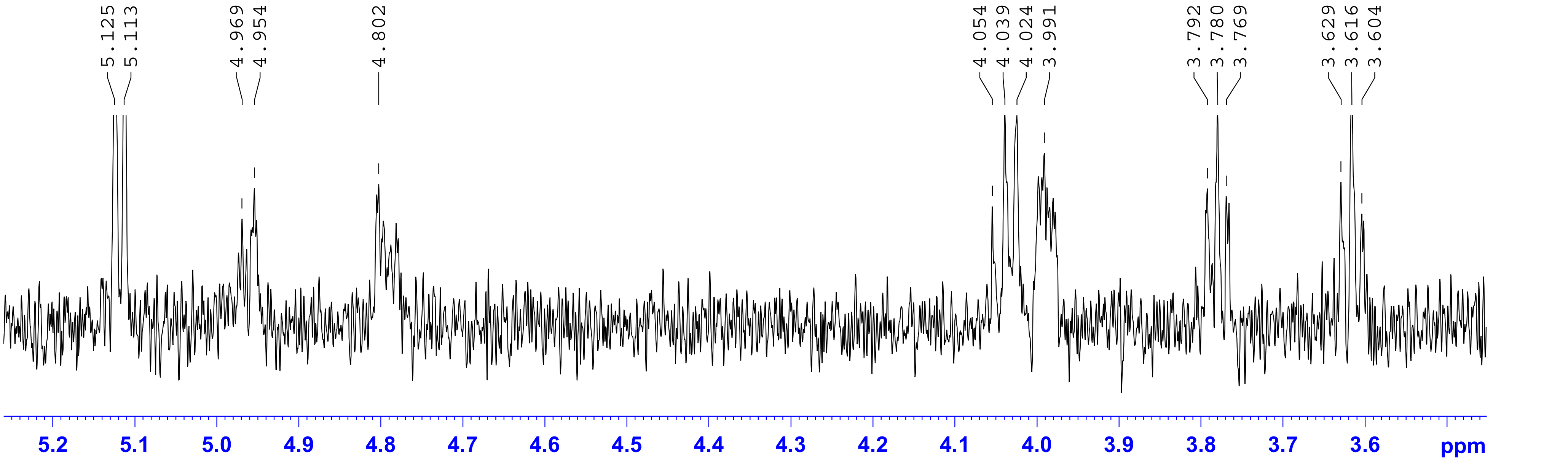

Figure S55. 1 D TOCSY (700.00 MHz) spectra of GlcIV and MeGlcV of kuriloside J (**7**) in C_5D_5N/D_2O (4/1)

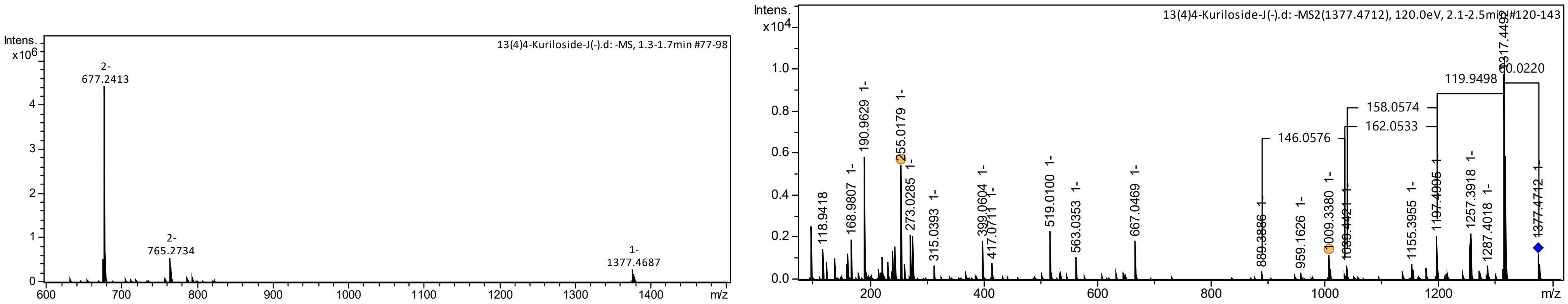

Figure S56. HR-ESI-MS and ESI-MS/MS spectra of kuriloside J (**7**)

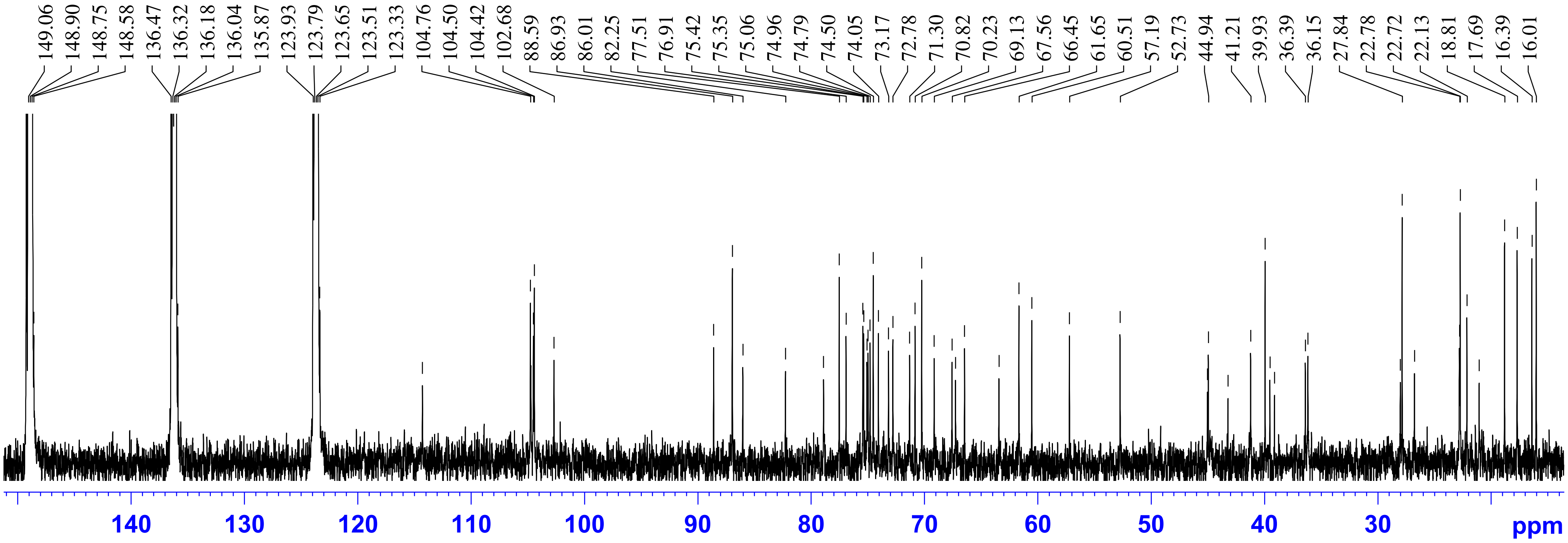

Figure S57. The ^{13}C NMR (176.03 MHz) spectrum of kuriloside K (**8**) in C_5D_5N/D_2O (4/1)

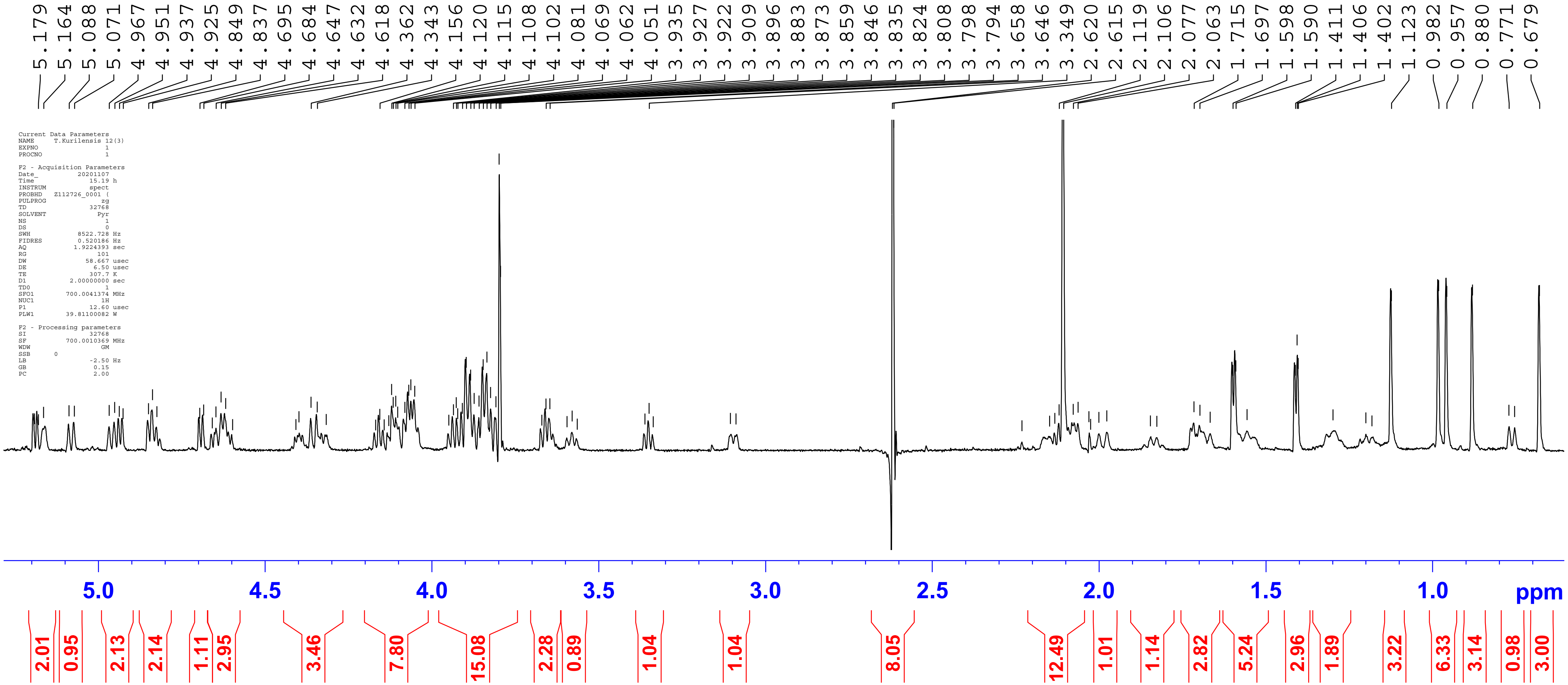

Figure S58. The ^{1}H NMR (700.00 MHz) spectrum of kuriloside K (**8**) in C_5D_5N/D_2O (4/1)

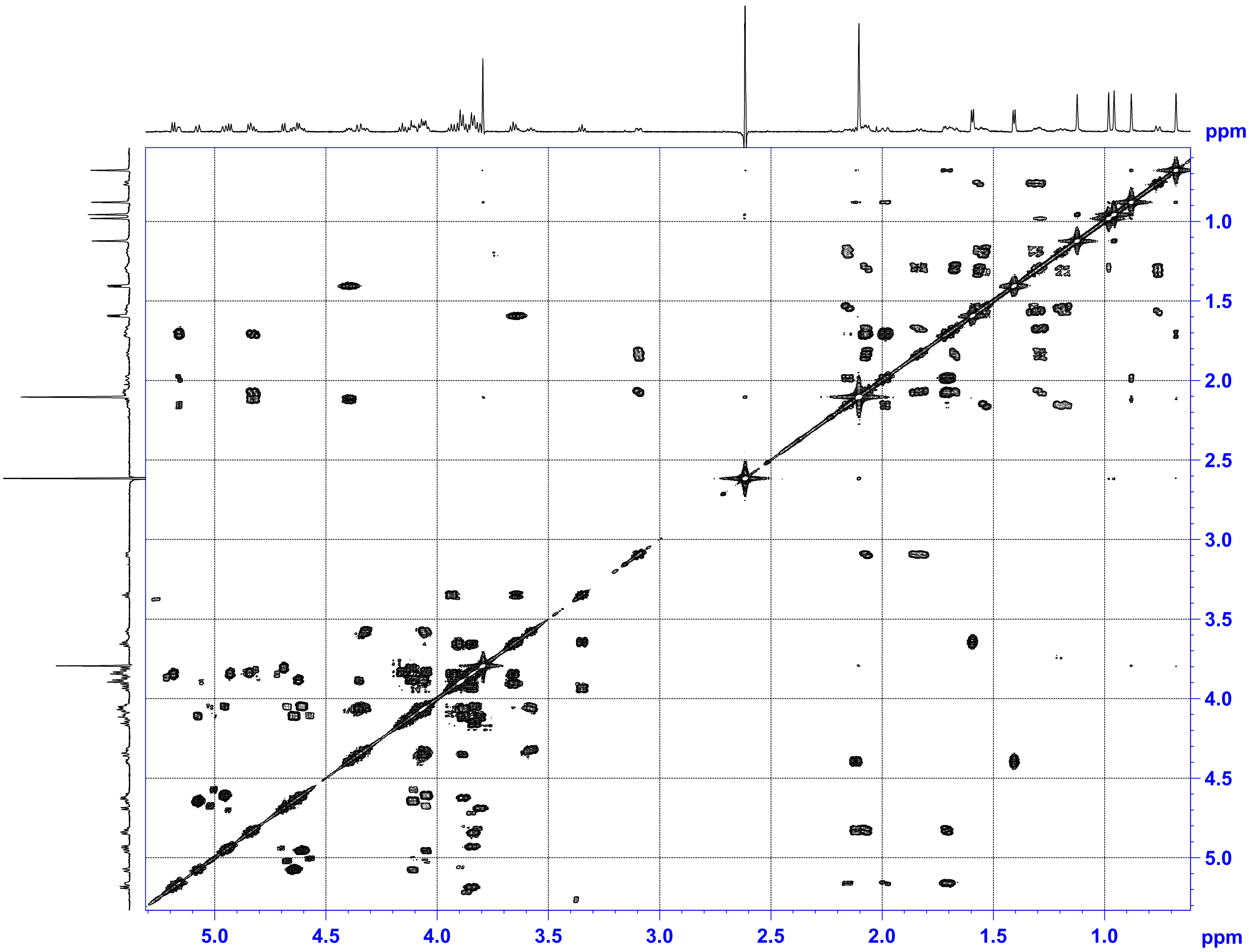

Figure S59. The COSY (700.00 MHz) spectrum of kuriloside K (**8**) in C_5D_5N/D_2O (4/1)

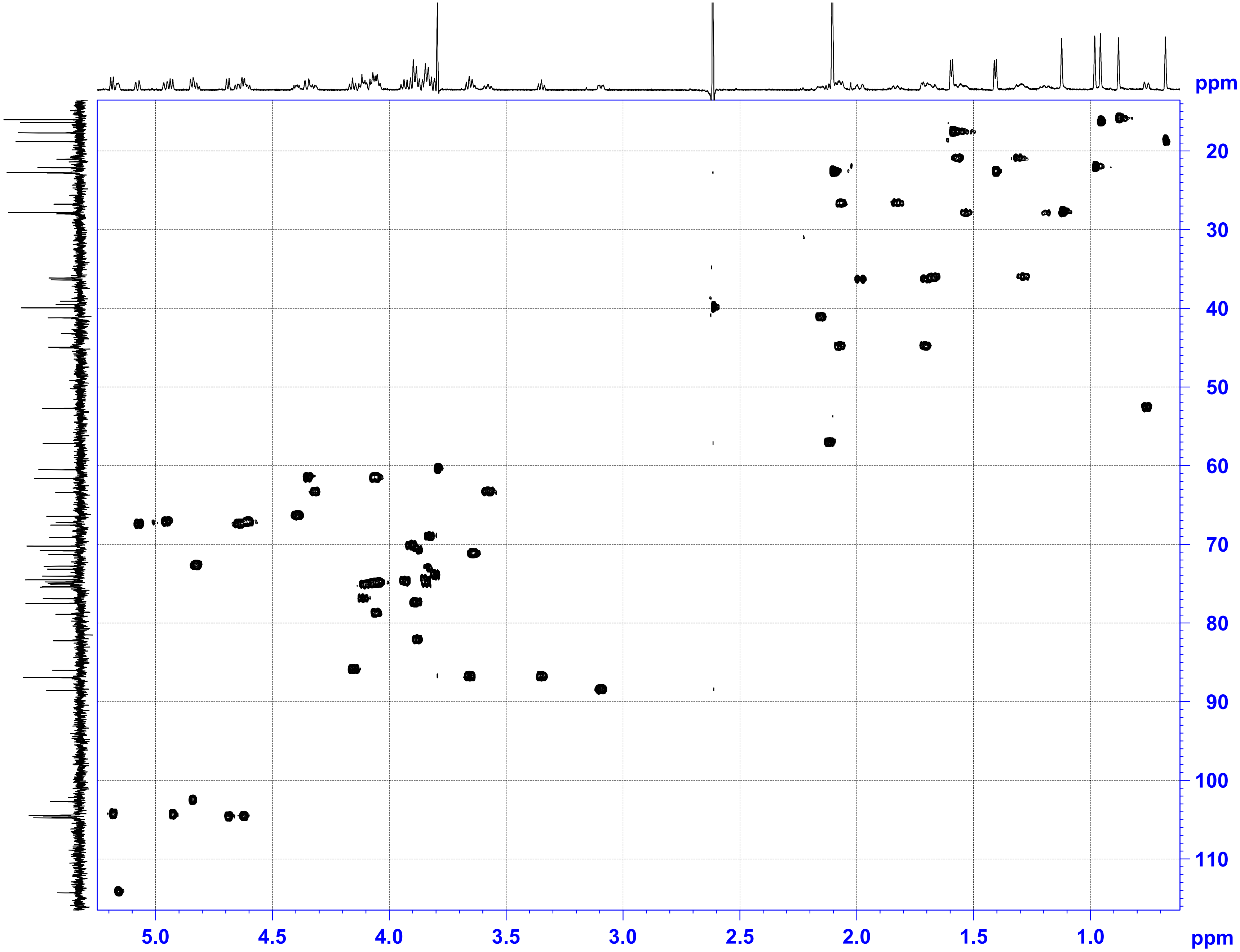

Figure S60. The HSQC (700.00 MHz) spectrum of kuriloside K (8) in C_5D_5N/D_2O (4/1)

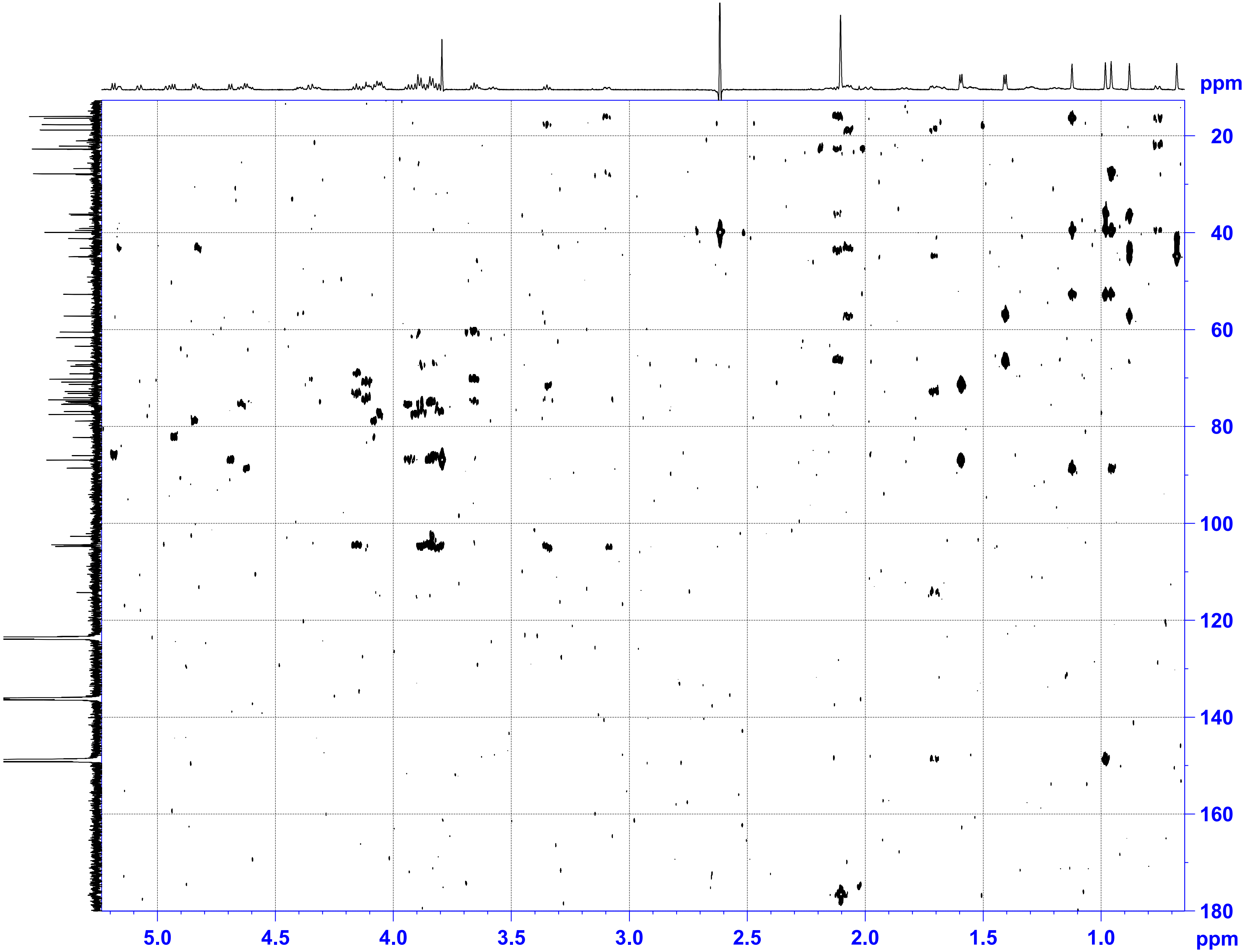

Figure S61. The HMBC (700.00 MHz) spectrum of kuriloside K (**8**) in C_5D_5N/D_2O (4/1)

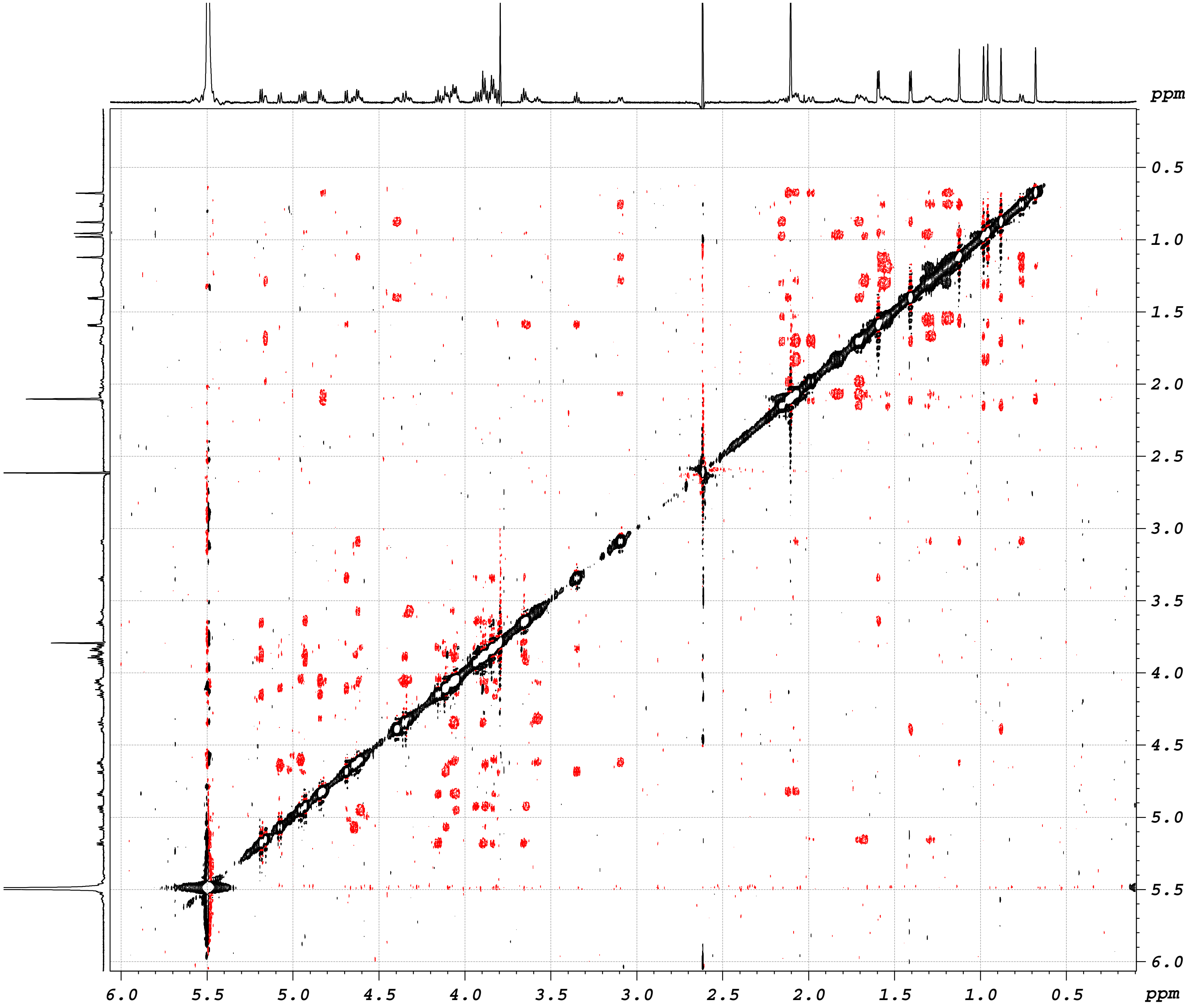

Figure S62. The ROESY (700.00 MHz) spectrum of kuriloside K (**8**) in C_5D_5N/D_2O (4/1)

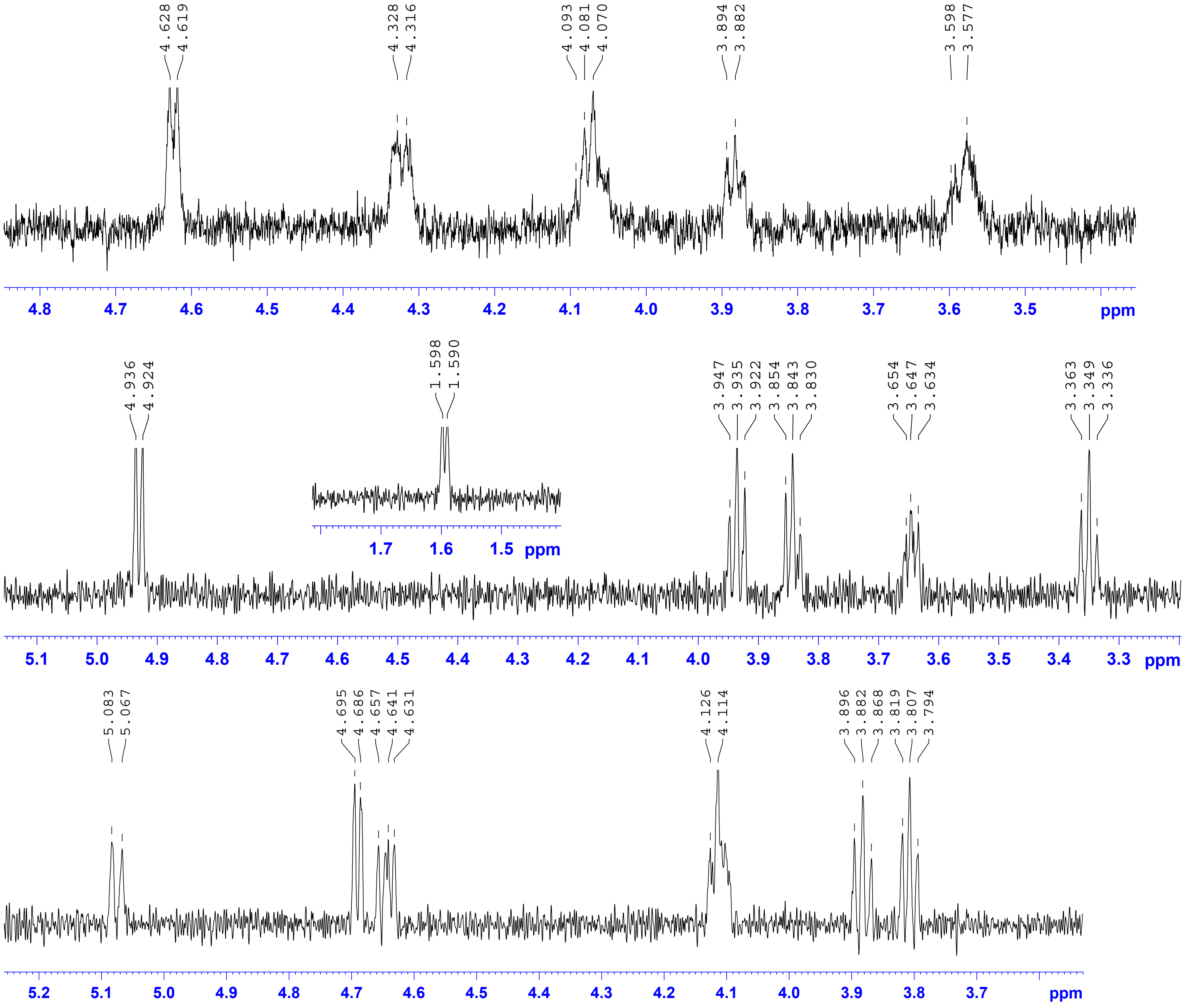

Figure S63. 1 D TOCSY (700.00 MHz) spectra of XyloseI, QuinovoseII and GlucoseIII of kuriloside K (**8**) in C_5D_5N/D_2O (4/1)

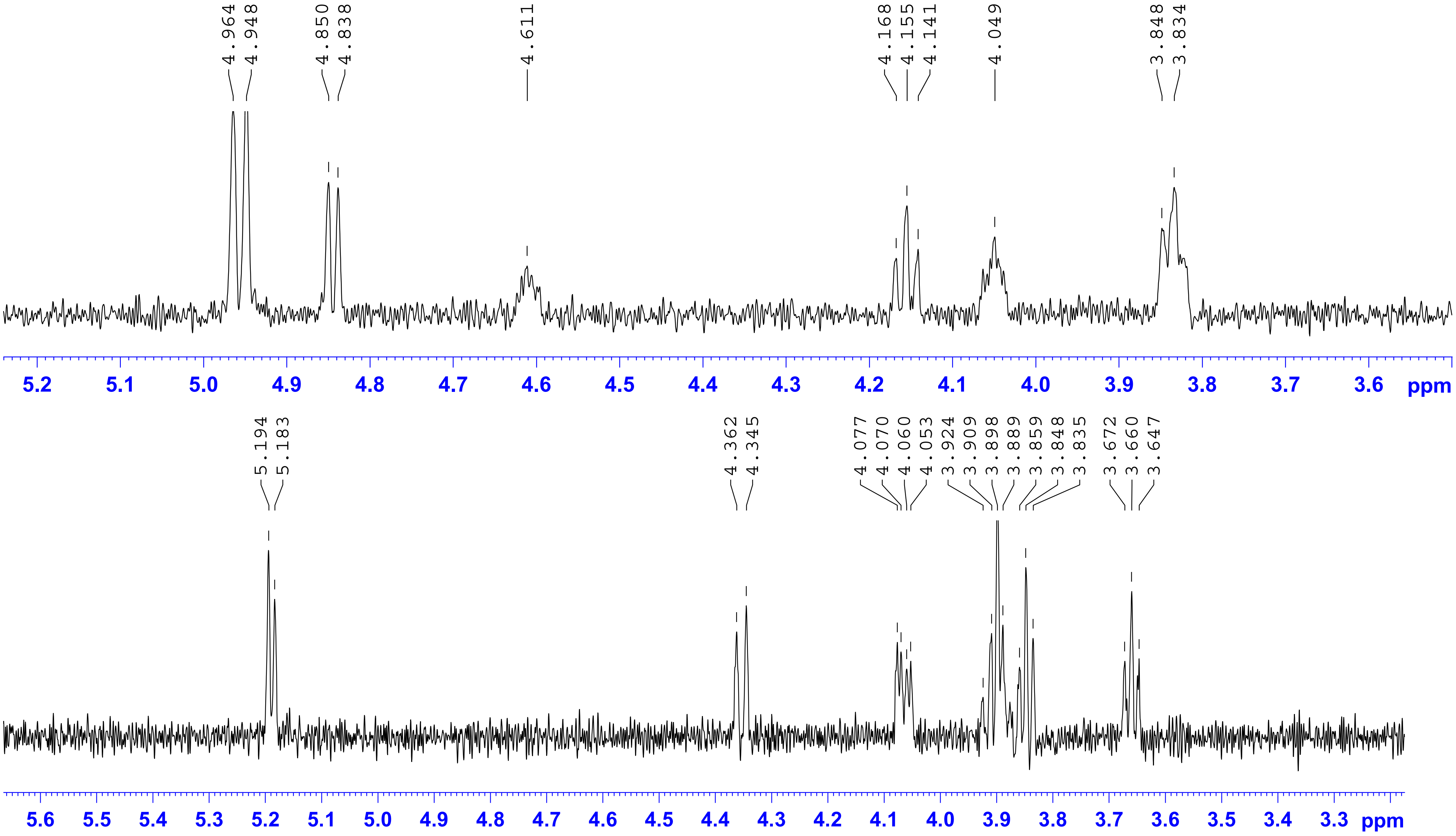

Figure S64. 1 D TOCSY (700.00 MHz) spectra of GlcIV and MeGlcV of kuriloside K (**8**) in C_5D_5N/D_2O (4/1)

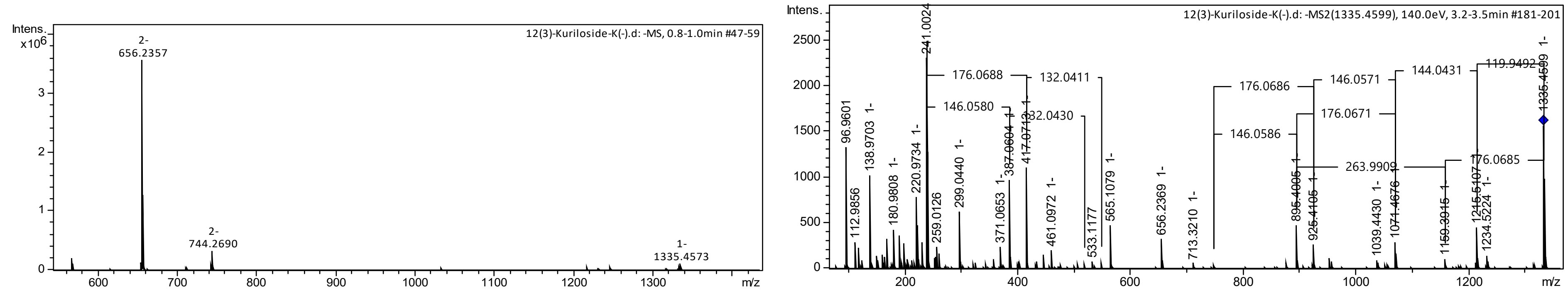

Figure S65. HR-ESI-MS and ESI-MS/MS spectra of kuriloside K (**8**)

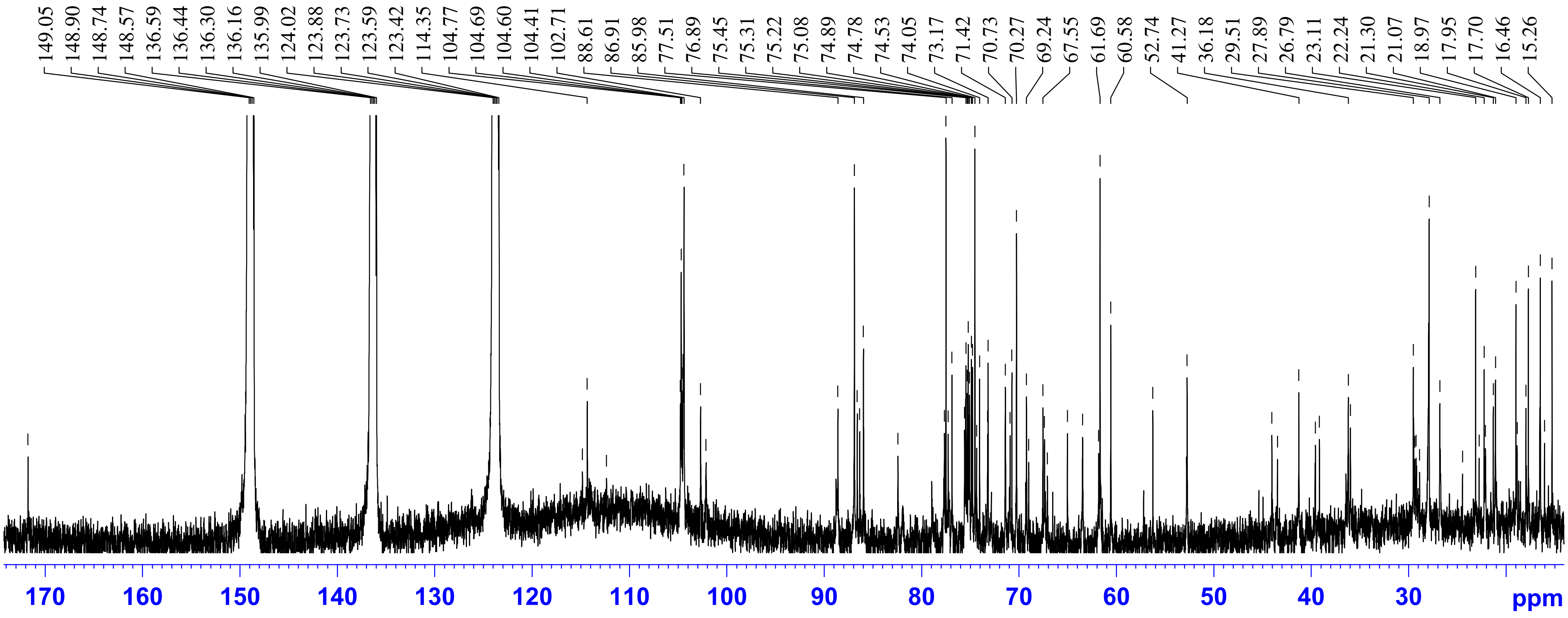

Figure S66. The ^{13}C NMR (176.03 MHz) spectrum of kuriloside K_1 (**9**) in C_5D_5N/D_2O (4/1)

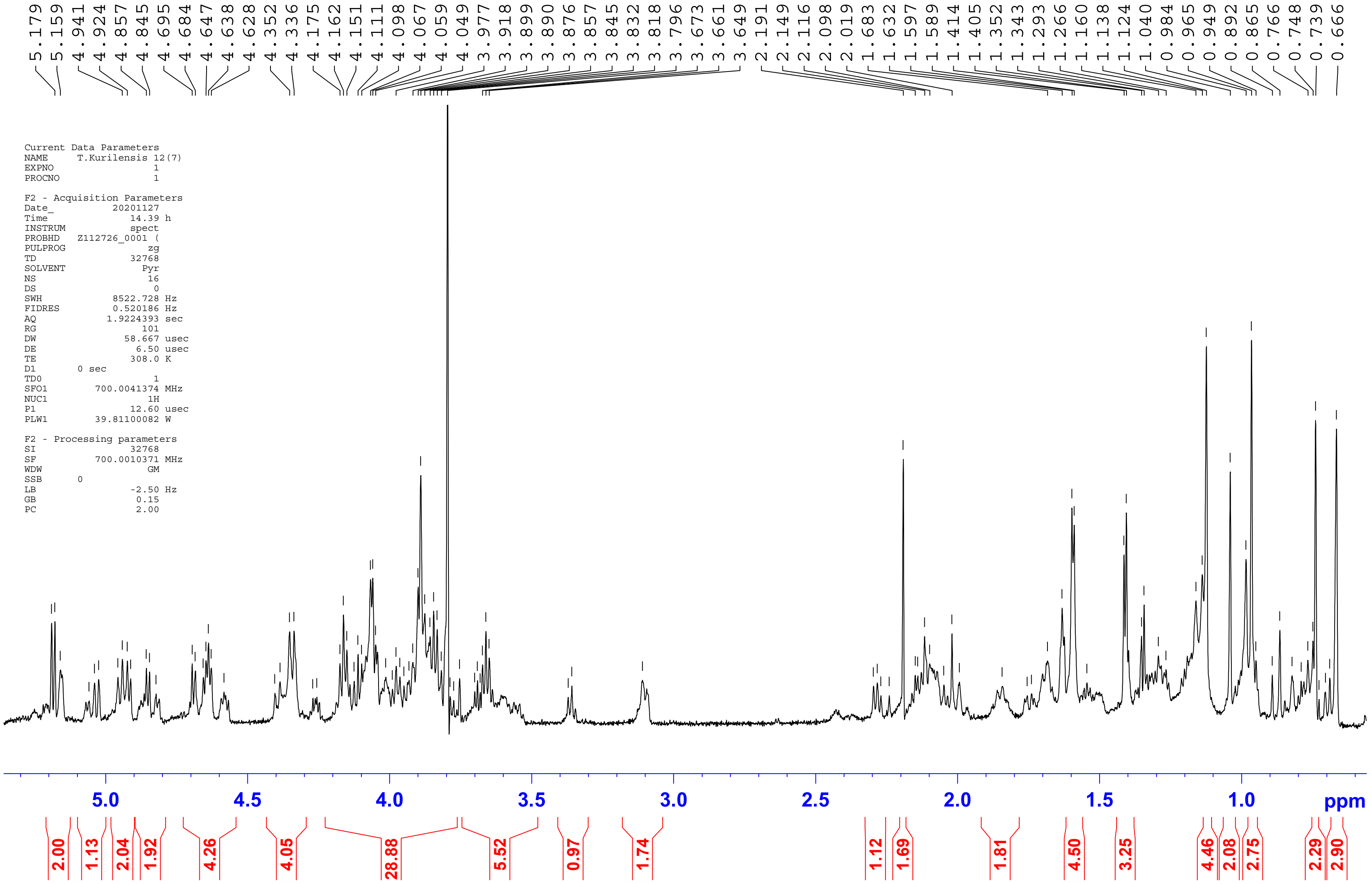

Figure S67. The ^{1}H NMR (700.00 MHz) spectrum of kuriloside K_1 (**9**) in C_5D_5N/D_2O (4/1)

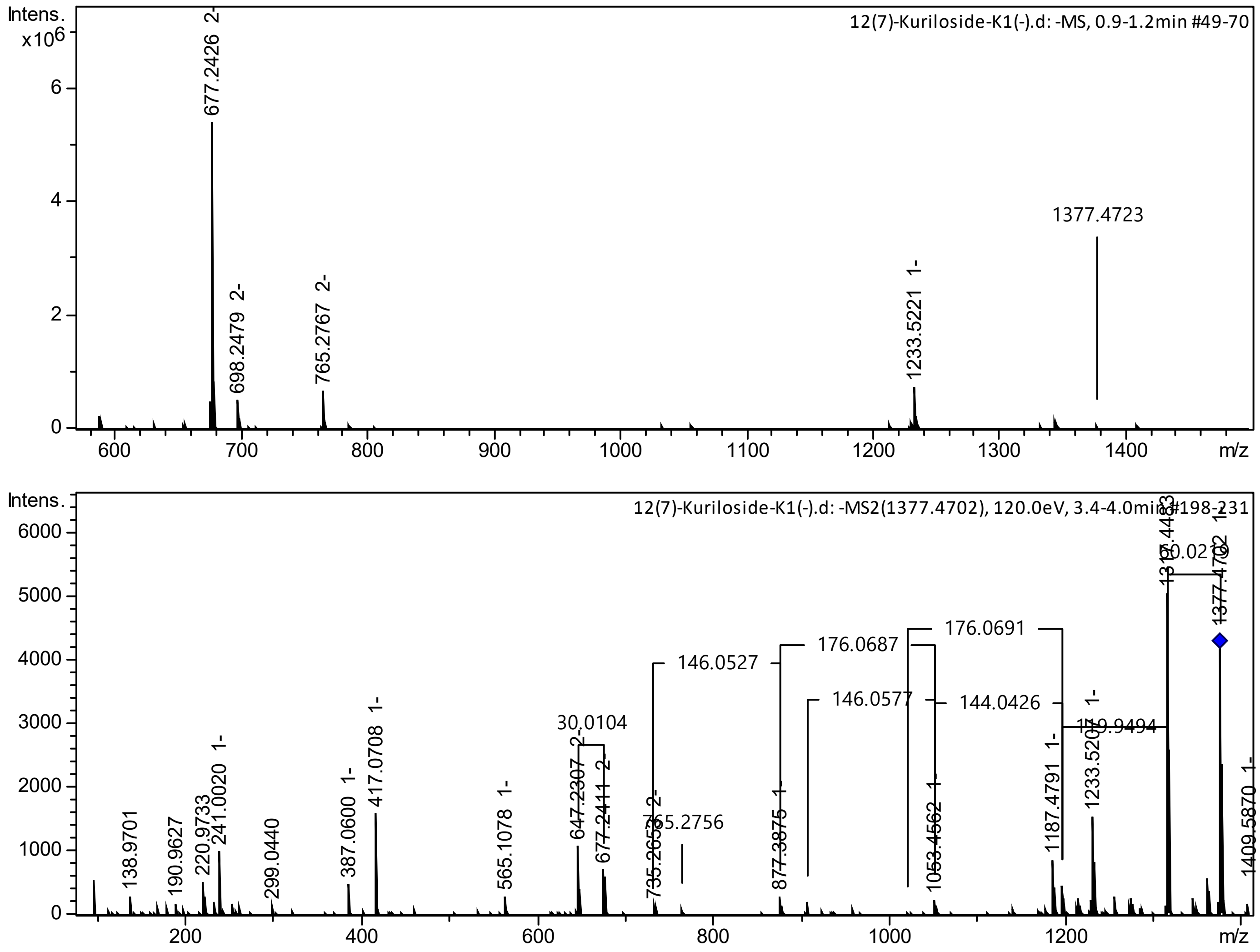

Figure S68. HR-ESI-MS and ESI-MS/MS spectra of kuriloside K1 (**9**)

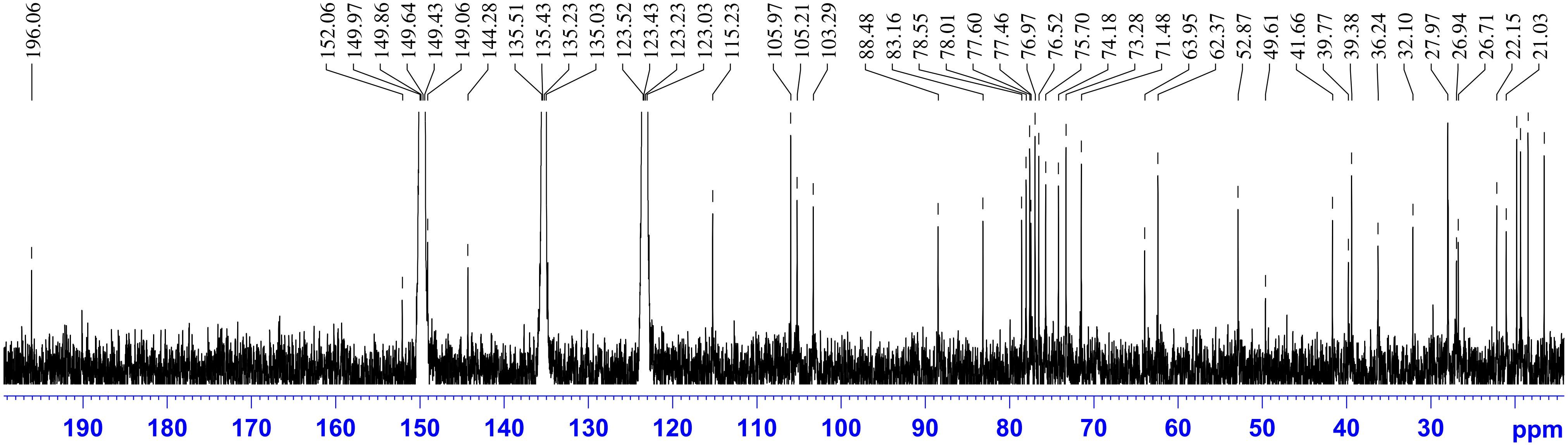

Figure S69. The ^{13}C NMR (176.03 MHz) spectrum of DS-kuriloside L (**10**) in C_5D_5N

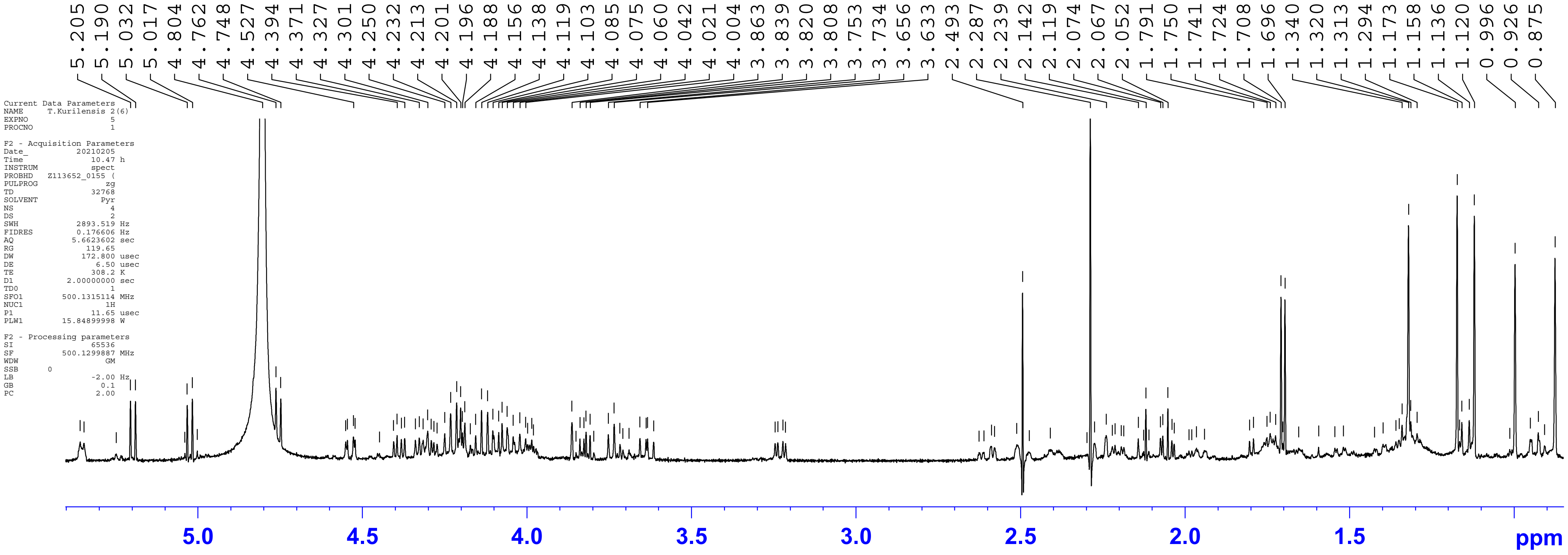

Figure S70. The ^{1}H NMR (700.00 MHz) spectrum of DS-kuriloside L (**10**) in C_5D_5N

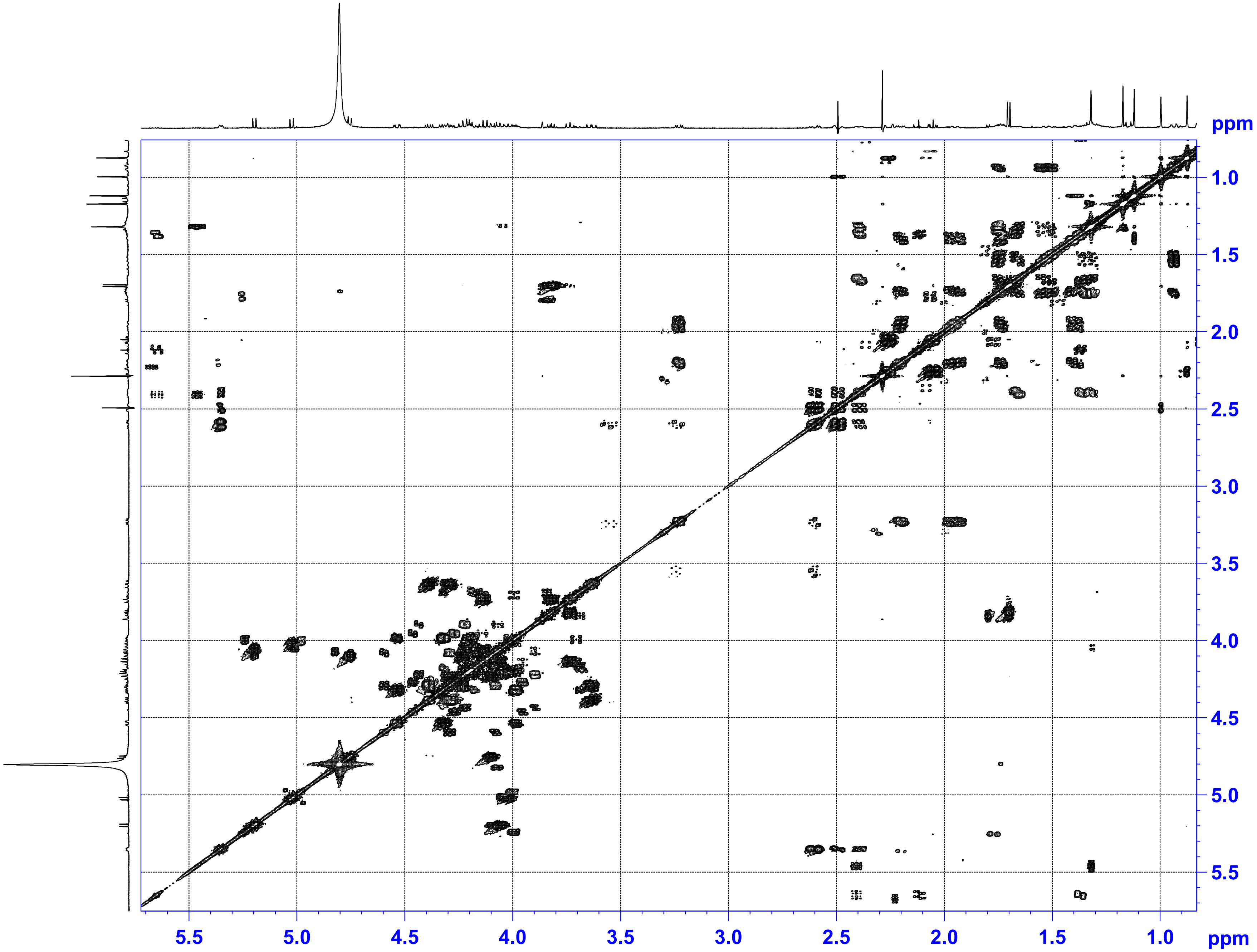

Figure S71. The COSY (700.00 MHz) spectrum of DS-kuriloside L (**10**) in C_5D_5N

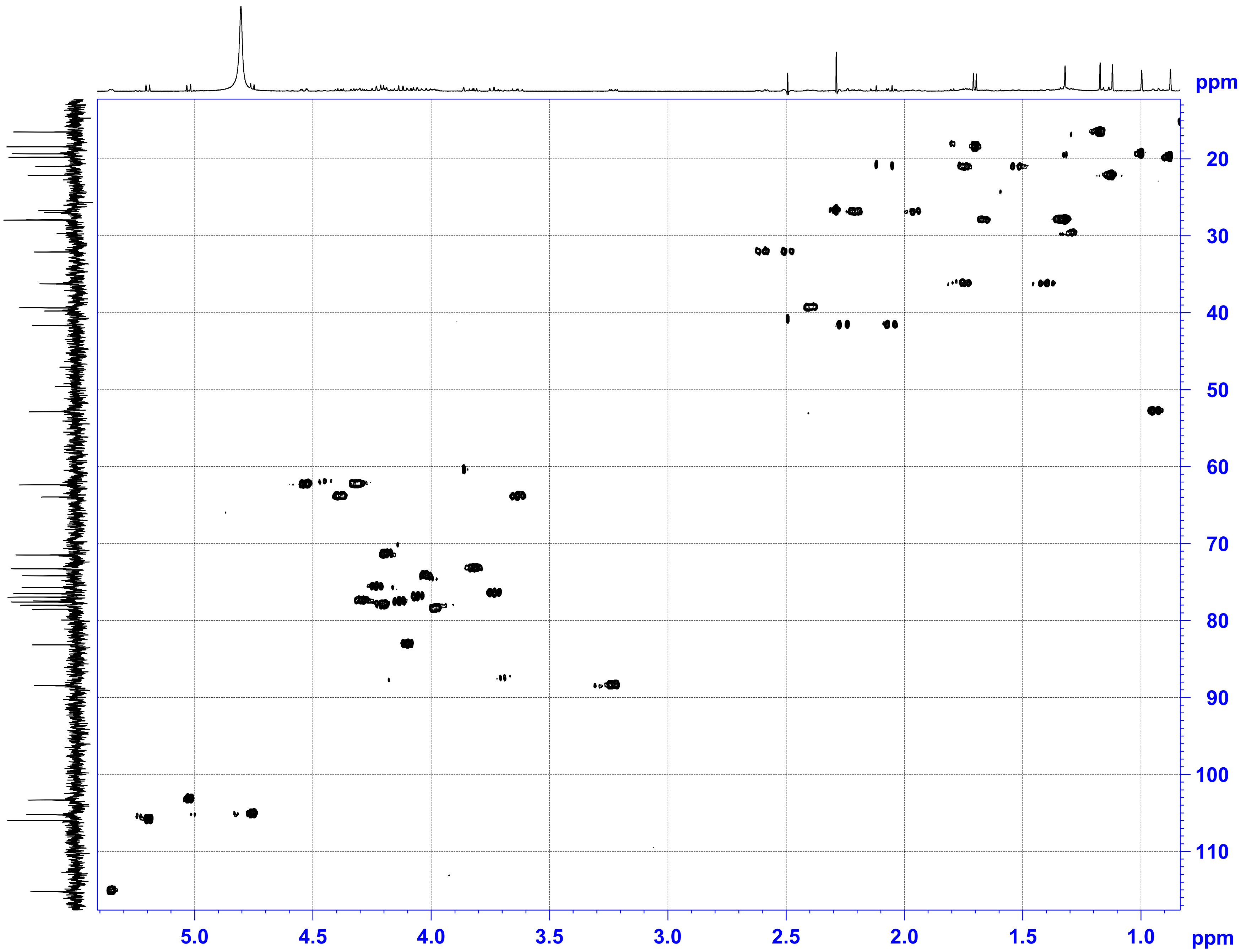

Figure S72. The HSQC (700.00 MHz) spectrum of DS-kuriloside L (**10**) in C_5D_5N

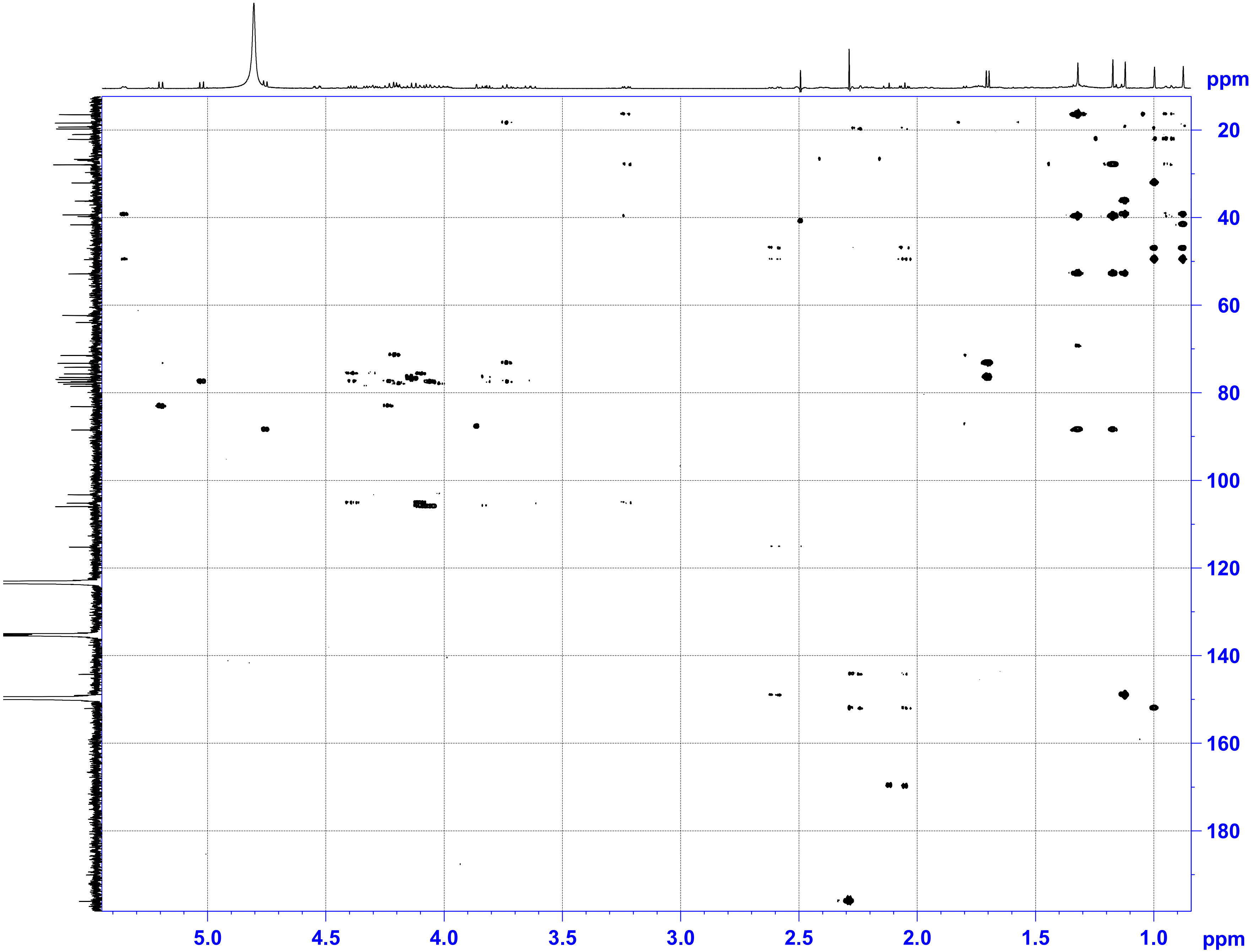

Figure S73. The HMBC (700.00 MHz) spectrum of DS-kuriloside L (**10**) in C_5D_5N

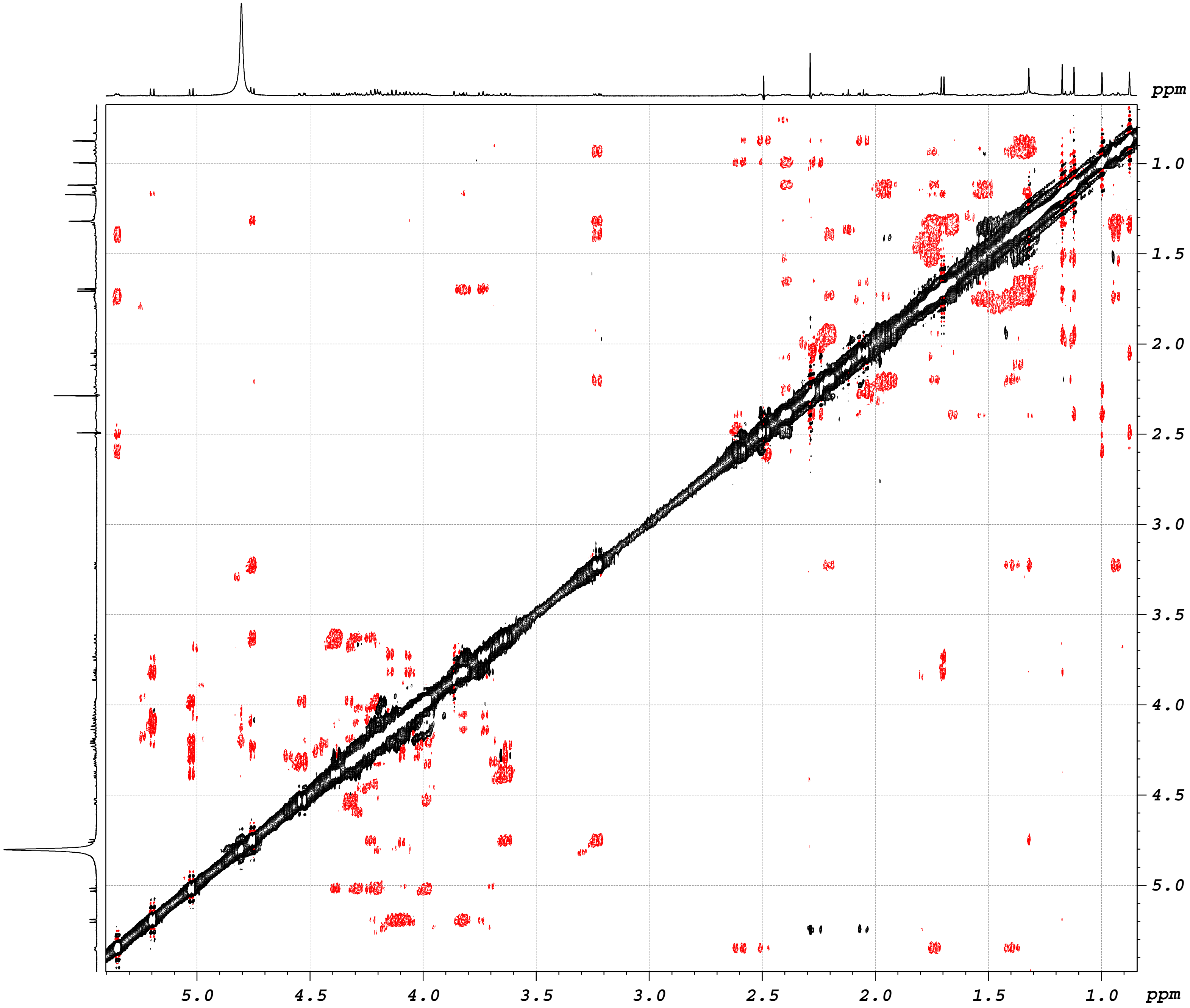

Figure S74. The ROESY (700.00 MHz) spectrum of DS-kuriloside L (**10**) in C_5D_5N

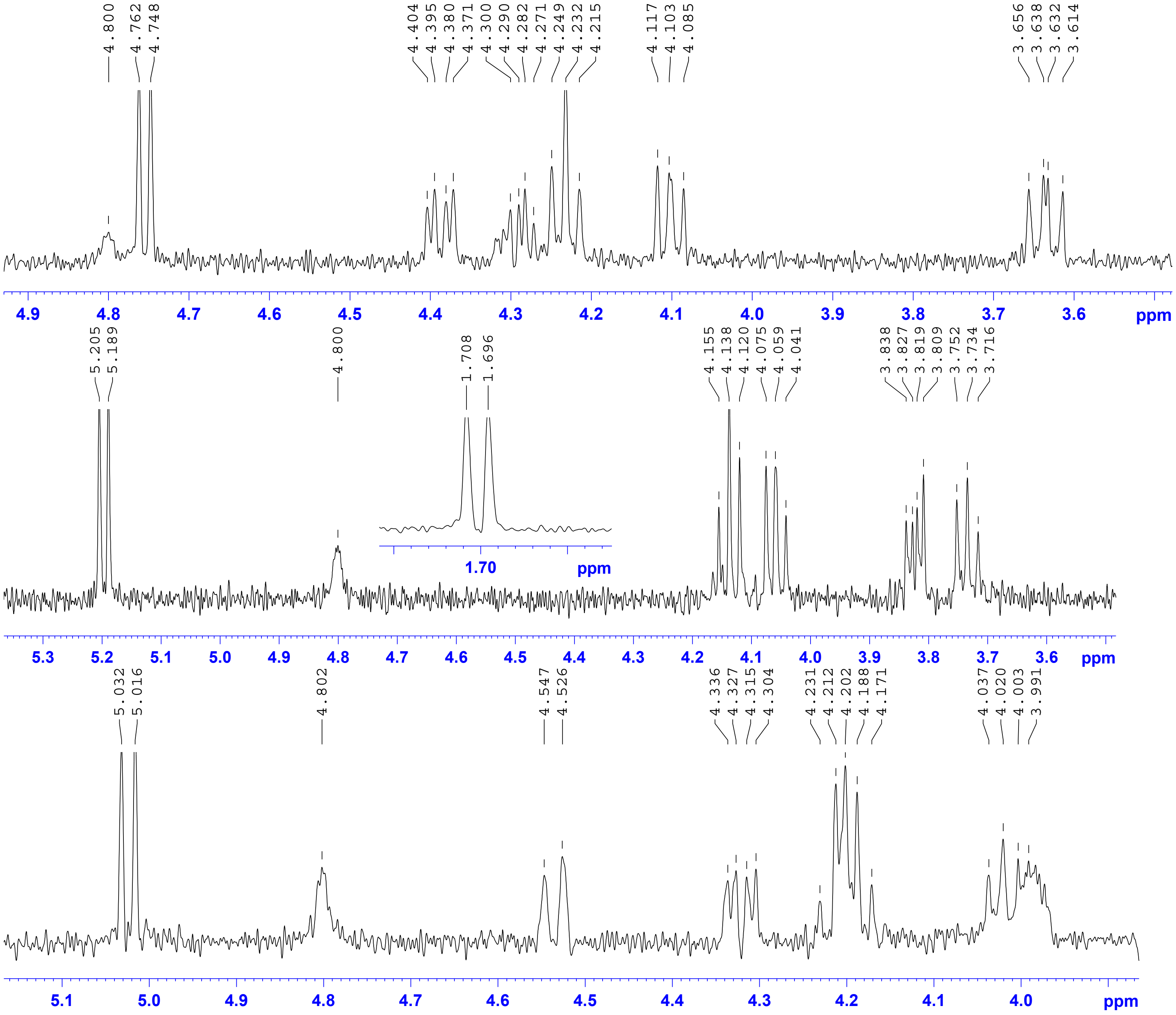

Figure S75. 1 D TOCSY (700.00 MHz) spectra of XyloseI, QuinovoseII and GlucoseIII of DS-kuriloside L (**10**) in C_5D_5N

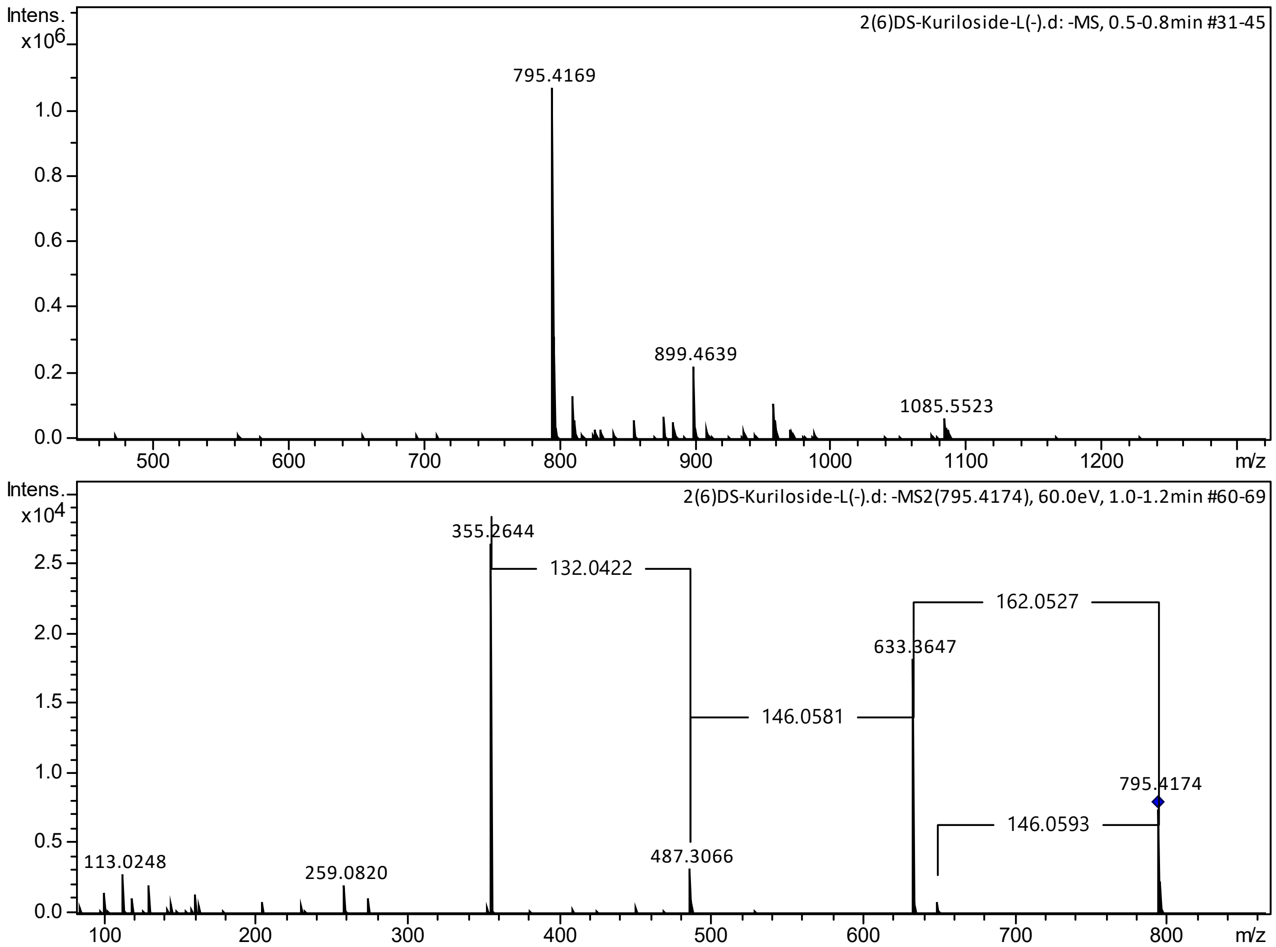

Figure S76. HR-ESI-MS (−) and ESI-MS/MS spectra of DS-kuriloside L (**10**).

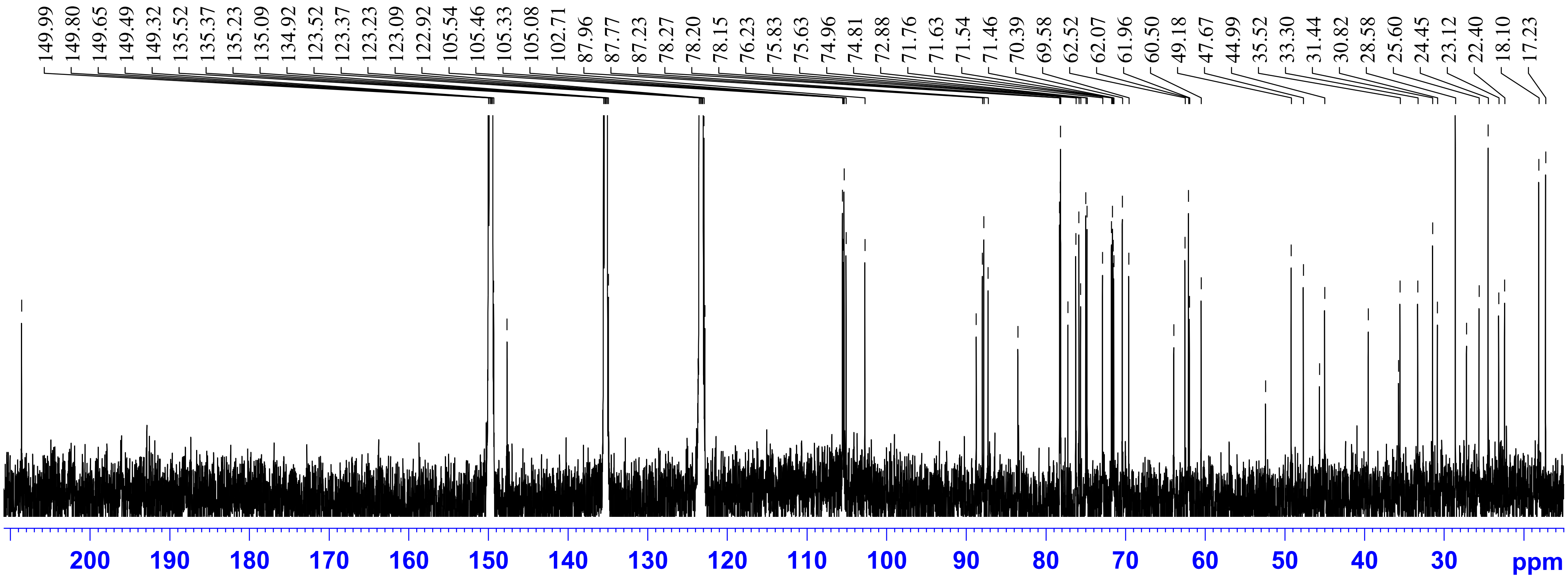

Figure S77. The ^{13}C NMR (176.03 MHz) spectrum of DS-kuriloside M (**11**) in C_5D_5N

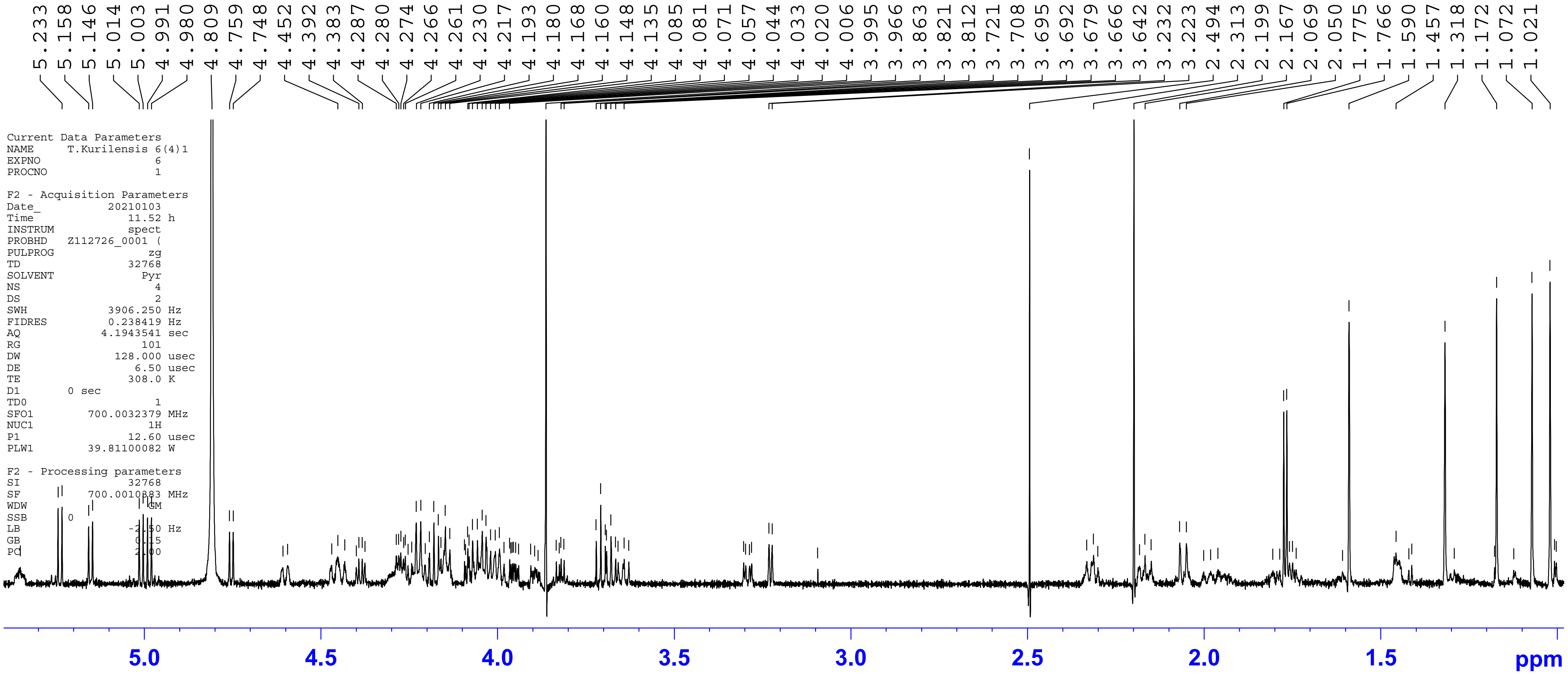

Figure S78. The ^{1}H NMR (700.00 MHz) spectrum of DS-kuriloside M (**11**) in C_5D_5N

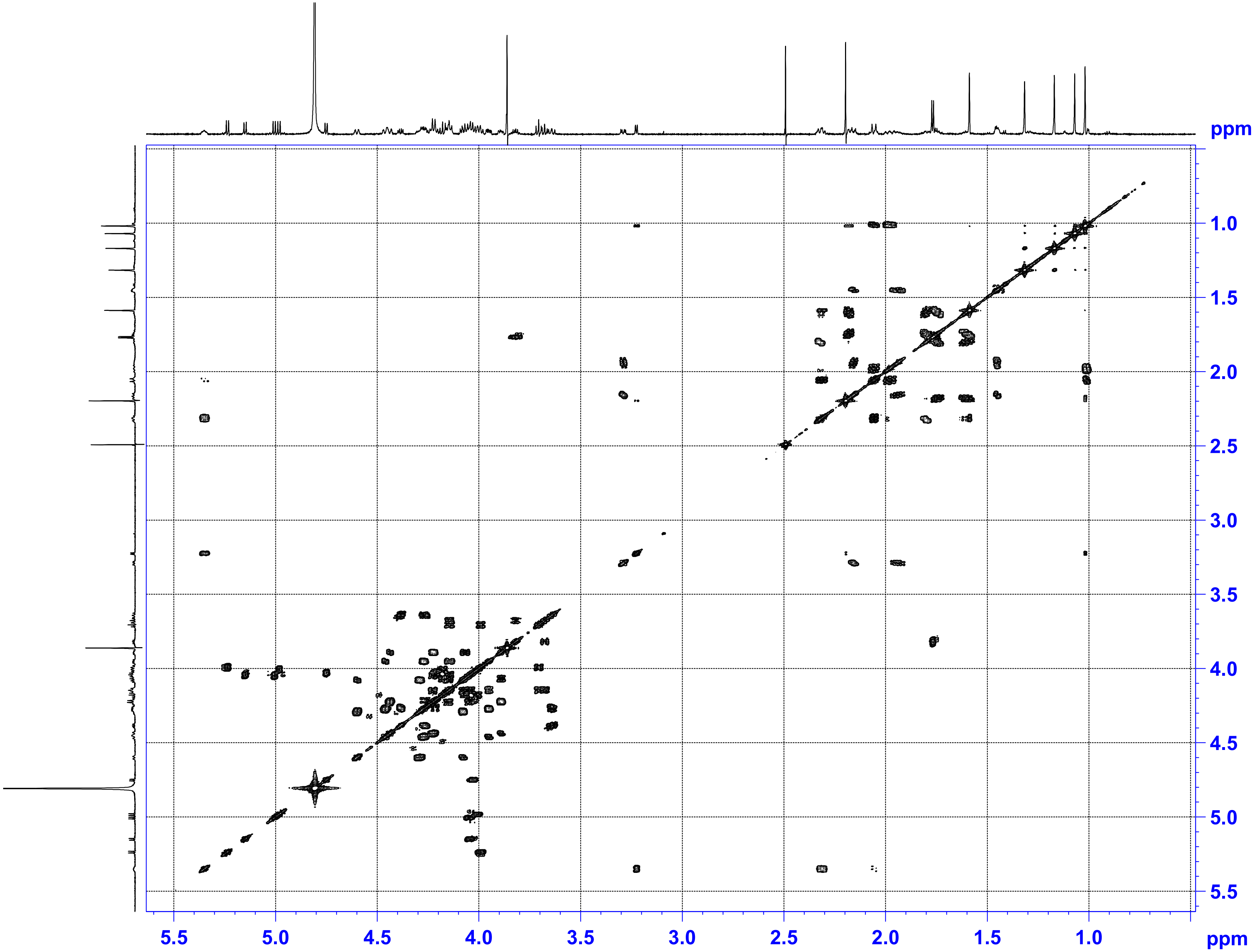

Figure S79. The COSY (700.00 MHz) spectrum of DS-kuriloside M (**11**) in C_5D_5N

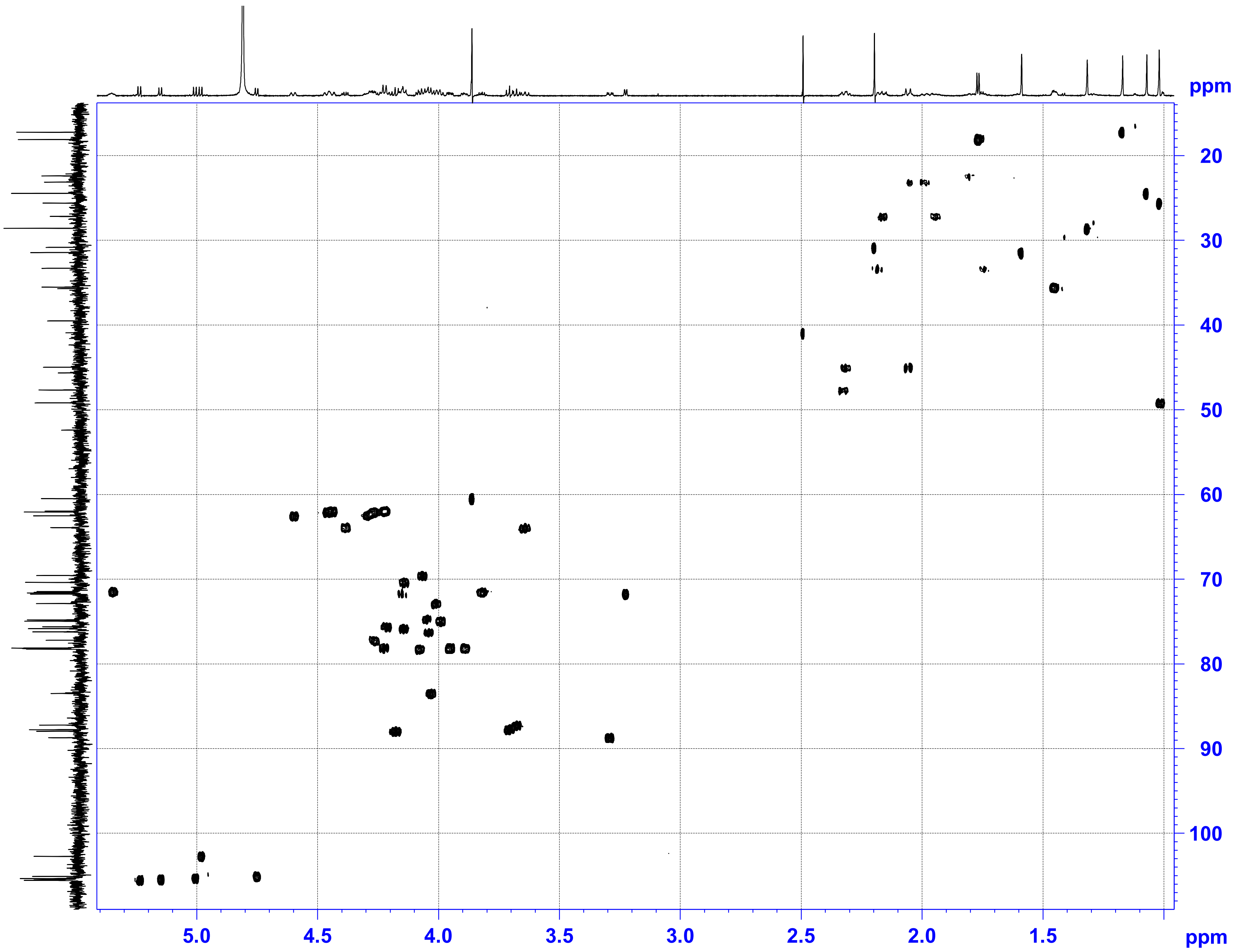

Figure S80. The HSQC (700.00 MHz) spectrum of DS-kuriloside M (**11**) in C_5D_5N

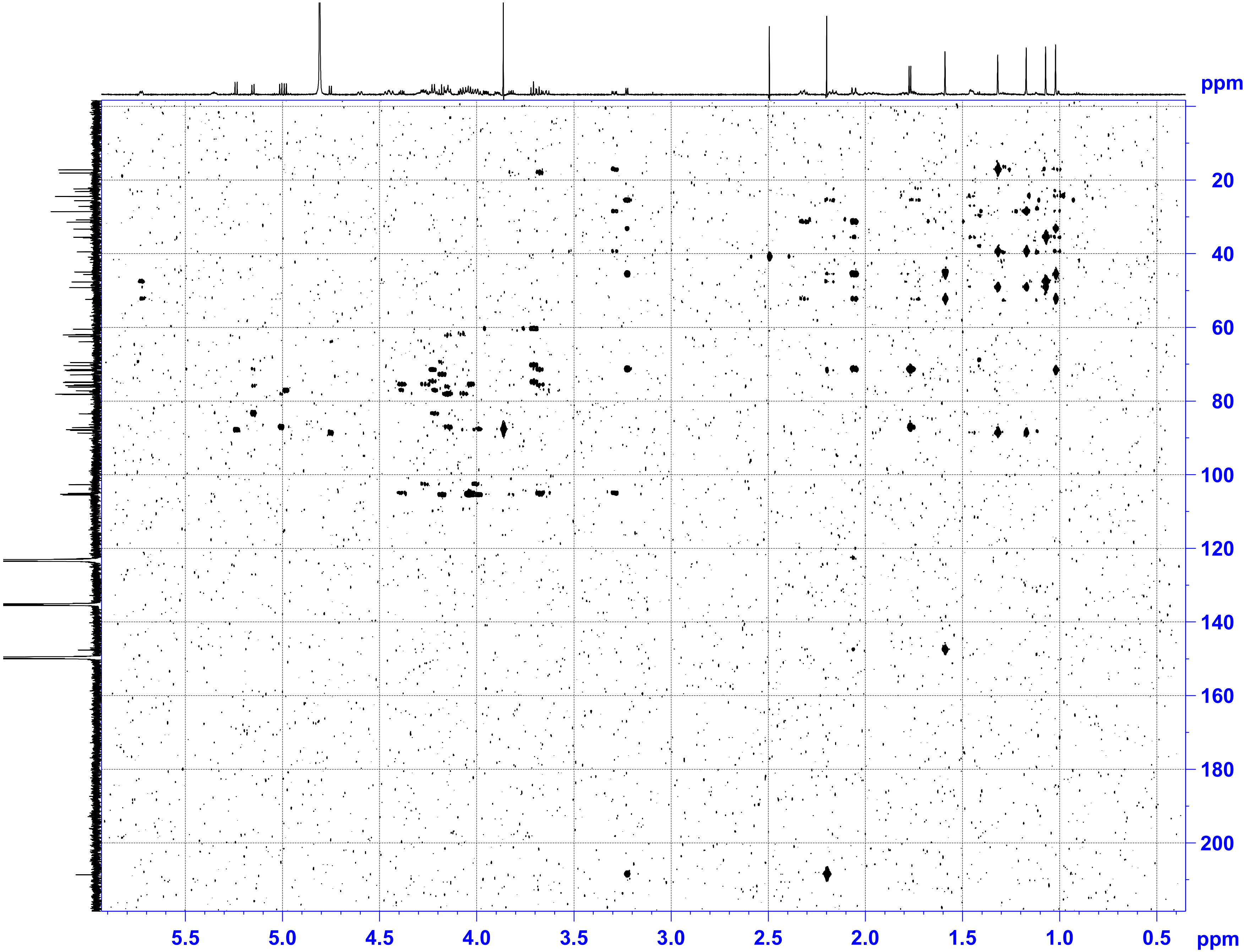

Figure S81. The HMBC (700.00 MHz) spectrum of DS-kuriloside M (**11**) in C_5D_5N

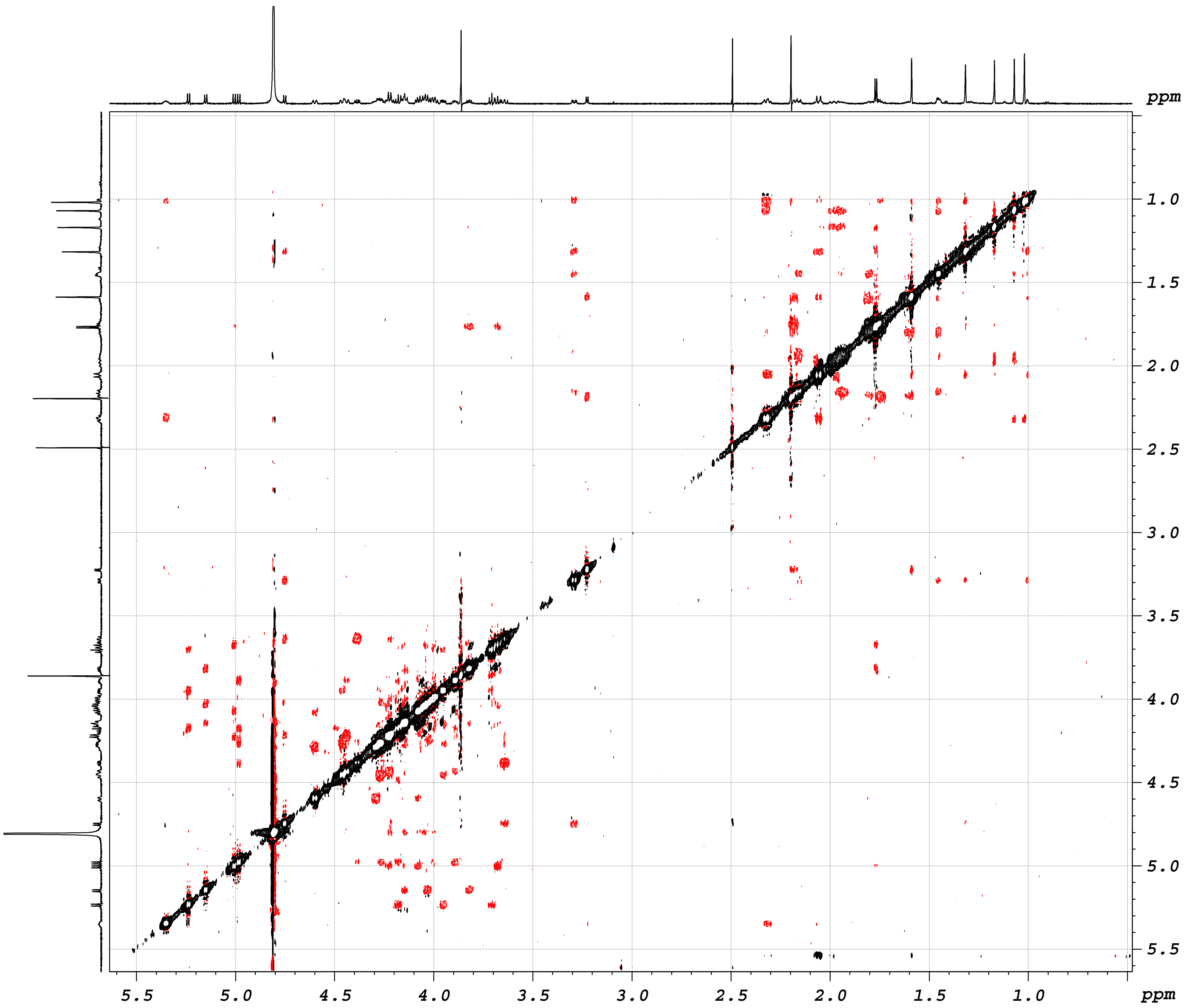

Figure S82. The ROESY (700.00 MHz) spectrum of DS-kuriloside M (**11**) in C_5D_5N

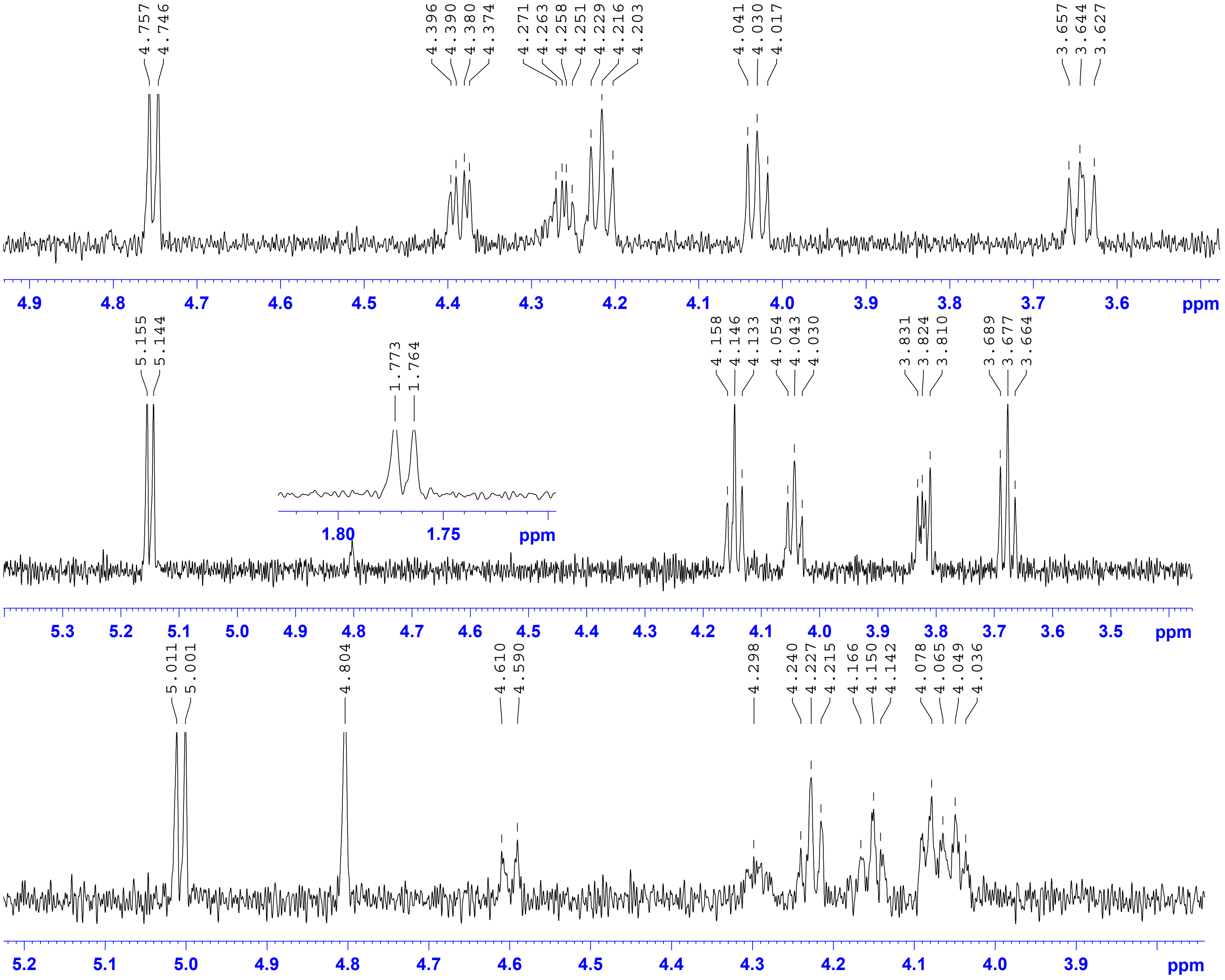

Figure S83. 1 D TOCSY (700.00 MHz) spectra of XyloseI, QuinovoseII and GlucoseIII of DS-kuriloside M (**11**) in C_5D_5N

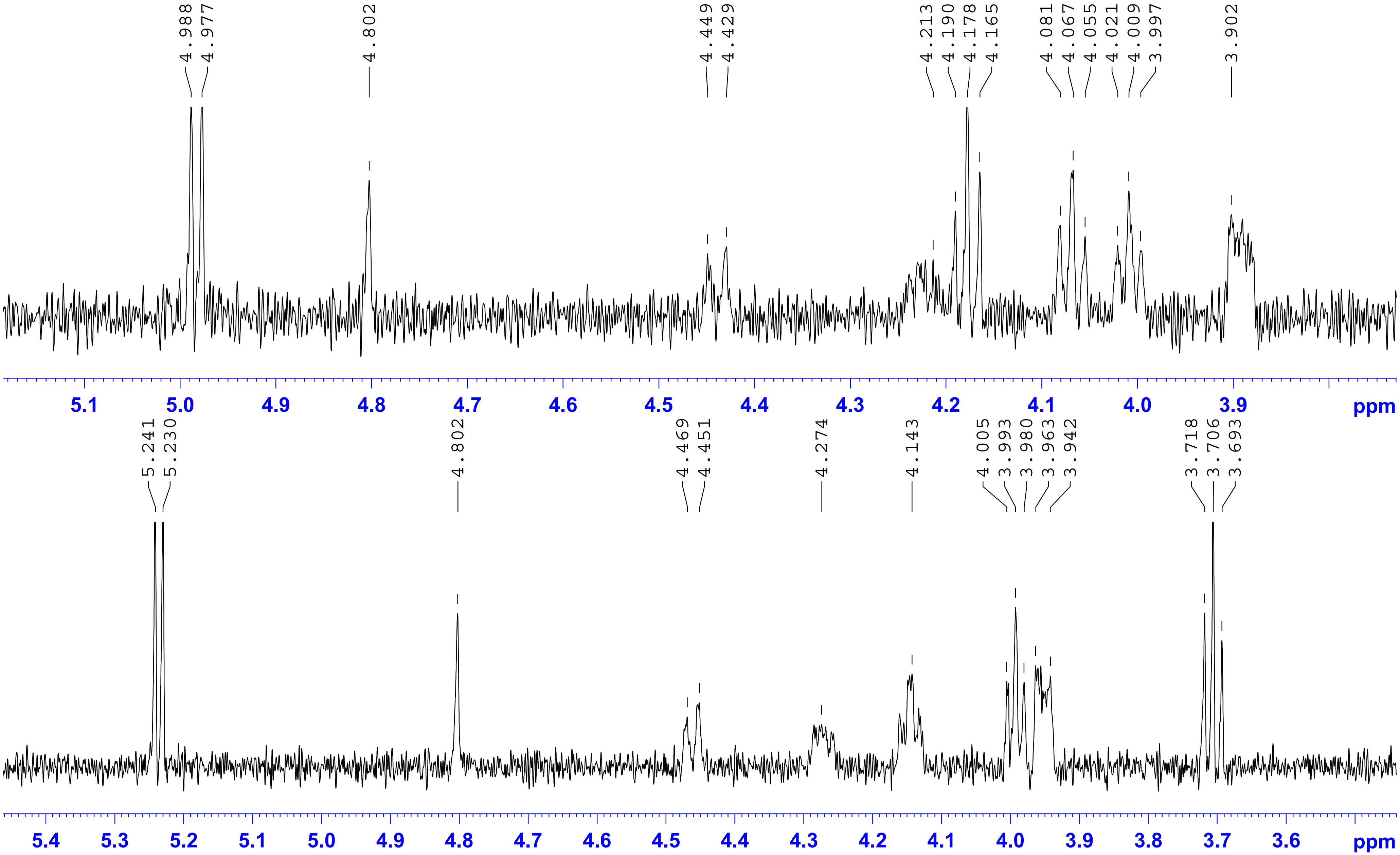

Figure S84. 1 D TOCSY (700.00 MHz) spectra of GlcIV and MeGlcV of DS-kuriloside M (**11**) in C_5D_5N

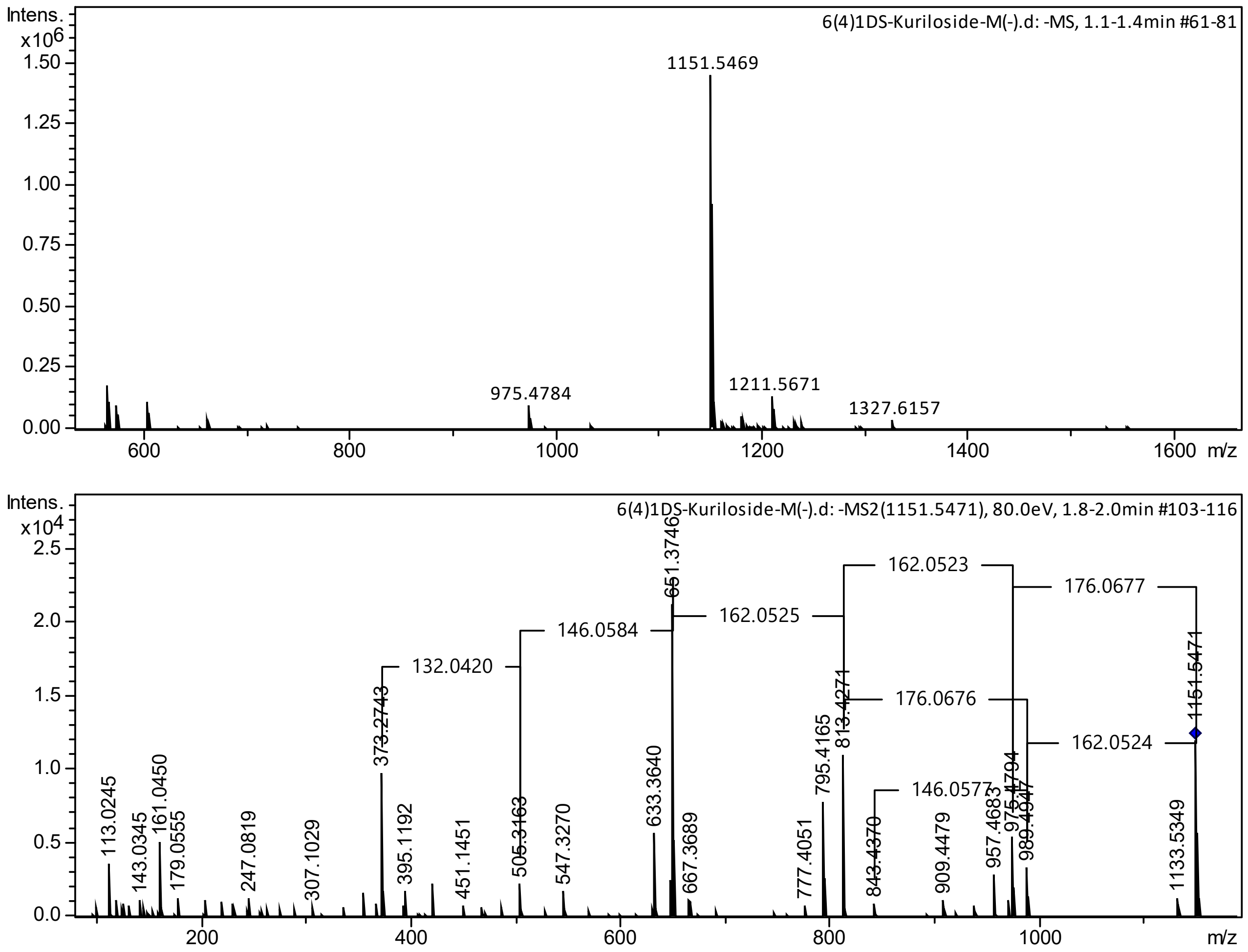

Figure S85. HR-ESI-MS(−) and ESI-MS/MS spectra of DS-kuriloside M (**11**).
